# Optimisation of trace mineral supplementation in diets for Atlantic salmon smolt with reference to holistic fish performance in terms of growth, health, welfare, and potential environmental impacts

**DOI:** 10.3389/fphys.2023.1214987

**Published:** 2023-08-17

**Authors:** Marialena Kokkali, Lene Sveen, Thomas Larsson, Aleksei Krasnov, Alexandros Giakovakis, John Sweetman, Philip Lyons, Katerina Kousoulaki

**Affiliations:** ^1^ Department of Nutrition and Feed Technology, Nofima, Bergen, Norway; ^2^ Department of Fish Health, Nofima, Ås, Norway; ^3^ Department of Biological Science, University of Bergen, Bergen, Norway; ^4^ Alltech Inc., Meath, Ireland

**Keywords:** trace minerals, Atlantic salmon, stress, low fish meal diet, fish health, welfare, environmental impact

## Abstract

The aquafeed ingredient inventory is ever changing, from marine to plant based, and recently evolving to incorporate increasing amounts of low trophic, side stream and circular economy based raw materials, each one contributing with variable amounts and qualities of macro- and micronutrients. Meeting the micronutrient requirement of farmed fish for healthy and efficient growth under normal and challenging conditions is of paramount importance. In this study we run a trial based on a 2 × 4 factorial design with three replications for each dietary treatment, where *Atlantic salmon* smolt were fed one of 8 experimental diets supplemented with either organic or inorganic mineral premixes (copper, iron, manganese, selenium, and zinc) at four dietary inclusion levels. We saw a trend for higher growth rate in the organic mineral groups irrespective of the dietary mineral levels. Mineral digestibility was negatively correlated with increasing mineral supplementation levels for all tested minerals but Se which increased with the increasing supplementation in the inorganic and up to the 2nd inclusion level in the organic mineral groups. Increasing mineral supplementation affected retention efficiency of Zn, Mn, Cu and Fe while mineral source affected only the retention of Se which was higher in the organic mineral groups. Moreover, fish obtained higher EPA and DHA in their body and increased slaughter yield in the organic as compared to the inorganic mineral groups and corroborated that trace mineral inclusion levels play a key role on salmon fillet’s technical quality. More effects from different origin and dietary inclusion levels of trace minerals were seen on fillet yield, fillet technical and nutritional quality, bone strength, skin morphology, organ mineralization and midgut transcriptome.

## 1 Introduction

Responsible aquaculture growth requires practices adjusted to evolving knowledge based industrial standards that can secure good fish welfare, health, and low environmental impacts, alongside with the production of safe and nutritious consumer products and the economic sustainability of the business. Modern salmon feeds are based on 70% plant ingredients, a reverse picture to the marine based aquafeeds used a few decades ago consisting mainly of fish meal, and fish oil (Aas et al*.* 2019). Feeding with plant-based diets often results in negative effects on fish performance, health, and welfare, such as lower feed intake rates and growth, lipid accumulation in internal organs, intestinal inflammation, and higher susceptibility to diseases (Krogdahl et al., 2010; Torstensen et al., 2011; Burr et al., 2013). Counteracting the shortcomings of plant-based diets as compared to fish meal and fish oil, dietary supplementation levels of micro-ingredients in contemporary salmon feeds, such as essential amino acids added in crystalline form, trace minerals, vitamins, phosphorous sources and astaxanthin, have quadrupled since 1990 (Aas et al*.* 2019).

When it comes to the essential trace minerals copper (Cu), manganese (Mn), selenium (Se), iron (Fe), and zinc (Zn), common commercial praxis is to supplement aquafeeds with trace mineral in their inorganic forms to cover the requirements of farmed fish, thus the relative levels of naturally occurring and added ones will vary in diets changing from marine to plant based with largely unknown consequences for fish physiology. There is increasing evidence that different trace mineral sources have different bioavailability (Maage and Sveier, 1998; Standal*.* 1999) and physiological effects (Berntssen et al*.* 2018).

Trace minerals are involved in a great number of physiological processes including respiration, protein and lipid metabolism, and cell redox regulation (Lall, 2022). Cu is an essential trace element for all animals including fish (Tan et al*.* 2011), functioning as catalytic and structural cofactor in multiple enzymes involved in energy production (Damasceno et al*.* 2016), Fe acquisition (Watanabe et al*.* 1997), oxygen transport (Olmedo et al., 2013), and antioxidant and immune activity (Tang et al*.* 2017). Cu supplementation has been found to improve growth and immunological function in different fish species (Berntssen et al*.* 2000; Lin et al*.* 2008; Lin et al., 2010; Sun et al*.* 2013; Mohseni et al*.* 2014). Mn is an active component of fish metabolic regulation, bone, and fillet formation, as well as fish growth (Prabhu et al., 2019b; Lall, 2022) and supplementation is required to meet fish needs (Prabhu et al., 2019b; Silva et al., 2019). Mn and Cu are functional catalytic sites of the antioxidant enzymes superoxide dismutase (SOD) while Se of the glutathione peroxidase (GPX), respectively, where SOD handles superoxide and GPX handles both lipid and water-soluble hydroperoxides (Esworthy et al., 1998; Lygren et al., 1999). A GPX situated in the intestinal mucosa specifically handles fatty acid hydroperoxides from the diet and converts them to non-toxic hydroxyl fatty acids (Esworthy et al., 1998). Apart from its peroxidation role Se, plays a key role against heavy metal, i.e., cadmium (Cd) and mercury (Hg)) toxicity (Watanabe et al., 1997; Bjerregaard, 2011), cellular redox linked to health status and disease prevention of the fish (Lall, 2022), as it is an essential component of selenoproteins involved in free radical metabolism and immune responses (Labunskyy et al., 2014). Fe is a component of heme proteins like hemoglobin and myoglobin (Lall, 2022), and enzymes like peroxidase, catalase, and cytochromes (Lim et al., 2001); and plays an important role in various biochemical processes from gene regulation, binding, and transportation of oxygen to regulation of cell growth (Lim et al., 2001; Lall, 2022). Fe absorption is a complex procedure and is affected by Fe availability, form, and other dietary components like phytic and ascorbic acid (Lall, 2022); thus, Fe supplementation is necessary, especially when fish meal is depleted in the diets, to maintain fish health and optimal growth (Lim et al., 2001; Lall, 2022). Zn has a catalytic role for numerous enzymes, aids intra and extra cellular metabolic functions as well as formation of hormones with diverse roles (i.e., growth, reproduction, immunity, etc.) (Dawood et al., 2021; Lall, 2022). Zn supplementation has a positive effect on aquatic animal’s performance, immunity, antioxidant status as well as feed utilization and digestibility (Dawood et al., 2021). Fe and Zn, among other components, function as co-factors or coenzyme precursors for essential long chain n-3 polyunsaturated fatty acid (LC *n*–3 PUFA) biosynthesis (Lewis et al., 2013) and play also role in the bioconversion of alpha-linolenic acid (ALA) to eicosapentaenoic acid (EPA), and docosahexaenoic acid (DHA) in salmonids (Mahfouz et al., 1989; Eder and Kirchgessner, 1994; Stangl and Kirchgessner, 1998; Zhou et al., 2011). They also play essential role in lipid metabolism as co-factors and co-enzymes in enzymes involved in peroxisomal β-oxidation (Osumi and Hashimoto, 1979; Poirier et al., 2006). There is a trend in nutrition of farmed land animals, such as poultry and swine, to replace inorganic trace mineral sources with lower amounts of putative more bioavailable organic trace mineral sources resulting in decreased excretion of minerals in the environment, but also improved in trace mineral-associated functionalities (Abdallah et al., 2009). Mineral uptake in fish is highly correlated with diet composition, the chemical form of the mineral, and the interactions with other diet components (i.e., phytic acid) (Kumar et al., 2012; Silva et al., 2019). In fish too, there is an increasing interest in comparing the bioavailability of organic and inorganic minerals in fish diets, but the available data are still scarce and inconsistent (Prabhu et al., 2016; Dominguez et al., 2017). In a meta-analysis by Prabhu et al. (2016), it was highlighted that organic forms of Se, like selenomethionine (SeMet) and selenoyeast (Se yeast), are more bioavailable compared to selenite; however, for other trace minerals like Zn and Mn the results of the different studies are conflicting (Prabhu et al., 2019; Silva et al., 2019; Meiler et al., 2021). Li et al. (2019) saw different metabolic patterns, including increased expression of the peptide transporter gene PepT1 and decrease in specific divalent mineral transporter gene CTR1 when porcine intestinal epithelial model cells (IPEC-J2) were provided media with increasing levels of different (inorganic vs*.* proteinates) Cu sources.

Supplemental sources of organic minerals are biotechnologically produced ingredients, which may also have variable performance. Keenan et al. (2019) showed improved metabolic performance in intestinal cell lines HT29 and Caco-2 providing Cu as Cu proteinate, against other forms, as for instance Cu-Gly, CuSO_4_, and Cu organic acid chelate. Negative effects of the latter were associated with sustained ROS and protein misfolding which led in turn to generation of aggresomes. Thus, apart from the pronounced interaction of mineral uptake and fish health and performance, the underlying mineral uptake mechanisms and trace mineral related physiological mechanisms in current farming practices require more in-depth investigation.

The main goal of this study was to map the physiological implications of supplemental dietary essential trace minerals in farmed Atlantic salmon (*Salmo salar*). Emphasis is placed on the release rate to the environment and importance for fish welfare, health and performance related to differential uptake, tissue assimilation and function mechanisms involved when the essential trace minerals Zn, Cu, Mn, Fe, and Se, are provided at different dietary levels and forms, namely, organic or inorganic.

## 2 Materials and methods

### 2.1 Trial feed design

A 2 × 4 factorial design (dietary trace mineral type organic vs*.* inorganic x 4 dietary mineral levels) with 3 replications for each dietary treatment was applied. The test organic (OM) and inorganic (IM) mineral premixes used in the experimental diets contained Cu, Fe, Mn, Se, and Zn. For Cu, Mn, and Zn, mineral premixes were created to formulate diets that would contain ¼, ½, ¾ or 4/4 of the difference between the respective levels in a non-supplemented dietary mix and the maximum allowed supplementation levels in fish feeds (European Commission, 2014). For Fe, 550 ppm was used as max reference dietary level, and not 750 ppm, which is the legal limit, as Fe has known prooxidative effects at high dietary levels, reported also in Atlantic salmon (Andersen et al., 1997). In the case of Se, we added 0.05, 0.1, 0.15, and 0.2 ppm in the respective diets (Table 1). The organic minerals used were Bioplex Cu, Mn, Zn and Fe and Selplex provided by Alltech Inc (Dunboyne, Ireland) and the inorganic ones used were sulphates. All dietary formulations included 10% fish meal, vitamins, and other functional components at equal amounts (Supplementary Table S1).

**TABLE 1:** Trial design target mineral supplementation levels (SL) in the experimental diets.

Treatment	Basic raw material mix^a^	1st (SL)^b^	2nd (SL)^b^	3rd (SL)^b^	^c^4th (SL)^b^
Cu mg/kg	6.4	10	15	20	25
Fe^d^ mg/kg	280	348	415	483	550
Zn mg/kg	57	88	119	149	180
Mn mg/kg	42	57	71	86	100
Se^e^ mg/kg	0.800	0.850	0.900	0.950	1.000

^a^ Analysed values.

^b^ Calculated values.

^c^ Diets were planned to contain Cu, Zn and Mn at EU, max allowed mineral levels.

^d^ Dietary Fe levels in diets with highest mineral supplementation were sat at 550 ppm.

^e^ Se was supplemented at 0.05 ppm, 0.1 ppm, 0.15 ppm, and 0.2 ppm on top of the pre-existing levels.

The raw materials used in feed production and the basic raw material mix used for the production of all the experimental dies were analysed for their content in the variables in this study to calculate the amounts of minerals that should be added to end up with the desired dietary mineral levels. The experimental diets were produced by extrusion using a Wenger TX-52 co-rotating twin-screw extruder with 150 kg h^-1^ capacity. The settings of the extruder were “normal” i.e., the production can be up scaled to a feed factory. Following production, the experimental feeds were analysed for total lipids, fatty acid profile, crude protein, energy, water, P, Cu, Mn, Zn, Fe and Se. Dietary Fe was found to be somewhat lower and dietary Se higher than expected (Table 2). However, as Fe levels were still withing the legal limits and Se levels within the range present in higher fish meal diets it we decided to use the produced diets in the planned feeding trial with Atlantic salmon smolt.

**TABLE 2:** Chemical composition of the experimental diets used in the feeding trial (Complete diet chemical composition in Supplementary Table S2).

Diet name		IM1	IM2	IM3	IM4	OM1	OM2	OM3	OM4
Copper^a^	mg/kg	9.3	13	19	24	9.7	14	19	24
Iron^b^	mg/kg	300 ± 4	386 ± 22	458 ± 9	509 ± 12	302 ± 5	374 ± 13	420 ± 13	502 ± 45
Zink^b^	mg/kg	78 ± 6	107 ± 11	133 ± 13	161 ± 15	87 ± 7	118 ± 11	149 ± 17	176 ± 19
Manganese^b^	mg/kg	57 ± 6	66 ± 4	80 ± 4	99 ± 3	55 ± 2	71 ± 2	83 ± 3	96 ± 3
Selenium^b^	mg/kg	1.20 ± 0.05	1.30 ± 0.07	1.46 ± 0.13	1.47 ± 0.06	1.36 ± 0.08	1.38 ± 0.04	1.45 ± 0.08	1.51 ± 0.05
Selenium^a^	mg/kg	0.7	0.9	1.2	1.1	1.0	1.2	0.9	1.0
Total P^c^	%	1.2	1.2	1.2	1.2	1.1	1.1	1.2	1.2
Yttrium^a^	mg/kg	77	75	80	78	74	79	83	81
Free astaxanthin^c^	mg/kg	40	42	42	40	40	40	40	40
Moisture^c^	%	7.8	6.4	6.8	7.2	7.5	7.7	7.5	7.3
Protein^c^	%	43.3	43.9	43.5	43.1	43.1	43.5	43.4	43.8
Fat^c^	%	28.9	29.7	29.0	28.8	29.7	29.3	29.0	29.0
Energy^c^	KJ/g	23.08	23.33	23.06	23.4	23.06	23.28	23.49	23.11

^a^ Analysed at Eurofins WEJ, contaminants, Hamburg, Germany (*n* = 1).

^b^ Analysed at Alltech Inc, Dunboyne, Ireland (*n* = 3).

^c^ Analysed at Nofima’s Accredited laboratory Biolab, Bergen, Norway (*n* = 2).

### 2.2 Salmon feeding trial

The salmon feeding experiment took place in the land tank facilities of Nofima at Sunndalsøra, Norway and lasted for 12 weeks. The fish used were non-vaccinated Atlantic salmon smolt. At trial start, fish were starved for 24 h and then the individual weight and length of 100 fish were noted and fish of the 10% highest and lowest size groups were excluded. Fish showing deformities or lesions were also excluded. In total, 55 fish were distributed in each one of the 24 experimental tanks (0.5 m^3^ volume per tank) and each one of the 8 experimental diets was randomly attributed to three of them. The fish had 215 ± 1.36 g body weight at start and more than tripled that during the 83-day-long trial, resulting at a mean final body weight, across the experimental treatments, of 702 ± 28.15 g. Feeding was continuous using automatic feeders. The fish were fed, first the day following start sampling, gradually increasing amounts to determine the satiation feeding rate levels of each group. Uneaten feed was collected and weighed daily for the estimation of satiation feed intake levels of each one of the experimental fish populations and the daily distributed feed amounts were set at 120% of this amount.

The tanks were equipped with continuous light and flow-through water systems using UV-treated filtrated sea water from 40 m depth (flow rate of approximately 20 L/min). Oxygen levels were monitored daily, and water flow adjusted, when necessary, to achieve optimal oxygen saturation (∼90%) levels for the first 9 weeks of the experiment and suboptimal oxygen saturation levels between (70%–80%) for the last 3 weeks of the experiment, equally among the experimental tanks. The mean water temperature during the trial was 8.73^o^C.

**FIGURE 1:**
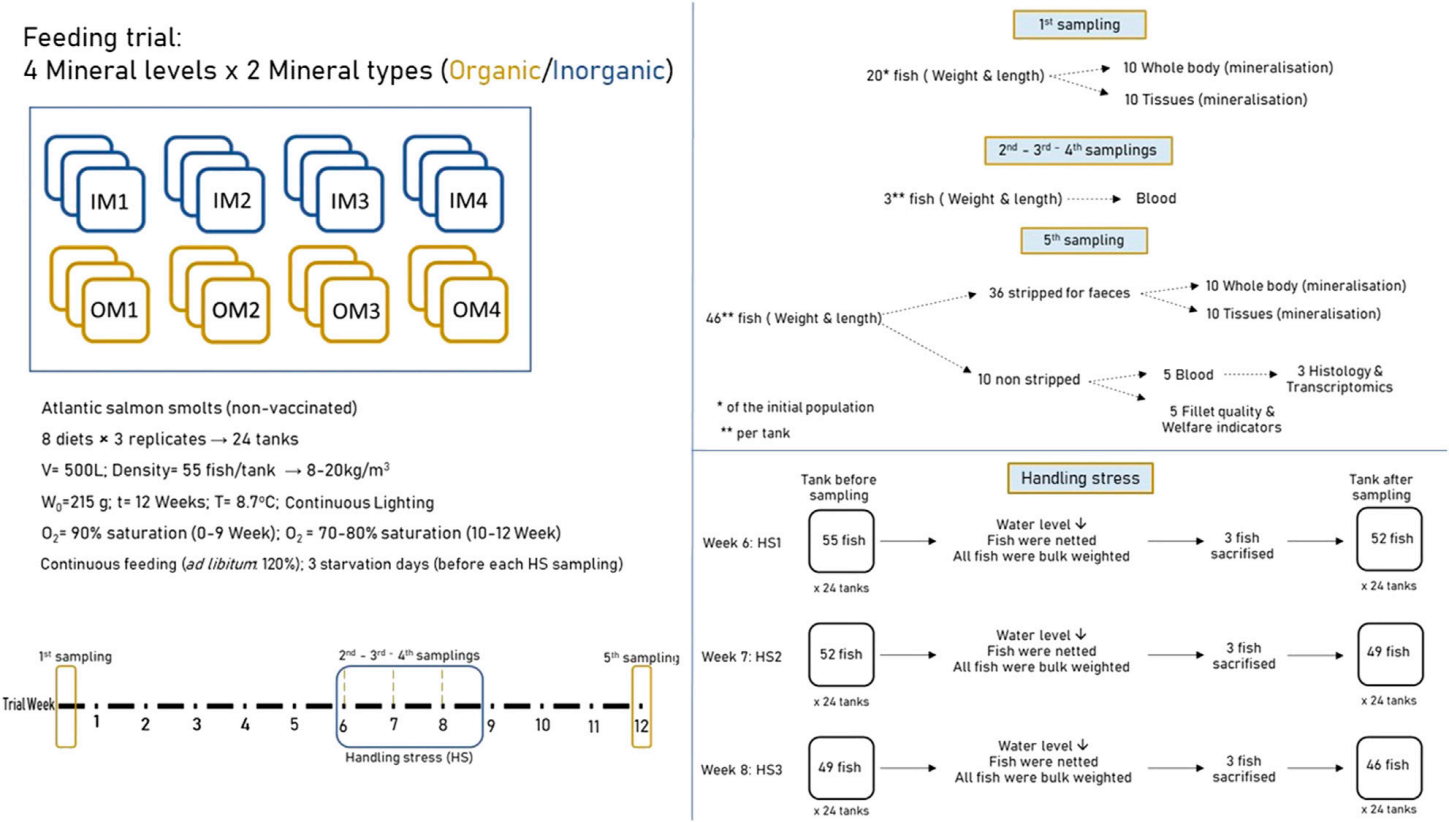
Schematic representation of the experimental design and set up.

Following 6 weeks of conditioning to the experimental diets, fish were subjected to handling stress treatments, once a week, for three consecutive weeks (trial week 6, 7, and 8). The handling stress treatments were as follows: tank water level was reduced, fish were chased, netted, weighted, and reintroduced to the tanks. The day before each handling stress sampling fish were starved, and normal feeding was carried on after the treatments. Following the last handling stress treatment (week 8), the fish were fed continuously and undisturbed until the final sampling (week 12), when they were individually weighed and sampled for different tissues. A schematic overview of the trial setup and the samplings is shown in Figure 1.

### 2.3 Samplings

In total, 5 samplings took place during the 12 trial weeks. The first sampling took place at trial start, in which 20 fish from the total population were sacrificed for collecting samples of whole body (from 10 fish) and different organs (gill, skin, spleen, head kidney, fillet from the remaining 10 fish) to be analysed for their mineral composition and used as start point for further calculations. Three intermediate samplings at the end of experimental weeks 6, 7, and 8 were performed in which all fish per tank were bulk weighed and 3 fish from each tank were sacrifised and sampled for blood; welfare indicators were also monitored for diagnosing and classifying external injuries according to Noble et al. (2018). Before bulk weighing fish were mildly anesthetized with dilute benzocaine solution. Total 9 fish were sacrificed per tank during the 3-handling stress samplings and, 46 fish remained in each tank after week 8 to the end. The last sampling was at trial end when all fish were euthanised by an overdose of benzocaine solution, individually weighed and measured for standard length. Of those, 36 fish were stripped for faeces, which were separated from urine and collected in one box per tank and frozen at–25^o^C until further analysis. From the stripped fish from each tank, 10 were used for chemical analyses in whole body, and 10 for analyses in different organs (gill, skin, liver, spleen, head kidney, and fillet); the remaining 26 fish were discarded. Of the 10 fish from each tank that were not stripped; 5 were used for blood sampling, and 3 for histology and gut transcriptome analysis. The remaining 5 were left to bleed out cutting their gills, re-weighted after bleeding, gutted, measured individual liver weight, and re-weighted and scored for welfare indicators. The gutted fish were stored on ice in Styrofoam boxes and shipped to Nofima Ås, Norway, where they were stored at 1 °C and analysed for fillet technical quality, 7–8 days post-mortem.

The experimental feeds, faeces, and fish whole body and organs (from 10 fish at start and 10 fish per tank in the end) were analysed for the 5 test minerals (Cu, Fe, Zn, Mn, Se), Ca, P, Mg, Na, K and Y. In addition, whole body samples were analysed for fatty acid profile, and fillets were analysed for astaxanthin. Fish samples (and faeces) were freeze dried before analysis.

### 2.4 Blood chemistry and antioxidant status

Blood samples were taken from the caudal vein of 3 fish from each tank during the handling stress (HS) treatments (HS1, HS2 and HS3) and final sampling. Photometric analyses were used to determine the blood serum content in alanine aminotransferase (ALAT), aspartate aminotransferase (ASAT), creatine kinase (CK), protein, glucose, cortisol, potassium level (K+), triglycerides and cholesterol, as indicators of the nutritional status of fish using a Pentra C400 HORIBA (HORIBA Medical, Montpellier, France).

### 2.5 Histology

At trial end, skin samples were taken from 3 fish per tank to evaluate general structure and mucous cell density. Tissue samples were stored in 10% formalin pots (CellStore™ 20 mL Pots, CellPath). Embedding, sectioning, and staining of the tissue samples were done at the Norwegian Veterinary Institute in Harstad, Norway. In brief, the tissue sections were hydrated in water and stained with 1% Alcian blue (Alfa Aesar), 3% acetic acid for 15 min, transferred to 1% periodic acid (VWR) for 10 min, followed by Schiffs (Sigma-Aldrich^®^) reagent for 15 min, 30 s in hematoxylin (VWR) before dehydration and mounting. The stained tissue sections were scanned with a Hamamatsu slide scanner (Hamamatsu) and uploaded to the Aiforia^®^ platform and analysed according to Sveen et al. (2021).

### 2.6 Gut transcriptome

Mid gut samples of 3 fish per tank (final sampling), were immersed in RNALater (Sigma-Aldrich^®^) and stored in −20°C until further use. Transcriptomic analysis was performed in Nofima’s fish health laboratories in Ås, Norway. The DNA oligonucleotide microarray platform developed by Nofima (SIQ-6 microarray: Salmon Immunity and Quality) contains probes for 15 K genes annotated with the bioinformatic pipeline STARS, (Krasnov et al*.* 2011). Microarrays were fabricated by Agilent Technologies, equipment and reagents unless indicated otherwise were purchased from the same provider. Total RNA was extracted with PureLink RNA Mini Kit (Thermo Fisher Scientific) and RNA quality confirmed (Bioanalyzer). One-color (Cy3) hybridization was performed. Following scanning of arrays, data were processed using STARS. Data were submitted to NCBI GEO Omnibus.

### 2.7 Fish welfare and filet quality

Welfare indicators (cataract, fin and skin damages, liver color, and visceral fat content) from 10 fish from each tank were assessed, and the same fish were then used to evaluate skin and bone strength as well as filet technical quality (melanin spots, fillet gaping, fillet and skin color, fillet firmness). The analyses were performed in Nofima’s nutrition and feed technology laboratory in Ås, Norway. In detail, 7–8 days post-mortem, fish were filleted (one side only) manually by an experienced technician. The fillet was then weighed, and evaluated for gaping (i.e., slits and holes in the fillet; score 0–5; Andersen et al*.* 1997), fillet colour by SalmoFan (DSM, Heerlen, Netherlands) and Minolta Chroma meter (two measurements per fish; CR-400 Minolta, KONICA MINOLTA SENSING, INC. JAPAN) on the dorsal part of the Norwegian quality cut (NQC), and muscle firmness (instrumentally; TA-XT-2, Stable Micro Systems Ltd. (SMS), Surrey, England) in the dorsal part of the NQC by pressing a cylinder (12.7 mm diameter) at constant speed (1 mm/s) into the muscle (Mørkøre and Einen 2003), perpendicular to the muscle fibres. Fillet muscle pH was determined in the dorsal, anterior part of the fillet using a pH meter (330i, Wissenchaftlich-Techniche Werkstätten GmbH (WTW), Weilheim, Germany) connected to an electrode (BlueLine 21, Schott Instruments Electrode, SI Analytics GmbH, Mainz, Germany). From the remaining carcass of the fish, the NQC cutlet, including back bone, was removed, and used for evaluation of skin color by Minolta just above the lateral line (two measurements per fish). For this purpose, we used the tristimulus CIE *L* ^∗^ *a* ^∗^ *b* ^∗^ 1976 color space, where the *L* ^∗^ variable represents lightness (*L* ^∗^ = 0 for black, *L* ^∗^ = 100 for white) and *a* ^∗^ and *b* ^∗^ indicate color directions: *a* ^∗^ is the red direction, −*a* ^∗^ is the green direction, +*b* ^∗^ is the yellow direction, and −*b* ^∗^ is the blue direction. Instrumental (TA-XT-2) skin puncture strength was evaluated by a needle (P/2N, SMS) inserted three scales above the lateral line of the fish (two measurements per fish). The backbone was then removed and cleaned for muscle remains and analysed for bone strength instrumentally (TA-XT-2) by pressing a rectangular knife (HDP/WBR, SMS), positioned perpendicular to back bone, at constant speed (2 mm/s) into the center of a vertebrae until a depth of 70% of vertebrae thickness (two measurements per fish). The fresh fillets were packed in zip lock bags and frozen individually at −20 C°. After 18 days, the fillets were thawed at room temperature, evaluated once more for fillet gaping, then dabbed with paper and weighed to obtain liquid loss gravimetrically compared to weight of fresh fillet.

### 2.8 Chemical analysis of diets, faeces, whole fish, and organs

Sample nitrogen (N) content was determined by the Kjeldahl method (ISO 5983–2 2009), and crude protein was estimated based on N × 6.25. Moisture was determined by drying at 103°C (ISO 6496-2 1999). Fat content was determined by chloroform-methanol extraction (Bligh and Dyer 1959). Fillet astaxanthin was analysed using a method which determines the content of astaxanthin-esters in aquatic animals known to only contain carotenoids in the form of astaxanthin esters. The method is also used to determine any content of free trans-, 9cis- and 13cis-astaxanthin (Schüep and Schierle, 1995). For mineral content analysis, sample preparation was performed by microwave-assisted digestion using a single reaction chamber oven (UltraWave^™^, Milestone, Sorisole, Italy) equipped with 22 position rack. More in detail, 0.15–0.25 g of finely ground sample was dissolved in 1 mL MilliQ water (passed through a Millipak filter, 0.22 μm), and 2 mL of nitric acid (69% HNO₃, VWR Chemicals, AnalaR NORMAPUR^®^ ACS, Reag. Ph. Eur. Analytical reagent) were added. The UltraWave^™^ protocol was run at 1500 W of microwave irradiation and a maximum temperature and pressure of 220 °C and 110 bar, respectively, and a base load of 130 mL MilliQ water and 5 mL HNO₃ 69%. Inductively Coupled Plasma Optical Emission spectroscopy (ICP-OES) (Agilent 5110 VDV, Agilent Technologies, Mulgrave, Australia) was used for determination of element (Cu, Fe, Mn, Zn, K, Mg, Na, Ca, Y) concentration. For plasma generation, nebulization and auxiliary gas, argon (Linde Gas As, Oslo, Norway) with a purity of 99.996% was used. The conditions used for element determination by ICP-OES were in accordance with the NS: EN 15621:2017 method adapted for OES. Selenium was analysed at an external laboratory (Eurofins, Molde, Norway).

### 2.9 Calculations

To evaluate the mineral, macronutrient, and energy apparent digestibility coefficient (ADC), 0.01% yttrium oxide (Y) marker was added in the experimental diets (Supplementary Table S2). The ADC of nutrients and energy, fish performance, survival, feed intake and efficiency as well as different fish biometrics were calculated, using the following equations:

$$ADC\;of\;nutrient\;\left(\%\right)=100-\left(\frac{Y\;in\;diet\times Nutrient\;in\;faeces}{Y\;in\;faeces\times Nutrient\;in\;diet}\times 100\right)$$

$$Feed\;conversion\;rate\;\left(FCR\right)=\frac{feed\;consumed}{weight\;gain}$$

$$Specific\;growth\;rate\;\left(SGR\right)=100\times \frac{\left(\ln\left(W2\right)-\ln\left(W1\right)\right)}{feeding\;days}\;*$$

Thermal growth coefficient TGC=1000×W213−W113∑(TCo×feeding days**

$$Slaughter\;yield\;\left(\%\right)=100\times \frac{gutted\;fish\;weight}{whole\;fish\;weight}$$

$$Fillet\;yield\;\left(\%\right)=100\times \frac{{Fillet\;weight}_{right}+{Fillet\;weight}_{left}}{gutted\;fish\;weight}$$

$$Hepatosomatic\;index\;\left(HSI\right)=100\times \frac{liver\;weight}{fish\;weight}$$

$$Condition\;factor\;\left(C_{f}\right)=100\times \frac{fish\;weight}{{fish\;fork\;length}^{3}}$$

^*^ W1= weight in the beginning of the period, W2 = weight in the end of the period.^**^ Cho, 1992.

### 2.10 Statistics

The biological and analytical data were subjected to two-way analysis of variance (ANOVA) using R.4.2.3, R Core Team (2020) and SPSS 29.0 for Windows. When significant differences among groups were identified, multiple comparisons among means were made using Duncan’s *post hoc* test. Differential gene expression was assessed by criteria: fold change >1.75 and *p* < 0.05. Treatment effects are considered at a significance level of *p* < 0.05 and tendencies at 0.1 < *p* < 0.05.

## 3 Results and discussion

### 3.1 Performance

Fish in the OM showed significantly higher growth rate as compared to the IM treatments halfway through the trial, at weeks 6 (1^st^ handling stress) and 7 (2nd handling stress) (*p* < 0.05), and a tendency for higher growth rate at week 8 (3^rd^ handling stress) and overall (0.10 < *p* < 0.05) at the end of the trial (week 12th handling stress) (Table 3 and Supplementary Table S3). Accordingly, total feed (in dry matter; DM) consumed per fish at trial end, tended (*p* < 0.08) to be higher in the OM as compared to the IM groups. Due to oxygen restriction (by design) and tank size limitation, we believe that the performance of the originally faster growing groups (OM) was hindered, limiting the initial differences created between OM and IM treatments towards the end of the experiment. Similarly, dietary organic mineral supplementation compared to inorganic, has shown a positive effect on Nile tilapia’s (*Oreochromis niloticus*) performance and feed efficiency as well as FCR and protein efficiency ratio (PER) (El-Sayed et al., 2023).

**TABLE 3:** Atlantic salmon trial start and end body weight (BW) growth and feed utilisation performance when fed diets (8 treatments (T)) with variable source (S) and levels (L) of trace minerals (values are means ± standard variation (a); *n* = 3 tanks)–(For all studied variables see Supplementary Table S3).

										*p*-values^a^			
		IM1	IM2	IM3	IM4	OM1	OM2	OM3	OM4	T	S	L	S x L
Start BW (g)	(g)	216 ± 1	214 ± 0	215 ± 1	216 ± 1	215 ± 1	216 ± 2	215 ± 2	216 ± 1	n.s.	n.s.	n.s.	n.s.
BW week 6	(g)	370 ± 8	370 ± 1	365 ± 19	361 ± 8	377 ± 5	385 ± 5	374 ± 12	372 ± 6	n.s.	0.016^b^	0.279	0.896
Final BW	(g)	680 ± 45	693 ± 2	697 ± 47	694 ± 19	702 ± 23	730 ± 30	710 ± 27	708 ± 10	n.s.	0.087^b^	n.s.	n.s.
TGC week 6		3.14 ± 0.17	3.20 ± 0.02	3.08 ± 0.31	3.01 ± 0.15	3.31 ± 0.07	3.42 ± 0.12	3.23 ± 0.17	3.19 ± 0.10	n.s.	0.013^b^	0.217	0.986
Total SGR		1.38 ± 0.08	1.41 ± 0.00	1.41 ± 0.08	1.41 ± 0.04	1.43 ± 0.04	1.47 ± 0.05	1.44 ± 0.04	1.43 ± 0.02	n.s.	0.082^b^	n.s.	n.s.
Total TGC		3.85 ± 0.27	3.95 ± 0.01	3.96 ± 0.26	3.94 ± 0.13	4.00 ± 0.14	4.15 ± 0.17	4.04 ± 0.13	4.02 ± 0.07	n.s.	0.083^b^	n.s.	n.s.
Total feed intake DM/fish	(g)	270 ± 9	268 ± 16	265 ± 25	261 ± 9	273 ± 13	285 ± 18	272 ± 13	274 ± 7	n.s.	0.080^b^	n.s.	n.s.
Total FCR DM		0.58 ± 0.05	0.56 ± 0.03	0.55 ± 0.00	0.55 ± 0.01	0.56 ± 0.00	0.56 ± 0.01	0.55 ± 0.00	0.56 ± 0.01	n.s.	n.s.	n.s.	n.s.

^a^ Values in the same row with different small letter are significantly different (*p* < 0.05) or show tendency for difference (0.1 < *p* < 0.05) following Duncan’s HSD, *post hoc* test; n. s. non-significant.

^b^ OM > IM.

In our trial, dietary mineral supplementation level had limited effects on fish performance, except between the first and the second handling-stress treatment, where we saw a significant interaction between mineral source and level (*p* < 0.008) with higher growth rate and lower FCR at higher OM supplementation levels and no such effect among the IM treatments. Similarly, in a previous study from our research group we saw improved FCR in salmon parr, before, during and after smoltification fed diets with high supplementation levels of organic trace minerals (Kousoulaki et al*.* 2021). There were no other significant effects or tendencies in FCR among the different dietary treatments and for the different trial variables. Published studies have shown differential fish performance at variable dietary mineral levels or source (Nguyen et al., 2019; Pierri et al*.* 2021). In the study of Prabhu et al*.*(2019a), increasing dietary micronutrient nutrient supplementation, including inorganic minerals (Cu: 8.31–30 mg/kg, Mn 37.1–95.8 mg/kg, Zn: 62–243 mg/kg, Se:0.42–1.39 mg/kg), enhanced salmon parr growth but there was no such correlation with post-smolt performance. Generally, there is evidence of positive effects by dietary organic minerals in the performance and feed utilisation of both fish and crustaceans (Apines et al., 2003; Katya et al., 2017; Prabhu et al*.* 2019a; Prabhu et al*.* 2019b; El-Sayed et al., 2023), but different inclusion level results are hard to compare due to differences among species, animal sizes and thus requirements, and diet formulation.

### 3.2 Nutrient apparent digestibility coefficient (ADC)

**FIGURE 2:**
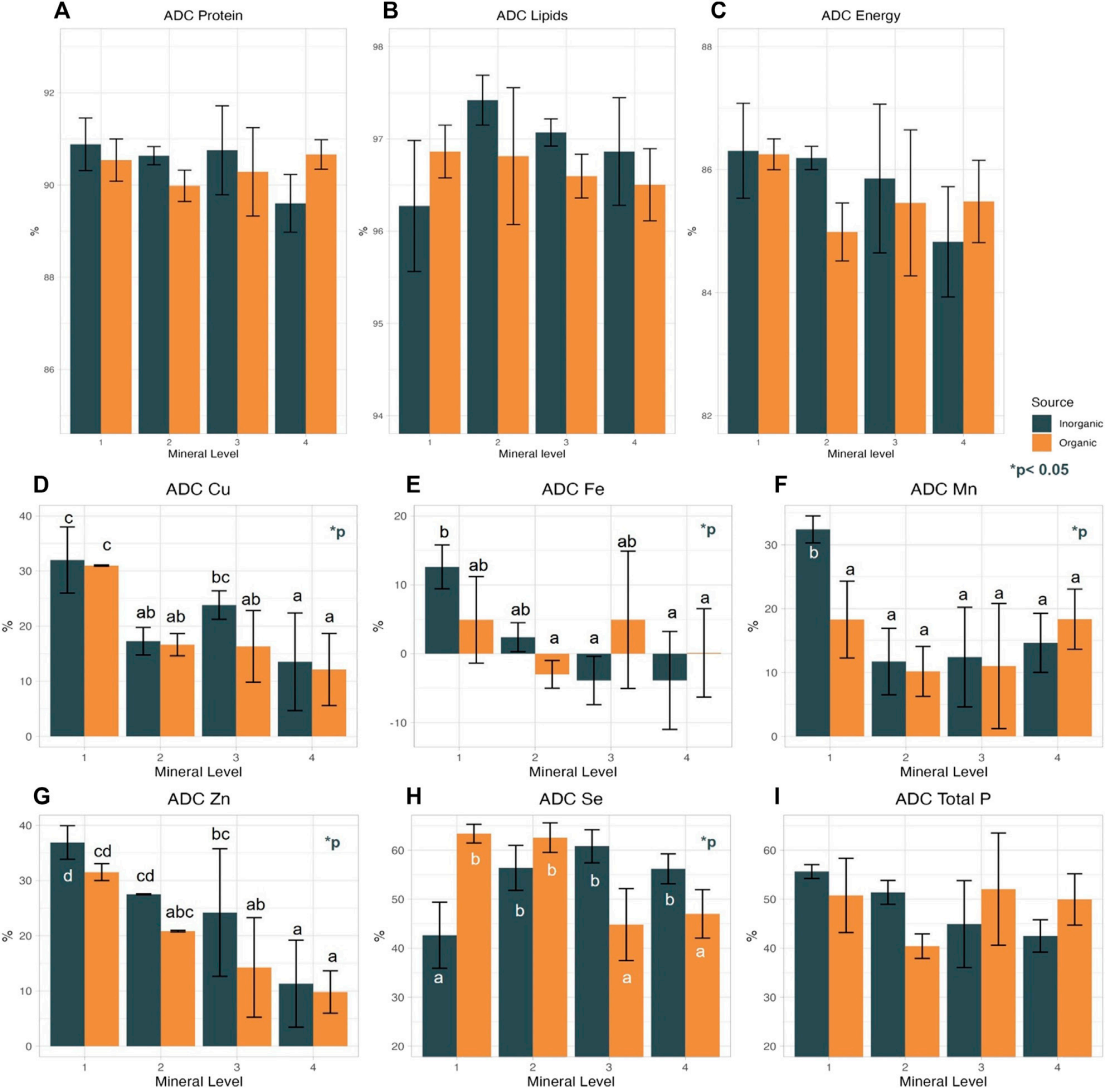
Apparent digestibility coefficient (ADC) of dietary protein **(A)**, lipid **(B)**, and energy **(C)**, as well as ADC of P **(I)** and 5 tested minerals Cu **(D)**, Fe **(E)**, Mn **(F)**, Zn **(G)**, Se **(H)** of Atlantic salmon fed diets of different trace mineral source (inorganic, organic) and levels, Values in the same graph with different small letter are significantly different (*p* < 0.05) following Duncan *post hoc* test.

In most cases, ADC of the test trace minerals was higher at the lowest supplementation level, apart from Se in the IM treatments that showed the opposite effect (Supplementary Table S5). In previous studies focusing on mineral digestibility similar results have been found; specifically, in the study of Prabhu et al. (2019a) numerically higher apparent availability (AA) of Cu, Zn, Mn was observed at lower supplementation levels, while AA of Se had a fluctuating pattern with a trend to increase with increasing inclusion levels. In a later study of Prahbu’s research group in which they focused on Se requirement comparing different sources and doses, Se AA was significantly lower in diets with higher supplementation levels of inorganic Se, while increased in diets supplemented organic Se (Prahbu et al., 2020). The term AA is used to replace ADC in reference to minerals to highlight that the values obtained are the unabsorbed minerals from the diet plus the digestive secretions (NRC, 2011). Both AA and ADC are calculated using the same equation. In our study, mineral source had nearly no effect on nutrient ADC, except in the case of Zn, which was approx. 6% higher (significantly; *p* = 0.033) in the IM groups. Our findings are in contrast with the findings in the studies of Silva et al. (2019) and Yarahmadi et al. (2022), in which Zn AA, did not correlate with Zn source (organic or inorganic). In Yarahmadi et al. (2022), the highest AA of Zn was achieved at 130 mg/kg of dietary Zn supplementation, an inclusion level that in our study resulted in lower Zn ADC compared to even lower dietery Zn levels. Nevertheless, we observed interactions between mineral source and supplementation level on the ADC of P, Fe (tendency, *p* = 0.055 and *p* = 0.075, respectively), and Se (significance, *p* < 0.001). In the case of dietary P, we saw interaction effects between trace mineral source and trace mineral dietary level, though P was not part of the experimental design. The ADC of P (Figure 2I), though present in similar amounts in the diets, correlated negatively with increasing trace mineral supplementation level in the IM groups, whereas in the OM groups P ADC was constant despite the higher feeding levels in the OM groups, which growing more may have resulted in higher P retention (not calculated) and possibly lower losses in the environment. Fossil P is a non-renewable resource, and concerns about the near future have arisen, as it is believed that P will be a limited resource for food production (Scholz et al., 2014). Retention of dietary P in salmonid aquaculture in Norway is approximately 30% resulting in the release of 11,000 tons of P in the environment yearly (Ytrestøyl, Aas and Aasgård, 2015). To mitigate P losses and potential negative environmental impacts of aquaculture, and avoid depletion of natural mineral resources, best management practices should be implemented to reduce P use in aquafeeds, among other potentially that of using organic trace mineral supplements in the feeds.

The ADC of dietary Fe decreased with increasing supplementation level in the IM groups, whereas there was no such trend in the OM groups, though Fe concentration in the faeces increased significantly with increasing dietary Fe supplementation in both IM and OM groups (Figure 2E; Supplementary Table S5). ADC of Se increased with increasing supplementation in the IM groups, and it decreased in the OM groups (Figure 2H). ADC of dietary Se significantly correlated with treatment (*p* < 0.00) and there was an interaction of mineral source and level (*p* < 0.05). Specifically, IM1, OM2-3-4 diets had the lowest Se digestibility, while IM2-3-4 and OM1-2 the highest. ADC of Se in our study was lower (42.5%–63.5) compared to the results in Silva et al. (2019) (58%–74%), and to the results in Prabhu et al*.* (2018), in rainbow trout (79.9%–81.9%) fed plant-based diets supplemented with inorganic Se (selenite). The diets in our study contained Se levels above EU max limit (total Se in complete fish feeds with 12% moisture is 0.5 mg/kg, (EU) No 121/2014), but previous studies on Atlantic salmon have shown that the optimal dietary Se level in low fish meal diets are above this EU limit (Prabhu et al., 2019a; Prabhu et al., 2020). Our results suggest that 0.7–0.8 ppm more inorganic Se is required than the legal limit to achieve better digestibility while 0.64 ppm more organic Se. Regarding the ADC of dietary macronutrients and energy, no effects of supplemented trace mineral level or origin was found (Figures 2A–C).

### 3.3 Blood chemistry

**FIGURE 3:**
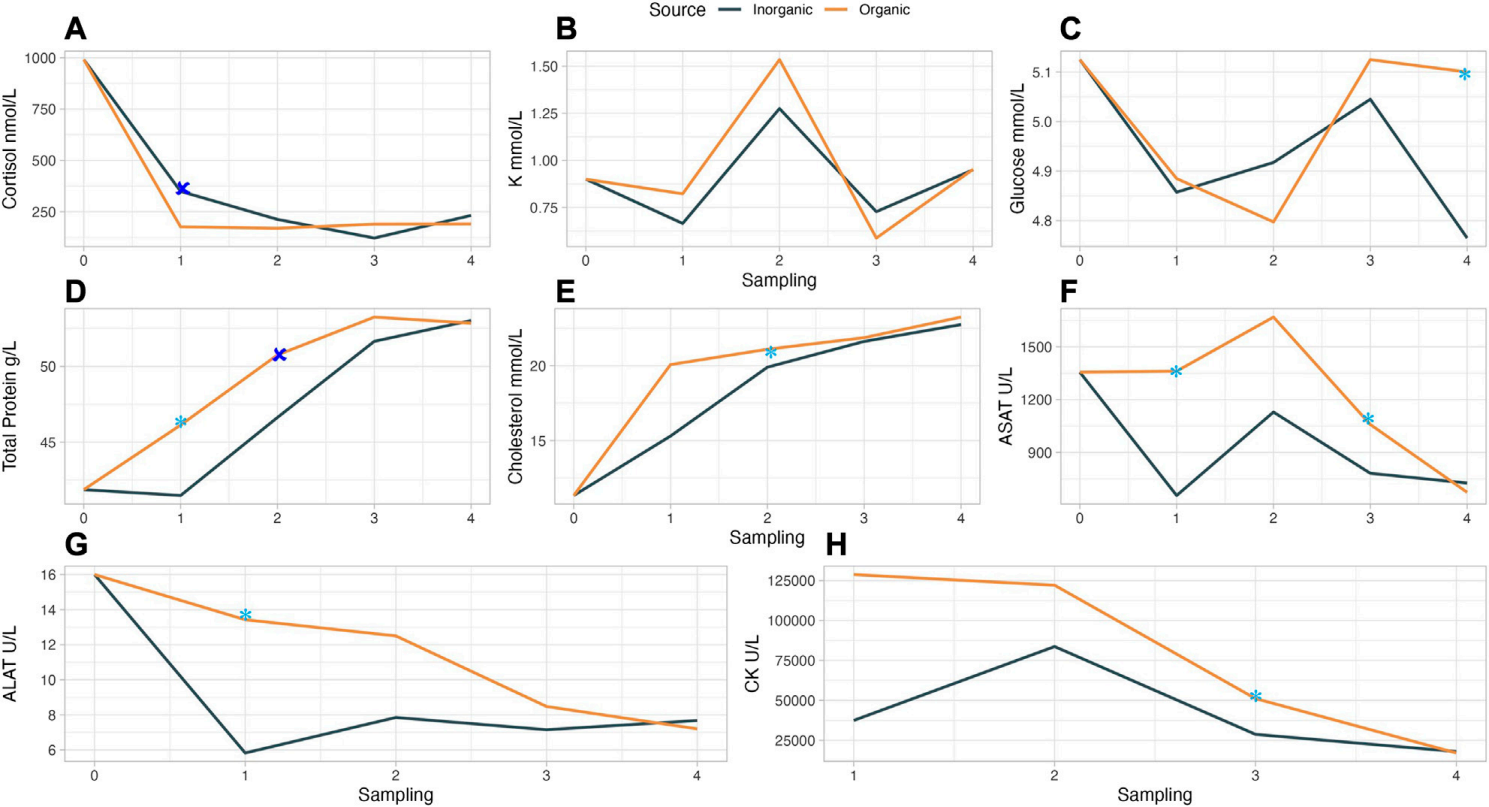
Blood serum metabolites in handling stressed (HS) Atlantic salmon fed diets with trace minerals source (IM or OM) at different time points (0: start, 1: HS1, 2: HS2, 3: HS3, 4: final sampling).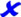 indicates significantly (*p* < 0.05) higher (↑) value for the specific sampling and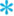 indicates tendency following Duncan *post hoc* test. **(A)** ↑ IM Cortisol in 1^st^sampling (*p* = 0.023). **(B)** K no significant differences between IM and OM. **(C)** ↑ OM Glucose in final sampling (*p* = 0.078). **(D)** ↑ OM Total Protein in HSI (*P* = 0.091) and HS2 (*P* = 0.018). **(E)** ↑ OM cholesterol in HS2 (*P* = 0.057). **(F)** ↑ OM ASAT in HS1 (*P* = 0.074) and HS3 (*P* = 0.076). **(G)** ↑ OM ALAT (*P* = 0.055), **(H)** ↑ OM CK in HS3 (*P* = 0.087).

Among other properties, minerals such as Se, Zn, Cu, and Mn have an antioxidant role (Zhang et al., 2016) that can be fundamental in fish’s protection against oxidative and other types of stress. Stress markers as well as indicators for the general condition of the farmed fish can be evaluated by blood chemistry. In our study, there were only few statistically significant differences or tendencies in the measured serum parameters, possibly due to a large variation among parallel tanks (Supplementary Table S6). Specifically, blood serum cortisol was higher at trial start and remained at lower levels and similar among the dietary treatments during the handling stress treatments (handling stress 1–4) and the final sampling. Cortisol levels were significantly higher (*p* = 0.023) in IM as compared to OM fish after HS1 (Figure 3A). Serum cortisol levels in our experiment were similar (Sandodden et al*.* 2001) or higher than in other studies (Graff et al., 2002; Djordjevic et al., 2021) where fish were submitted to handling stress and related treatments, for instance, transport. Similarly, serum glucose levels in our study (4.36–5.38 mmol/L) were similar as in Sandodden et al*.* (2001) and fluctuated in both mineral source groups, but at different sampling points. Glucose levels tended to be higher in OM groups (Figure 3C) at the final sampling (*p* = 0.078) which can be an indication that the OM fed fish could more efficiently mobilise higher energy levels to repair damages due to handling. When fish experience stress, cortisol is released from their adrenal glands, stimulating the synthesis of glucose from non-carbohydrate sources (gluconeogenesis). This elevated glucose release provides immediate energy to cope with stress, supporting physiological and behavioral responses through mobilization of energy reserves and increased metabolism (Schreck and Tort, 2016). Cortisol can have also an impact on electrolyte balance by influencing the movement of potassium (K) ions across cell membranes, potentially leading to changes in blood potassium (K+) levels (Mommsen et al., 1999). The specific mechanisms are complex and vary depending on species and stressor. In our study, K+ values increased in HS3 for both OM and IM. Elevated stress levels can also lead to an increase in total serum protein due to factors such as increased production of acute-phase proteins or changes in protein turnover (Schreck and Tort, 2016). We observed a gradual increase in total serum protein until HS3 which then stabilised for both groups; OM treatments though, led to a higher elevation of total serum protein levels after HS1 (*p* = 0.091) and HS2 (*p* = 0.018) as compared to IM. Cholesterol levels increased until the end of the experiment, similarly for both OM and IM (Figure 3E) and were in similar levels with the recently published results of our research group (Kousoulaki et al., 2022). As mentioned before, Cu is essential for lipid metabolism and the synthesis of lipoproteins, which are responsible for transporting cholesterol. Adequate Cu levels support proper lipid metabolism and cholesterol transport in fish (Chen et al., 2015). Fe, on the other hand, plays a role in lipid peroxidation, a process that can indirectly impact cholesterol metabolism. Imbalances in iron levels may affect lipid peroxidation, potentially influencing cholesterol levels in fish (Burry and Grossel, 2003).

Aspartate and alanine transaminases (ASAT and ALAT) are liver metabolites released when oxidative damage occurs in liver cells (Sandnes et al., 1988). Nevertheless, elevated serum ASAT and ALAT levels in Atlantic salmon have correlated with increased fish performance (Kousoulaki et al., 2022), whereas in Nile tilapia’s liver (Gaye-Siessegger et al., 2007) they have been associated with amino acid metabolism. In our study, a fluctuation of ASAT (Figure 3F) levels was observed for both mineral groups and highest levels after HS2; OM tended to have higher ASAT levels after HS1 (*p* = 0.074) and HS3 (*p* = 0.076). ALAT levels (Figure 3G) tended to be higher in OM group after HS1(*p* = 0.055). In the study of Kousoulaki et al. (2022) a positive correlation between TGC, ASAT, ALAT, and creatinine kinase (CK) was observed. Our results indicate a similar positive correlation between TGC and the liver and the respective muscle metabolites in the OM group. In our study, CK (Figure 3H) declined gradually for both OM and IM groups reaching the same final level but was numerically higher in the OM group in the two first HS and trended to be higher in HS3 (*p* = 0.087). Generally, there are indications of better stress tolerance and endocrine responses when fish are fed organic minerals (Herrera et al., 2019; Meiler et al. 2021) but the underling mechanisms need further investigation.

### 3.4 Tissue mineralisation and retention efficiency

We found significant treatment effects in all tested mineral retention efficiency (RE) values (Supplementary Table S7). Except for Cu RE, trace mineral RE was numerically higher in the OM treatments, significantly only for Se. Except for Se, trace mineral RE decreased significantly with increasing dietary supplementation level both in the IM and OM groups. More specifically, Cu RE was highest (∼23.78%) at the 1st supplementation level for both mineral sources (OM1–IM1), and lowest (∼9.74%) at the 3rd and 4th supplementation level again for both sources. Increasing levels of Cu in the diet of Atlantic salmon smolts (comparable to the levels in our study) resulted in decreasing Cu RE (Prahbu et al., 2019b). In the same study Prabhu et al. (2019b) found overall lower (20%) Cu RE compared to our study. Fe RE was numerically higher in OM1 (7.8%) diet and lower in IM4 (3.5%), and supplementation level and the interaction of mineral level and source influenced Fe RE. Highest Mn RE (∼5.19%) was observed in the 1st supplementation level for both mineral sources; once again in our study we achieved higher Mn RE compared to the results (4.3%) of the study of Prabhu et al. (2019a). The same was observed also for Zn RE with the study by Prabhu (2019a) showing highest Zn RE of 10.1%, at 140 mg/kg dietary Zn using and inorganic supplemental source (Zn oxide), while in the present study we achieved overall Zn RE above 18% and highest (29.1%) at IM2 and OM1 diets (107 and 87 mg/kg dietary Zn in the diets, respectively). As mentioned before, Se RE was the only RE response affected by mineral source, with OM (highest: 42.9% OM1) reaching higher values compared to the IM (lowest 13.2% IM2) groups, while the interaction of mineral source and level significantly (*p* = 0.041) affected the Se RE. Organic minerals (Cu, Fe, Mn, Zn) supplemented in different levels in all plant feeds for Nile tilapia had similar mineral RE to our results; namely, Mn was the least retained while Se showed the highest RE. The RE of Fe, Cu, Mn, and Zn decreased with the increasing dietary mineral supplementation levels (Nguyen et al., 2019). Better Se RE, was also reported by Prabhu et al. (2020) when salmon fed organic dietary Se (selenomethionine) compared to inorganic (sodium selenate); in the same study they emphasised that dietary Se required to achieve body homeostasis exceeds existing EU max limits. In another study, in salmon pre-smolts, Vera et al. (2020) used increasing levels of inorganic minerals (sodium selenite, ferrous sulphate monohydrate, manganous oxide, cupric sulphate pentahydrate and zinc oxide), with comparable Se dietary levels as compared to our study, reaching however remarkably lower whole-body Se RE values (12.2% and 17.9%). In line with the existing body of scientific literature, our findings align with the consensus that dietary supplementation of organic Se in fish diets leads to superior Se RE compared to inorganic Se sources.

**FIGURE 4:**
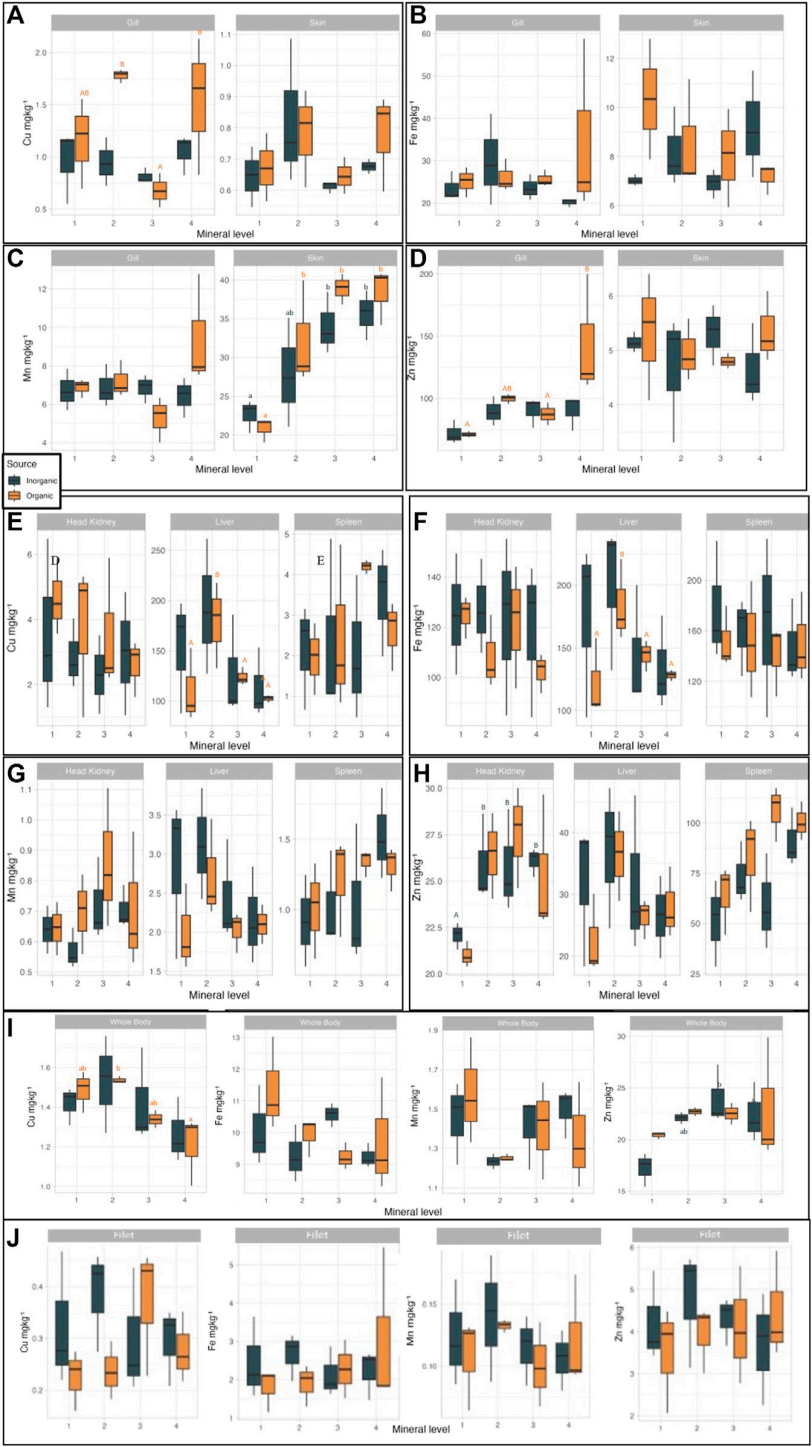
Tissue mineralisation in Atlantic salmon when fed diets with added trace minerals in either inorganic (IM) or organic (OM) form at variable levels (values are means# standard variation; *n* = 3 tanks). Values in the same graph with different small letter are significantly different (*p* < 0.05), whereas those with different capital letter show tendency for difference (*p* < 0.1) following Duncan *post hoc* test. Letter’s color follow the same pattern (orange for OM and dark blue for IM). **(A)** Cu concentration in gills and skin; correlation between mineral level and gill Cu, in OM (*p* = 0.069). **(B)** Fe concentration in gill and skin. **(C)** Mn concentration in gill and skin; correlation between mineral level and skin Mn, in IM (*p* = 0.026) and OM (*p* = 0.011). **(D)** Zn concentration in gill and skin; correlation between mineral level and gill Zn, in OM (*p* = 0.074). **(E)** Cu concentration in head kidney, liver, and spleen, correlation between mineral level and liver Cu in OM (*p* = 0.060). **(F)** Fe concentration in head kidney, liver, and spleen; correlation between mineral level and liver Fe in OM (*p* = 0.0584). **(G)** Mn concentration in head kidney, liver, and spleen. **(H)** Zn concentration in head kidney, liver, and spleen. **(I)** Whole body mineralisation (Cu, Fe, Mn, Zn); correlation between mineral level and whole body Cu in OM (*p* = 0.030), correlation between mineral level and whole body Zn in IM (*p* = 0.028). **(J)** Filet mineralisation (Cu, Fe, Mn, Zn), no correlation between mineral level filet mineralisation (in neither source).

In order to investigate how increasing dietary mineral levels of different source may affect mineral homeostasis in salmon, we analysed mineral levels in different tissues at trial start and end and saw that both factors induced significant effects (Supplementary Table S7). Copper is present in metalloenzymes and other metalloproteins like cytochrome c oxidase (COX), a protein which is encoded in mitochondrial genome (Broadley. 2012) and catalyzes the reduction of molecular oxygen to water. Gill filaments have numerous mitochondria-rich (MR) cells proliferating to increase the ion regulatory capacity of the organ (Lin and Sung, 2003). Branchial expression of COX-IV gene and COX enzyme activity in the mitochondrial electron transport chain is the outcome MR cells activity in fish gills (Zikos et al., 2014). In our study, Cu was positively corelated with OM source in the gills (*p* < 0.021) with a maximum concentration of 1.8 mg/kg (in OM2). This correlation can be related to the bioavailability of the dietary organic Cu leading to enhanced Cu metabolism and incorporation into ceruloplasmin in the liver and transport to extrahepatic organs like muscle (Lall, 2022) and promoting accumulation also in other organs, like gills. Cu concentration in the gills was significantly (*p* = 0.022) affected by mineral supplementation levels; supplementation level 3 induced the lowest Cu concentration for both mineral sources and the highest on levels 2 and 4 in the OM groups. In the liver, supplementation level 2 led to the highest Cu concentration, mineral source independent, while level 4 the lowest (*p* = 0.037). A correlation between concentration of Cu in the liver and supplementation level was observed withing the OM groups (*p* = 0.06) (Figure 4E). Whole body Cu (Figure 4I) levels, as also seen in the liver, was significantly higher at supplementation level 2 while at level 4 were the lowest for both mineral source groups. In the study of Taylor et al. (2019) where 3 diets with comparable dietary Cu (inorganic) levels with the first 3 levels in our study were tested, salmon parr whole body Cu was not affected by the increasing micronutrient supplementation levels. Overall, in our study Cu whole body mineralisation was lower than in other published studies-that report whole body Cu values above 2 mg/kg (Prabhu et al., 2019a; Vera et al., 2020).

Iron was not significantly correlated with treatment or the interaction of mineral source and inclusion level in any tissue; but as with Cu, there was a trend (*p* = 0.084) for higher Fe concentration in the liver in the OM treatments (Figure 4F). In the study of Rigos et al. (2010), a trend of better bioavailability of organic Fe in contrast to inorganic was demonstrated is gilthead sea bream (*Sparus aurata*) fed organic and inorganic Fe in increasing dietary levels. In the same study, liver Fe levels were numerically higher in sea bream fed organic Fe.

Manganese levels were not affected significantly by treatment in any studied tissue but in the gills, where we saw was a trend (*p* = 0.071) of mineral source and level interaction. This aligns with Prabhu et al. (2019b), that suggest that the efficacy of dietary Mn sources should be evaluated in conjunction with the level of inclusion. In the skin, Mn concentration was correlated with mineral level but independently for each mineral source (Figure 4C). Specifically, fish fed IM had significantly (*p* = 0.026) lower Mn in the skin at IM1level (22.7 mg/kg) and highest in IM3 and 4. OM1 fed fish had also significantly (*p* = 0.011) lower Mn in the skin, and the highest already from OM2 (∼35 mg/kg), with no further increase at OM3 and OM4 (Supplementary Table S8). The results on whole body Mn (Figure 4I) in our study agree with the findings of Prabhu et al. (2019b) even if higher Mn levels were supplemented in the diets of our study [50–100 mg/kg vs*.* 5–65 mg/kg in Prabhu et al. (2019b)]. Probably because of that we also saw in our study higher liver Mn concentrations as compared to the study of Prabhu et al. (2019b).

**FIGURE 5:**
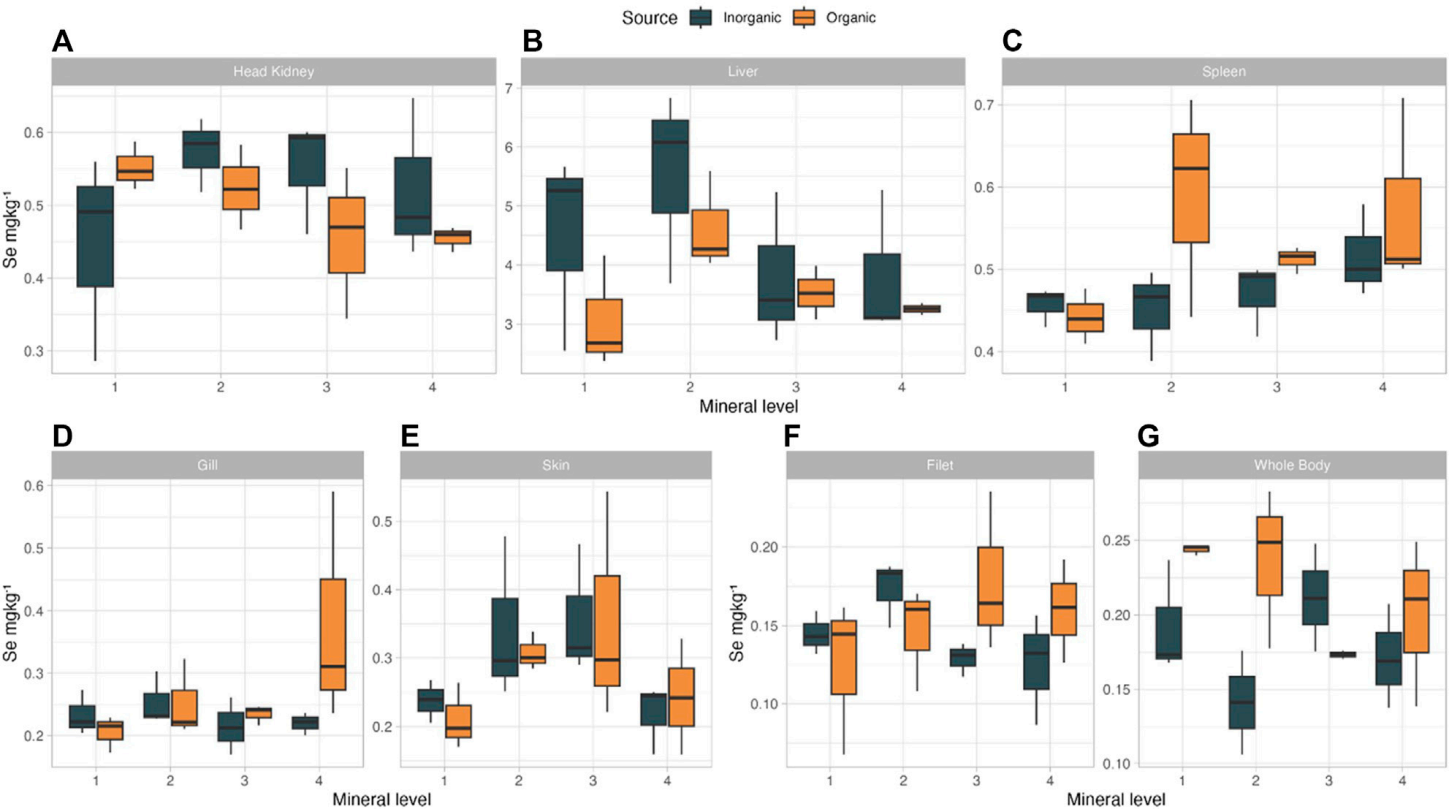
Se concentration in tissue [**(A)** Head Kidney, **(B)** Liver, **(C)** Spleen, **(D)** Gill, **(E)** Skin, **(F)** Filet, **(G)** Whole Body] Atlantic salmon when fed diets with added trace minerals in either inorganic (IM) or organic (OM) form at variable levels.

Selenium is an essential component of selenium containing proteins (selenoproteins) involved in free radical metabolism and immune responses (Labunskyy et al*.* 2014). Whole body and tissue Se levels were affected by the different treatments and were numerically higher in the OM groups in whole body and spleen (Figure 5). Mineral source and the interaction of mineral source and inclusion level significantly (*p* < 0.044) affected Se in the whole body of salmon, with the highest concentration in OM1 (0.247 mg/kg) and the lowest in IM2 (0.143 mg/kg). In the spleen, we saw a trend (*p* = 0.075) for higher Se concentration in the OM as compared to IM treatments, with highest levels in OM2 (0.59 mg/kg). Skin Se levels tended to increase with increasing supplementation levels (*p* = 0.085) until level 3 in both OM and IM groups. The ADC and RE results combined with the observed levels in the whole body and target tissues, such as the spleen, at lower supplementation levels, indicate that organic Se is more bioavailable than the inorganic source we used. Better organic Se bioavailability has been reported on farmed fish and terrestrial animals indicating a clear trend on more efficient retention of organic Se; specifically filet of Nile tilapia (Nguyen et al., 2019), African catfish (*Clarias gariepinus*) whole body (Darmawangsa et al., 2021), fillet of salmon (Betancor et al*.* 2016; Sele et al*.* 2018; Silva et al*.* 2019), breast muscle of hens and eggs (Pan et al., 2007), milk of dairy cows (Gong et al., 2014) and muscle of pigs (Jiang et al., 2017) had improved/higher Se deposition when organic Se replaced inorganic Se in the animal’s diets.

Whole body Zn concentration in our study increased significantly from IM1 to IM3 (*p* < 0.044). In a recent study, Yarahmadi et al. (2022) found that whole body Zn levels increase with the increasing levels of dietary (inorganic) Zn; in a Zn inclusion level of 182 mg/kg they measured 36.5 mg/kg Zn in the whole body while in their lowest Zn supplementation level (40 mg/kg) they reported 24.5 mg/kg whole body Zn. In our study, the highest whole body Zn concentration (24 mg/kg) was measured at fish fed IM3 diet (dietary Zn level 133 mg/kg). In the spleen, Zn levels increased numerically with increasing supplementation level up to OM3 and IM4 and were significantly higher in the OM as compared to the IM groups (*p* < 0.018). In the kidney Zn levels increased until supplementation level 2 for both IM and OM groups (*p* < 0.05). In their study, Maage et al. (2001) investigated the effect of Zn source (organic vs*.* inorganic) in 2 inclusion levels (low: 130 mg/kg, and high: 230 mg/kg) when fed to Atlantic salmon for 24 weeks. They reported significantly higher whole body and vertebrae Zn concentration in salmon fed high Zn level diets (source independent). In the skin, both in OM and IM, Zn levels varied irregularly with respect to the supplementation levels (*p* < 0.001) but increasing mineral levels increased skin Zn concentration. Zn plays an important role in wound healing both in humans, as it stimulates epithelialization of wounds (Lansdown et al., 2007), as in fish by improving skin health and wound healing ability (Jensen et al., 2015). In their study Jensen et al. (2015) fed 60 mg more Zn than the current EU max allowed Zn level (180 mg/kg) to salmon smolts which resulted in improved skin health and activation of rapid wound healing mechanisms compared to the controls. Following the pattern of increasing tissue Zn level with increasing Zn levels in the feed, gill Zn levels in our study increased significantly from the first to the fourth supplementation level for both OM and IM groups (*p* < 0.009), more markedly in OM4. In salmonids (and possibly in all teleosts) it is suggested that Zn excretion takes place primarily through ion exchange in the gills (Nakatani, 1966; Hardy et al., 1987). This suggestion could enlighten our findings and the increasing Zn levels in the gills with the increasing supplementation levels could be due to activation of excretory mechanisms by the fish to maintain mineral homeostasis, more markedly in the OM4 groups indicating higher availability of the used organic Zn supplement. The European Commission (EC) had already reduced the maximum allowed limit for Zn from 200 mg/kg to 180 mg/kg (EC, 2016) and the European Food Safety Authority (EFSA) had proposed in the past (EFSA Panel on Additives and Products or Substances used in Animal Feed, 2014) a further reduction of Zn to 150 mg/kg which could militate fish requirements. This study in accordance with numerous others (Maage, Julshamn and Berge (2001; Jensen et al., 2015; Prabhu et al., 2019a; Vera et al., 2020; Yarahmadi et al., 2022) demonstrates a clear requirement for higher dietary Zn levels in the diets of Atlantic salmon. The environmental concerns that thrust EC to stricter legislations could be eased with better Zn retention, which is feasible as shown in this study and discussed before.

Fish in the OM groups consumed more feed than in IM and were also found to have higher levels of the inert mineral marker yttrium, added in all diets at equal levels, in the gills as compared to the IM groups (*p* < 0.023). As for Zn, it may be that excretion of superfluous Y levels were concentrated in the gills to further be excreted.

Tissue macromineral [potassium (K), Magnesium (Mg), Calcium (Ca) and Natrium (Na)] levels were also measured, though supplemented at equal amounts in the different trial diets (not analysed). Tissue Potassium levels did not correlate with mineral source, level, or their in between interaction in any tissue except for kidney. Fish kidney has a dual role, it filtrates and excretes wastes from the blood, and is also responsible for selective nutrient reabsorption and erythropoiesis (Iqbal et al., 2004). In our study, kidney K levels tended to be higher in the IM (*p* = 0.055) as compared to the OM groups. In fish, K excretion takes place mainly in the kidney (Basalo, 2022) thus it could be possible that IM groups had increased K excretion. Magnesium and calcium mineralisation was not affected by the experimental treatments, except from the gills where Mg and Ca concentrations tended to decrease at IM4 and increase in OM4 (*p* = 0.09 and *p* = 0.065 for Mg and Ca, respectively). If the increasing supplementation levels of the tested minerals and their different sources affected the upregulation or excretion of some microelements is difficult to conclude; further studies need to be conducted in order to understand the underlying mechanisms of micro and microelement interaction and how this could affected by micromineral different level and source.

### 3.5 Whole body lipid and fatty acid profile

Lipid metabolism and active lipogenesis have been correlated with Se supplementation in farmed animal diets like salmon (Kousoulaki et al., 2016), pigs (Zhao et al., 2016) and broiler chickens (Marta del Puerto et al., 2017). Fe and Zn, among other components, function as co-factors or coenzyme precursors for essential long chain n-3 polyunsaturated fatty acid (LC n−3 PUFA) biosynthesis (Lewis et al*.* 2013) and play also role in the bioconversion of alpha-linolenic acid (ALA) to eicosapentaenoic acid (EPA), and docosahexaenoic acid (DHA) in salmonids (Eder and Kirchgessner, 1994; Mahfouz et al., 1989; Stangl and Kirchgessner, 1998; Zhou et al*.* 2011). They also play an essential role in lipid metabolism as co-factors and co-enzymes in enzymes involved in peroxisomal β-oxidation (Osumi and Hashimoto, 1979; Poirier et al., 2006). In our study, whole body total lipids were not affected by mineral source or level. Nevertheless, several, rather small but significant differences were seen in the whole-body fatty acid profile of fish in the different experimental groups (Table 4). The saturated fatty acid 14:0, most analysed monounsaturated fatty acids [e.g., myristic acid (16:1 n-7) (*p* < 0.029), nervonic acid (24:1 n-9) (*p* < 0.05), eicosaenoic acid 20:1 (n-9)+(n-7) (*p* < 0.011), erucic acid 22:1 (n-11) + (n-9) + (n-7) (*p* < 0.003)], EPA (20:5 n-3) (*p* = 0.003), DPA (22:5 n-3) (*p* = 0.049) and DHA (22:6 n-3) (*p* = 0.026), were significantly higher in the whole body lipids of the OM as compared to the IM groups, and no differences were seen in the n-6 fatty acids. As a consequence, the ratio omega-6/omega-3 was higher in the IM as compared to the OM treatments (*p* < 0.000) with significant interaction between mineral source and levels, increasing in with increasing supplementation levels of inorganic and decreasing with increasing supplementation level of organic minerals. As mentioned before our research group has found increased EPA and DHA retention is salmon fed organic minerals (Zn, Cu, Mn, and Fe) (Kousoulaki et al., 2016). Moreover, the study of Tseng et al. (2021) in seabream further validates these findings as whole-body EPA and DHA was significantly higher in seabream groups fed organic Se compared to the inorganic control.

**TABLE 4:** Whole body (WB) (round/wet) total lipids (Bligh and Dyer extract) and fatty acid content (as % of whole body) of Atlantic salmon fed diets (8 treatments (T))with variable source (S) and levels (L) of trace minerals (values are means ± standard variation (a); *n* = 3 tanks)–(For complete fatty acid content see Supplementary Table S8).

	IM1	IM2	IM3	IM4	OM1	OM2	OM3	OM4	*p*-values			
									T	S	L	S x L
**WB B&D extract**	14.33 ± 0.33	14.78 ± 0.20	14.72 ± 0.55	14.18 ± 0.38	15.10 ± 0.27	14.63 ± 1.06	14.17 ± 0.13	14.29 ± 0.44	n.s.	n.s.	n.s.	n.s.
**14:0**	**0.39 ± 0.01**	**0.37 ± 0.01**	**0.38 ± 0.03**	**0.38 ± 0.01**	**0.39 ± 0.01**	**0.40 ± 0.03**	**0.40 ± 0.01**	**0.40 ± 0.04**	n.s.	**0.036** ^b^	n.s.	n.s.
**Saturated FA**	2.38 ± 0.04	2.36 ± 0.05	2.38 ± 0.19	2.36 ± 0.05	2.46 ± 0.05	2.48 ± 0.19	2.45 ± 0.06	2.39 ± 0.19	n.s.	0.159	n.s.	n.s.
**16:1 n-7**	**0.38 ± 0.00**	**0.38 ± 0.01**	**0.38 ± 0.03**	**0.38 ± 0.00**	**0.39 ± 0.01**	**0.41 ± 0.03**	**0.41 ± 0.02**	**0.40 ± 0.03**	n.s.	**0.029** ^b^	n.s.	n.s.
**20:1 (n-9) + (n-7)**	**0.86 ± 0.02**	**0.88 ± 0.02**	**0.86 ± 0.06**	**0.88 ± 0.03**	**0.91 ± 0.03**	**0.96 ± 0.06**	**0.92 ± 0.02**	**0.91 ± 0.07**	n.s.	**0.011** ^b^	n.s.	n.s.
**22:1(n-11) + (n-9) + (*n*-7)**	**0.79 ± 0.02**	**0.78 ± 0.02**	**0.76 ± 0.06**	**0.76 ± 0.01**	**0.79 ± 0.05**	**0.85 ± 0.04**	**0.84 ± 0.01**	**0.81 ± 0.05**	n.s.	**0.003** ^b^	n.s.	n.s.
**24:1 n-9**	**0.06 ± 0.01**	**0.06 ± 0.00**	**0.06 ± 0.00**	**0.06 ± 0.00**	**0.06 ± 0.00**	**0.07 ± 0.01**	**0.07 ± 0.01**	**0.06 ± 0.01**	n.s.	**0.050** ^b^	n.s.	n.s.
**Monounsaturated FA**	6.45 ± 0.05	6.63 ± 0.14	6.56 ± 0.49	6.59 ± 0.12	6.84 ± 0.18	6.80 ± 0.49	6.53 ± 0.11	6.41 ± 0.55	n.s.	n.s.	n.s.	n.s.
**PUFA (n-6) FA**	1.79 ± 0.04	1.88 ± 0.05	1.87 ± 0.14	1.86 ± 0.05	1.93 ± 0.05	1.87 ± 0.11	1.79 ± 0.04	1.77 ± 0.14	n.s.	n.s.	n.s.	n.s.
**20:5 n-3 (EPA)**	0.28 ± 0.01	0.27 ± 0.01	0.27 ± 0.02	0.26 ± 0.01	0.28 ± 0.00	0.29 ± 0.01	0.29 ± 0.01	0.28 ± 0.02	n.s.	**0.003** ^b^	n.s.	n.s.
**22:6 *n*-3 (DHA)**	0.69 ± 0.03	0.67 ± 0.02	0.66 ± 0.04	0.64 ± 0.00	0.68 ± 0.01	0.70 ± 0.03	0.69 ± 0.01	0.70 ± 0.05	n.s.	**0.026** ^b^	n.s.	n.s.
**PUFA (*n*-3) FA**	1.82 ± 0.05	1.80 ± 0.06	1.79 ± 0.12	1.75 ± 0.03	1.85 ± 0.05	1.87 ± 0.08	1.83 ± 0.03	1.82 ± 0.14	n.s.	n.s.	n.s.	n.s.
**omega-6/omega-3**	**0.98 ± 0.02** ^**a**^	**1.04 ± 0.01** ^**b**^	**1.04 ± 0.02** ^**b**^	**1.06 ± 0.02** ^**b**^	**1.04 ± 0.01** ^**b**^	**1.00 ± 0.02** ^**a**^	**0.98 ± 0.01** ^**a**^	**0.97 ± 0.01** ^**a**^	**0.000**	**0.000** ^**c**^	n.s.	**0.000**
**EPA + DHA**	**0.96 ± 0.03**	**0.94 ± 0.03**	**0.93 ± 0.06**	**0.90 ± 0.01**	**0.96 ± 0.01**	**0.99 ± 0.04**	**0.99 ± 0.01**	**0.98 ± 0.07**	n.s.	**0.011** ^b^	n.s.	n.s.

^a^ Values in the same row with different small letter are significantly different (*p* < 0.05), whereas those with different capital letter show tendency for difference (*p* < 0.1) following Duncan *post hoc* test.

^b^ OM > IM.

^c^ IM > OM.

### 3.6 Fish biometrics, welfare, and filet technical quality

**FIGURE 6:**
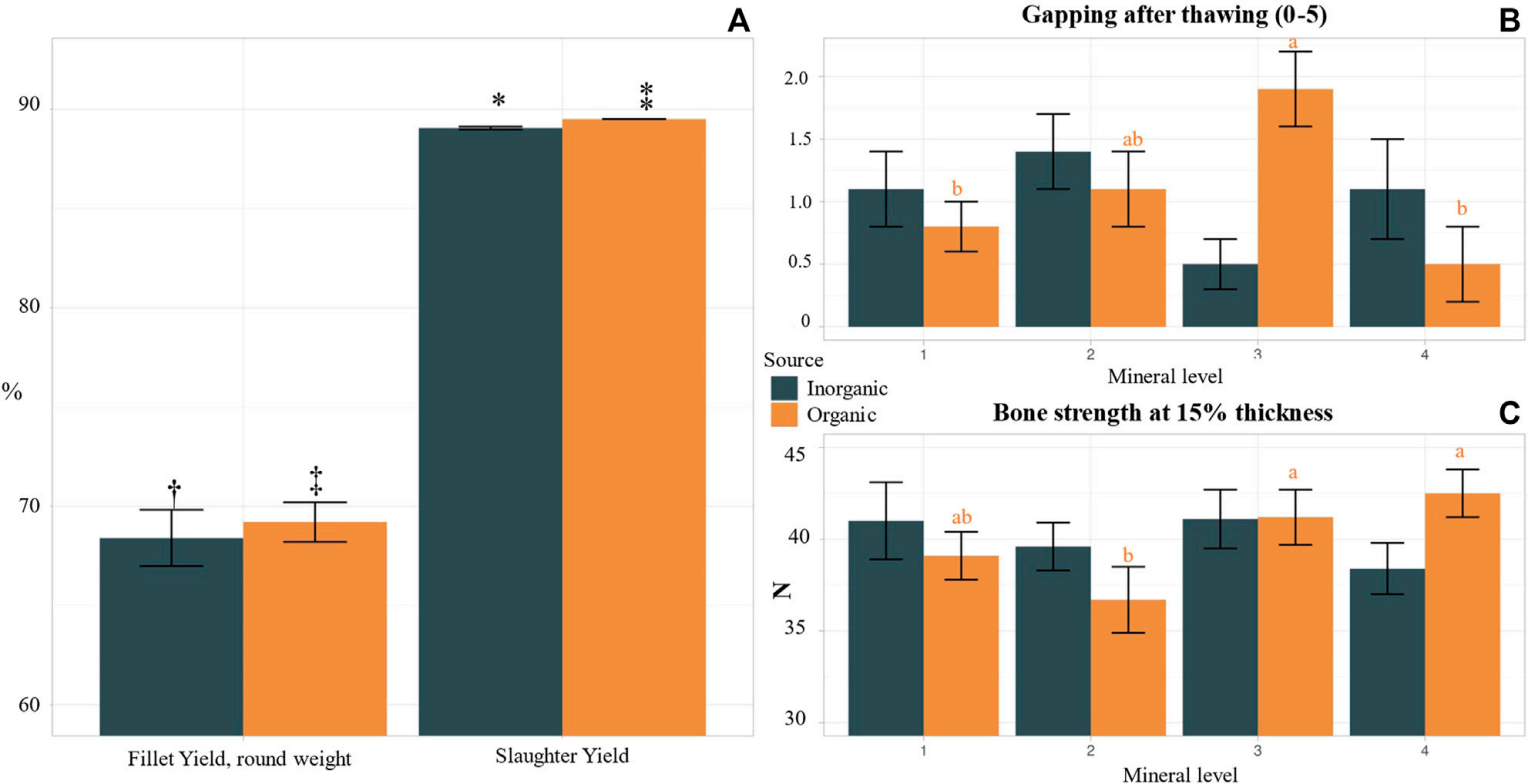
Biometrics, welfare, and technical fillet quality of Atlantic salmon post smolts, fed diets with variable source (OM, IM) and levels of trace minerals. Values in the same graph with different small letter or x symbol are significantly different (*p* < 0.05), whereas those with 1 symbol show tendency for difference (*p* < 0.1) following Duncan *post hoc* test. **(A)** Fillet and slaughter yield comparison between organic and inorganic groups, 1 fillet (0.084) and slaughter yield (*p* = 0.000) in OM. **(B)** Fillet gapping after thawing (from 0: no gapping, to 5 severe gapping), correlation between mineral level and gapping in OM (*p* < 0.01). **(C)** Bone strength (N), correlation between mineral level and bone strength in OM (*p* < 0.04).

We observed no significant effects of mineral supplementation level on slaughter yield, condition factor, HSI, or welfare score except for bone strength in OM fish (Supplementary Table S9), which agrees with the analysed higher Mg and Ca levels in the whole body of fish fed the OM added diets. Specifically, bone strength in OM3 and OM4 was higher as compared to OM2 but not significantly different from OM1 (Figure 6C) while bone strength was not correlated with increasing IM supplementation level. Zn is a key mineral in fish vertebrae and affects enzymes, like alkaline phosphatase (ALP), responsible for bone mineralisation (Prahbu et al., 2016). Better bone strength in 3^rd^ and 4^th^ supplementation level in the OM fish could also be attributed to better Zn mineralisation in the vertebrae but vertebrae samples were not collected/analysed in our experiment.

Fillet yield and quality parameters were influenced by the increasing supplementation levels for both tested mineral sources (Supplementary Table S9). Fillet color intensity, fat content, texture, firmness (hardness and elasticity) and gaping are of the most distinct parameters to evaluate quality and freshness of Atlantic salmon and are key elements in the products market value (Sigurgisladottir et al., 1997). In our study, significant decrease of fillet yield with increasing supplementation level of inorganic mineral premix was observed, more pronounced at IM4. Fillet of IM4 fish had also significantly higher liquid losses as compared to lower inorganic mineral supplementation levels, while fillet firmness peaked at IM3 and reduced again at IM4 level. Fillet liquid losses were lowest at OM2 and OM4 and highest at OM1 and OM3. Lowest gaping after thawing was observed at OM4 however not significantly different as compared to OM1, whereas the highest values were measured at OM3 followed by OM2 (Figure 6B). Hamre et al. (2020) have highlighted that mineral nutrition status of fish could affect fillet quality degrading conditions, for instance gaping, liquid losses, suboptimal pigmentation, and melanin spots. Our results can verify that mineral levels play a key role on salmon fillet’s technical quality. In the present study we compared also the two tested mineral sources regardless of mineral supplementation level and the results are presents in Supplementary Table S10. Fish in the OM treatments had similar round body weight, but they were longer and had significantly higher gutted body weight and slaughter yield (Figure 6A) and tendency for higher fillet yield (Figure 5A) as compared to fish in IM. Moreover, HSI was higher in IM as compared to OM. Similarly visceral fat was higher in IM as compared to OM. The differences in the effects of the two different mineral sources on fillet quality were related to muscle and skin color. There was a tendency for darker fillet color (more red) and brighter skin in the OM as compared to the IM treatments.

### 3.7 Skin histology

**FIGURE 7:**
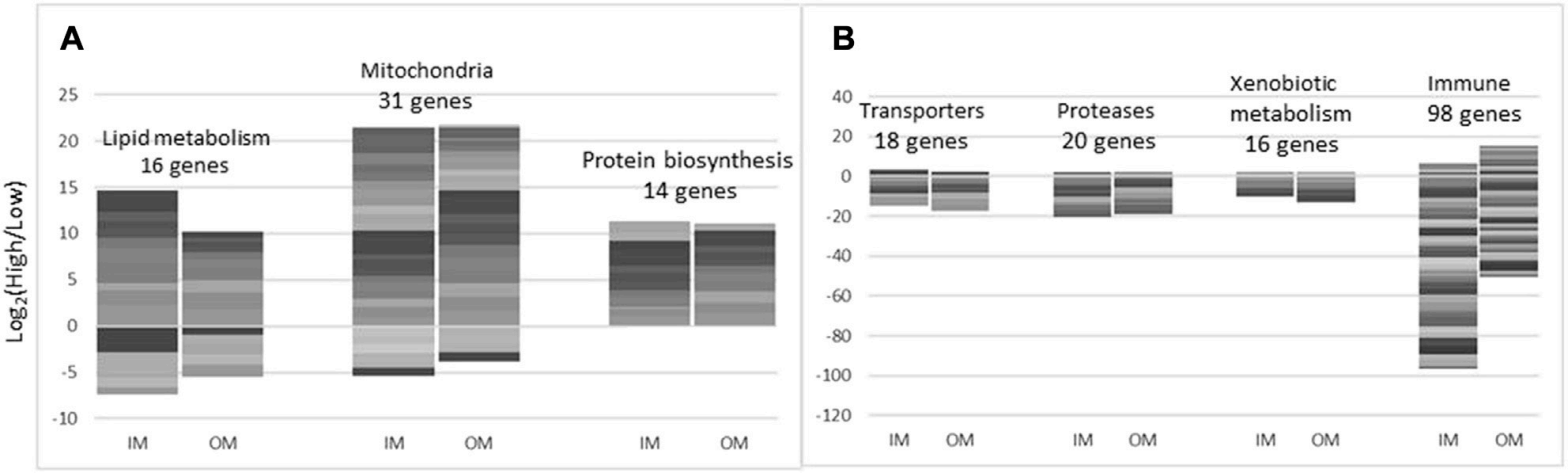
Gene functional groups **(A)** and individual genes **(B)** in IM and OM fish groups.

Mineral salts account for up to 1% of the mucus mass affecting its secretion and rheological properties (Lai et al., 2009; Raynal et al*.*2003), hence we were interested in the effect of the organic and inorganic minerals on fish skin and mucous production. A weak difference was detected for the mineral levels for mucous cell ratio to epidermal area (*p* = 0.047) (Figure 7A), meaning that the area of mucous cells to the area of epithelial tissue increase with increasing mineral level, but the *post hoc* test was not significant. Further the blue mucous cells were positioned closer to the apical border in OM compared to IM (*p* = 0.014) (Figure 7B), which may influence mucus secretion, while the *post hoc* test resulted in group differences between IM and OM at level 2 only.

Atlantic salmon skin with its viscous mucus layer is important for the protection of the fish surface from the surrounding environment. In our previous research fish fed a mineral diet (higher content of ash (mainly silicates), iron, iodine, and manganese, also resulted in higher number of skin mucous cells, relative to fish fed a commercial diet, and higher levels of iodine in the fish mucus (Sveen et al., 2021). Further, in rainbow trout (*Oncorhynchus mykiss*) is proposed that epithelial mucus secretion may play an important role in maintaining intestinal metal solubility (Glover and Hogstrand, 2003). Overall, the dietary levels of minerals (and source) seams of importance for skin mucus production, however more research is needed to fully understand the relationship between dietary minerals and fish mucus.

### 3.8 Midgut transcriptomics (microarray)

**FIGURE 8:**
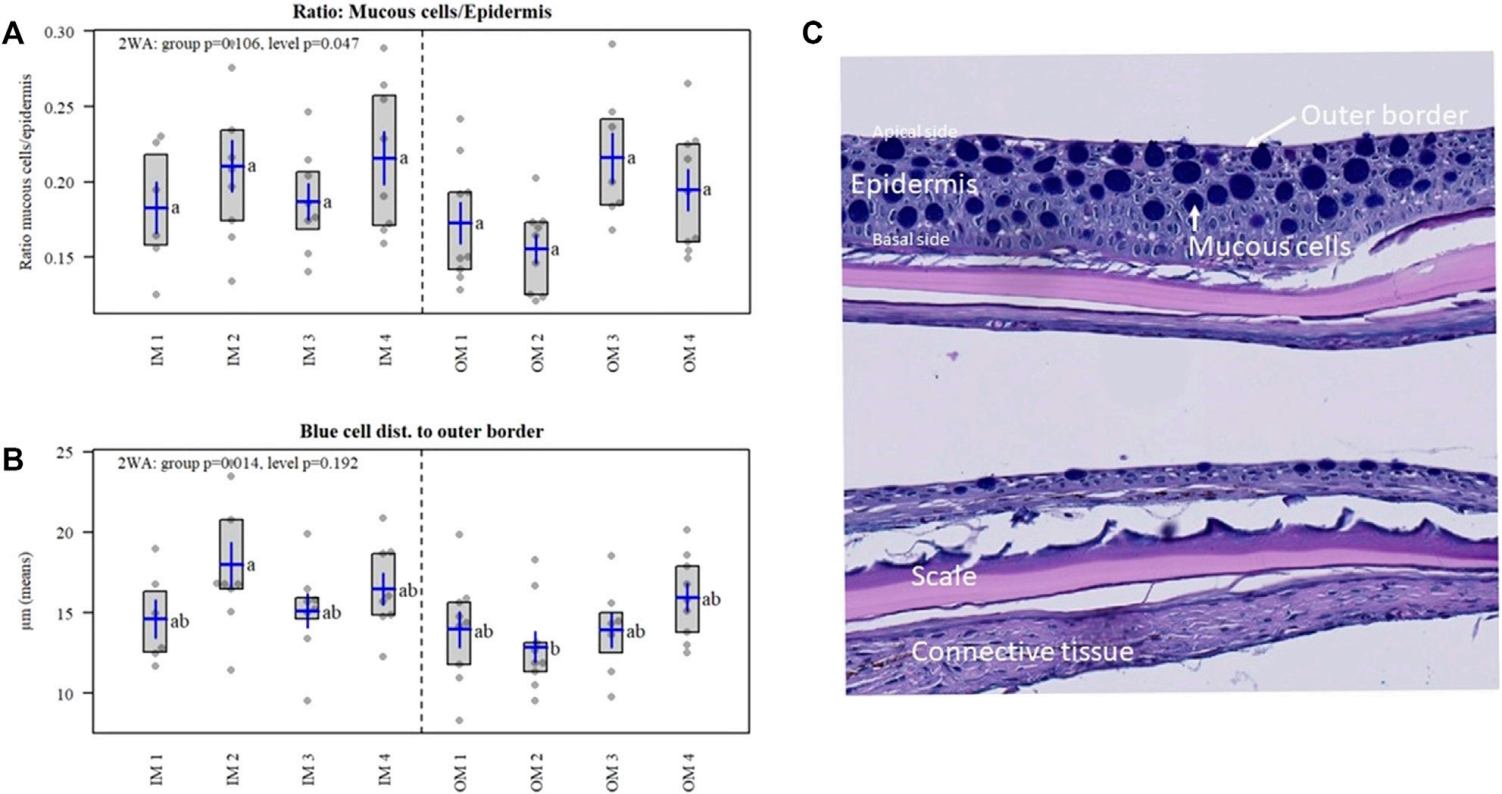
**(A)** Mucous cell ratio to epidermal area (*p* = 0.047). **(B)** Distance of blue mucous cells to the apical border in OM compared to IM (*p* = 0.014). **(C)** Histological samples of skin from Atlantic salmon in the experimental treatments of the current study.

The effects of increased levels of inorganic and organic minerals on midgut transcriptome was similar by magnitude (respectively 294 and 249 differentially expressed genes–DEG) and the composition of DEG. Correlation of the expression profiles was relatively high (Pearson r = 0.78) and differences between IM and OM were small. Several functional groups of genes showed coordinated expression changes (Figure 8), individual genes are found in Supplementary Figure S1. Higher doses of minerals stimulated different aspects of lipid metabolism: transport, activation, elongation and saturation of fatty acids, synthesis and hydrolysis of triglycerides, phospholipids and sphingomyelin, formation of lipid droplets. Trace minerals play a crucial role in the biosynthesis of lipid transport proteins by acting as essential cofactors for enzymes involved in various metabolic pathways. For instance, Cu is involved in the synthesis of apolipoprotein C-II (apoC-II), Fe is necessary for the biosynthesis of heme, a component of various proteins involved in lipid metabolism, including cytochromes and heme-containing enzymes. These proteins play roles in electron transport and redox reactions that are essential for the metabolism of lipids (e.g., in Gillard et al., 2017; Buhler, and Wang-Buhler, 1998). Manganese is a cofactor for the enzyme pyruvate carboxylase, which is involved in the synthesis of malonyl-CoA. Malonyl-CoA is a precursor for fatty acid synthesis and is necessary for the production of lipids, including those required for the assembly of lipoproteins (McGarry et al., 1978). Selenium is also essential for the synthesis of selenoproteins, some of which are involved in lipid metabolism, for example, selenoprotein P (Burk et al., 2003). Zinc is required for the activity of enzymes involved in fatty acid synthesis, such as fatty acid synthase. Additionally, zinc is involved in the stabilization of apolipoprotein B (apoB), the main structural protein of very low-density lipoproteins (VLDL) and low-density lipoproteins (LDL). Different trace mineral supplementation levels also enhanced expression of genes involved in protein biosynthesis including *trna ligases* activating arginine, cysteine, glutamine, glycine, and tyrosine. Expression changes were found in genes encoding transporters of inorganic and organic compounds and most of them were downregulated. IM and OM decreased expression of 16 and 13 genes for intracellular (cathepsins) and extracellular proteolytic enzymes including highly destructive *matrix metalloproteinases 9* and *13*. The numbers of immune genes with higher and lower expression at high levels of minerals was respectively 1 and 80 in IM and 11 and 31 in OM. Downregulation prevailed and was significantly stronger in IM (*p* = 2.1 × 10^−7^, paired *t*-test). The most affected groups were antigen presentation, antiviral responses, and immunoglobulins (17, 26, and 14 features). IM also showed tendency to stronger downregulation of xenobiotic metabolism (*p* = 0.077); lower expression was observed in genes involved in biotransformation phase I (*cytochrome p450 oxidases*) and II (*glutathione transferases*), transporters and degrading enzymes. Depending on the condition of midgut, these changes can be harmful (suppression of immune system and detoxification) or beneficial (reduction of inflammation and exposure to hazardous compounds).

### 3.9 Conclusions

Overall, our findings provide important insights into the use of organic and inorganic mineral supplements and the optimal inclusion levels in feeds for Atlantic salmon. Our results suggest that dietary OM supplementation can have a positive effect on salmon growth. Moreover, we showed that the apparent digestibility coefficients (ADC) of supplemented minerals at higher dietary levels were generally lower, except for selenium (Se) which showed an opposite trend and higher ADC in the OM than inorganic mineral (IM) groups. There was a negative correlation of ADC of phosphorus (P) and IM supplementation levels, probably related to reduced growth and tissue mineralization in these groups as compared to the organic ones, corroborated by the higher Ca and Mg levels analysed in the whole body of fish fed diets supplemented with OM. In line with literature findings, dietary supplementation of organic Se led to higher Se RE compared to inorganic Se sources. Furthermore, our results indicate that the observed selenium RE levels in Atlantic salmon surpass the maximum limits established by the EU.

Additionally, the saturation of the studied tissues in trace minerals was reached at lower dietary supplementation levels in the OM groups compared to the IM groups. At high supplementation levels, negative effects on fillet yield and quality were observed in the IM groups, while no such effect was seen in the OM groups, which in some parameters had better quality than the fish in the IM groups. Finally, mineral source and level had only minor effects on salmon skin morphology and gut transcriptome. These findings suggest that OM supplementation can offer benefits over IM in terms of growth, and fillet quality. Based on our design and the findings of mineral ADC and RE, filet technical quality and tissue mineralisation we extrapolated trace mineral supplementation recommendations for Atlantic salmon diets based on mineral source. Specifically, if organic minerals are supplemented in the diets: 10–15 ppm Cu, 348 ppm or lower Fe, 57–71 ppm Mn, 88–119 ppm Zn and 0.85 ppm Se are recommended. If inorganic minerals are used: 10–15 ppm Cu, 348 ppm or lower Fe, 57–71 ppm Mn, 119–149 ppm Zn and 0.9–0.95 ppm Se are recommended. The results of this study provide valuable information for the development of sustainable aquaculture practices and feed formulation. Future research can focus on the transport patterns of minerals in the gut and the functional effects of trace minerals on bone formation.

## Data Availability

The datasets presented in this study can be found in online repositories. The names of the repository/repositories and accession number(s) can be found below: https://www.ncbi.nlm.nih.gov/geo/query/acc.cgi?acc=GSE231575.

## References

[B1] Aas T. S. Ytrestøyl T. Åsgård T. (2019). Utilization of feed resources in the production of Atlantic Salmon (*Salmo salar)* in Norway: An update for 2016. Aquac. Rep. 15, 100216. 10.1016/j.aqrep.2019.100216

[B2] Abdallah A. G. El-Hussein O. M. Abdel-Lati K. O. (2009). Influence of some dietary organic mineral supplementations on Broiler performance. Int. J. Poult. Sci. 8 (3), 291–298. 10.3923/ijps.2009.291.298

[B3] Andersen F. Lorentzen M. Waagbø R. Maage A. (1997). Bioavailability and interactions with other micronutrients of three dietary iron sources in Atlantic salmon, *Salmo salar*, smolts. Aquac. Nutr. 3, 239–246. 10.1046/j.1365-2095.1997.00096.x

[B4] Apines M. J. Satoh S. Kiron V. Watanabe T. Aoki T. (2003). Availability of supplemental amino acid-chelated trace elements in diets containing tricalcium phosphate and phytate to rainbow trout, *Oncorhynchus mykiss* . Aquaculture 225 (1-4), 431–444. 10.1016/s0044-8486(03)00307-7

[B5] Basalo P. F. X. (2022). Potassium homeostasis and fish welfare in coupled aquaponic systems. Fish. Aquac. J. 13 (2), 1000290. 10.35248/2150-3508.22.13.290

[B6] Berntssen M. H. G. Betancor M. Caballero M. Hillestad M. Rasinger J. Hamre K. (2018). Safe limits of selenomethionine and selenite supplementation to plant-based Atlantic salmon feeds. Aquaculture 495, 617–630. 10.1016/j.aquaculture.2018.06.041

[B7] Berntssen M. H. Lundebye A. K. Hamre K. (2000). Tissue lipid peroxidative responses in Atlantic salmon (*Salmo salar* L) parr fed high levels of dietary copper and cadmium. Fish Physiology Biochem. 23 (1), 35–48. 10.1023/a:1007894816114

[B8] Betancor M. B. Dam T. M. C. Walton J. Morken T. Campbell P. J. Tocher D. R. (2016). Modulation of selenium tissue distribution and selenoprotein expression in Atlantic salmon (*Salmo salar* L) fed diets with graded levels of plant ingredients. Br. J. Nutr. 115 (8), 1325–1338. 10.1017/s0007114516000416 26907361

[B9] Bjerregaard P. Fjordside S. Hansen M. G. Petrova M. B. (2011). Dietary selenium reduces retention of methyl mercury in freshwater fish. Environ. Sci. Technol. 45 (22), 9793–9798. 10.1021/es202565g 22014184

[B10] Broadley M. Brown P. Cakmak I. Rengel Z. Zhao F. (2012). Function of nutrients. Marschner's Mineral Nutr. High. Plants, 191–248. 10.1016/b978-0-12-384905-2.00007-8

[B11] Buhler D. R. Wang-Buhler J. L. (1998). Rainbow trout cytochrome P450s: Purification, molecular aspects, metabolic activity, induction and role in environmental monitoring. Comp. Biochem. Physiology Part C Pharmacol. Toxicol. Endocrinol. 121, 107–137. 10.1016/S0742-8413(98)10033-6 9972454

[B12] Burk R. F. Hill K. E. Motley A. K. (2003). Selenoprotein metabolism and function: Evidence for more than one function for selenoprotein P. J. Nutr. 133 (5), 1517S–1520S. 10.1093/jn/133.5.1517S 12730456

[B13] Burr G. S. Wolters W. R. Barrows F. T. Donkin A. W. (2013). Evaluation of a canola protein concentrate as a replacement for fishmeal and poultry by-product meal in a commercial production diet for Atlantic Salmon (*Salmo salar*). Int. Aquatic Res. 5 (1), 5. 10.1186/2008-6970-5-5

[B14] Bury N. Grosell M. (2003). Iron acquisition by teleost fish. Comp. Biochem. Physiology Part C Toxicol. Pharmacol. 135 (2), 97–105. 10.1016/S1532-0456(03)00021-8 12860048

[B15] Chen Q.-L. Luo Z. Wu K. Huang C. Zhuo M. Q. Song Y. F. (2013). Differential induction of enzymes and genes involved in lipid metabolism in liver and visceral adipose tissue of juvenile yellow catfish *Pelteobagrus fulvidraco* exposed to copper. Aquat. Toxicol. 136, 19–28. Available at. 10.1016/j.cbpb.2015.02.004 23660017

[B16] Cho C. Y. (1992). Feeding systems for rainbow trout and other salmonids with reference to current estimates of energy and protein requirements. Aquaculture 100 (1-3), 107–123. 10.1016/0044-8486(92)90353-m

[B17] Damasceno F. M. Fleuri L. F. Sartori M. M. P. Amorim R. L. Pezzato L. E. da Silva R. L. (2016). Effect of dietary inorganic copper on growth performance and hematological profile of Nile tilapia subjected to heat-induced stress. Aquaculture 454, 257–264. 10.1016/j.aquaculture.2015.12.029

[B18] Darmawangsa G. M. Suprayudi M. A. Utomo N. P. Ekasari J. (2021). Dietary supplementation of organic selenium to improve growth performance and protein utilization in African catfish fed with different protein level diets. J. Akuakultur Indones. 20 (2), 130–138. 10.19027/jai.20.2.130-138

[B19] Dawood M. A. Alagawany M. Sewilam H. (2021). The role of zinc microelement in aquaculture: A review. Biol. trace Elem. Res. 200, 3841–3853. 10.1007/s12011-021-02958-x 34628590

[B20] Djordjevic B. Morales-Lange B. McLean Press C. Olson J. Lagos L. Mercado L. (2021). Comparison of circulating markers and mucosal immune parameters from skin and distal intestine of Atlantic salmon in two models of acute stress. Int. J. Mol. Sci. 22 (3), 1028. 10.3390/ijms22031028 33494146 PMC7864346

[B21] Domínguez D. Rimoldi S. Robaina L. E. Torrecillas S. Terova G. Zamorano M. J. (2017). Inorganic, organic, and encapsulated minerals in vegetable meal based diets for *Sparus aurata* . PeerJ 5, e3710. 10.7717/peerj.3710 29093992 PMC5661455

[B22] Eder K. Kirchgessner M. (1994). Dietary fat influences the effect of zinc deficiency on liver lipids and fatty acids in rats force-fed equal quantities of diet. J. Nutr. 124 (10), 1917–1926. 10.1093/jn/124.10.1917 7931700

[B23] Efsa Panel on Additives and Products or Substances used in Animal Feed (Feedap) (2014). Scientific Opinion on the potential reduction of the currently authorised maximum zinc content in complete feed. Efsa J. 12 (5), 3668. 10.2903/j.efsa.2014.3668 PMC13159935 42125380

[B24] El-Sayed A.-F. M. Figueiredo-Silva C. Zeid S. M. Makled S. O. (2023). Metal-amino acid complexes (ZN, Se, Cu, Fe and Mn) as a replacement of inorganic trace minerals in commercial diets for Nile tilapia (*Oreochromis niloticus*) reared under field conditions: Effects on growth, feed efficiency, gut microbiota, intestinal histology, and economic return. Aquaculture 567, 739223. 10.1016/j.aquaculture.2022.739223

[B25] Esworthy R. S. Swiderek K. M. Ho Y. S. Chu F. F. (1998). Selenium-dependent glutathione peroxidase-GI is a major glutathione peroxidase activity in the mucosal epithelium of rodent intestine. Biochimica Biophysica Acta (BBA) - General Subj. 1381 (2), 213–226. 10.1016/s0304-4165(98)00032-4 9685647

[B26] European Commission E. C. (2016). Commission implementing regulation (EU) 2016/1095 of July 2016 concerning the authorisation of zinc acetate dihydrate, zinc chloride anhydrous, zinc oxide, zinc sulphate heptahydrate, zinc sulphate monohydrate, zinc chelate of amino acid hydrate, zinc chelate of protein hydrolysates, zinc chelate of glycine hydrate (solid) and zinc chelate of glycine hydrate (liquid) as feed additives for all animal species and amending regulations (EC) No 1334/2003,(EC) No 479/2006,(EU) No 335/2010 and implementing regulations (EU) no 991/2012 and (EU) no 636/2013. Official J. Eur. Union L 182, 7–27.

[B27] European Commission E. C. (2014). Commission Implementing Regulation (EU) No 121/2014 of 7 February 2014 concerning the authorisation of L-selenomethionine as a feed additive for all animal species (Text with EEA relevance). Official J. Eur. Union L 39/53 (3).

[B28] Gaye-Siessegger J. Focken U. Abel H. Becker K. (2007). Influence of dietary non-essential amino acid profile on growth performance and amino acid metabolism of Nile tilapia, *Oreochromis niloticus* (L). Comp. Biochem. Physiology Part A Mol. Integr. Physiology 146 (1), 71–77. 10.1016/j.cbpa.2006.09.025 17157045

[B29] Gillard G. Harvey T. N. Gjuvsland A. Jin Y. Thomassen M. Lien S. (2018). Life‐stage‐associated remodelling of lipid metabolism regulation in Atlantic salmon. Mol. Ecol. 27 (5), 1200–1213. 10.1111/mec.14533 29431879

[B30] Glover C. N. Hogstrand C. (2003). Effects of dissolved metals and other hydrominerals on *in vivo* intestinal zinc uptake in freshwater rainbow trout. Aquat. Toxicol. 62 (4), 281–293. 10.1016/s0166-445x(02)00108-x 12595168

[B31] Gong J. Wang D. Shi B. Yan S. (2014). Effect of dietary organic selenium on milk selenium concentration and antioxidant and immune status in midlactation dairy cows. Livest. Sci. 170, 84–90. 10.1016/j.livsci.2014.10.003

[B32] Graff I. E. Waagbo R. Fivelstad S. Vermeer C. Lie O. Lundebye A. K. (2002). A multivariate study on the effects of dietary vitamin K, vitamin D3 and calcium, and dissolved carbon dioxide on growth, bone minerals, vitamin status and health performance in smolting Atlantic Salmon *Salmo salar* L. J. Fish Dis. 25 (10), 599–614. 10.1046/j.1365-2761.2002.00403.x

[B33] Hamre K. Bjørnevik M. Espe M. Conceição L. E. C. Johansen J. Silva J. (2020). Dietary micronutrient composition affects fillet texture and muscle cell size in Atlantic salmon (*Salmo salar*). Aquac. Nutr. 26 (3), 936–945. 10.1111/anu.13051

[B34] Hardy R. W. Sullivan C. V. Koziol A. M. (1987). Absorption, body distribution, and excretion of dietary zinc by rainbow trout (Salmo *gairdneri*). Fish Physiology Biochem. 3 (3), 133–143. 10.1007/bf02180415 24233441

[B35] Herrera M. Mancera J. M. Costas B. (2019). The use of dietary additives in fish stress mitigation: Comparative endocrine and physiological responses. Front. Endocrinol. 10, 447. 10.3389/fendo.2019.00447 PMC6636386 31354625

[B36] Iqbal F. Qureshi I. Z. Ali M. (2004). A Histopathological changes in the kidney of common carp, *Cyprinus carpio* following nitrate exposure. J. Sci. Res. 15, 411–418.

[B37] Jensen L. B. Wahli T. McGurk C. Eriksen T. B. Obach A. Waagbø R. (2015). Effect of temperature and diet on wound healing in Atlantic salmon (*Salmo salar* L). Fish Physiology Biochem. 41, 1527–1543. 10.1007/s10695-015-0105-2 26272065

[B38] Jiang J. Tang X. Xue Y. Lin G. Xiong Y. L. (2017). Dietary linseed oil supplemented with organic selenium improved the fatty acid nutritional profile, muscular selenium deposition, water retention, and tenderness of fresh pork. Meat Sci. 131, 99–106. 10.1016/j.meatsci.2017.03.014 28500964

[B39] Keenan J. O'Sullivan F. Henry M. Breen L. Doolan P. Sinkunaite I. (2018). Acute exposure to organic and inorganic sources of copper: Differential response in intestinal cell lines. Food Sci. Nutr. 6 (8), 2499–2514. 10.1002/fsn3.857 30510751 PMC6261202

[B40] Kousoulaki K. Krasnov A. Ytteborg E. Sweetman J. Pedersen M. E. Høst V. (2021). A full factorial design to investigate interactions of variable essential amino acids, trace minerals and vitamins on Atlantic Salmon Smoltification and post transfer performance. Aquac. Rep. 20, 100704. 10.1016/j.aqrep.2021.100704

[B41] Kousoulaki K. Mørkøre T. Nengas I. Berge R. Sweetman J. (2016). Microalgae and organic minerals enhance lipid retention efficiency and fillet quality in Atlantic salmon (*Salmo salar L*). Aquaculture 451, 47–57. 10.1016/j.aquaculture.2015.08.027

[B42] Kousoulaki K. Sveen L. Norén F. Espmark Å. (2022). Atlantic salmon (*Salmo salar*) performance fed low trophic ingredients in a fish meal and fish oil free diet. Front. Physiology 13, 884740. 10.3389/fphys.2022.884740 PMC9214214 35755425

[B43] Krasnov A. Timmerhaus G. Schiøtz B. L. Torgersen J. Afanasyev S. Iliev D. (2011). Genomic survey of early responses to viruses in Atlantic salmon, *Salmo salar* L. Mol. Immunol. 49 (1-2), 163–174. 10.1016/j.molimm.2011.08.007 21924497

[B44] Krogdahl Å. Penn M. Thorsen J. Refstie S. Bakke A. M. (2010). Important antinutrients in plant feedstuffs for Aquaculture: An update on recent findings regarding responses in Salmonids. Aquac. Res. 41 (3), 333–344. 10.1111/j.1365-2109.2009.02426.x

[B45] Kumar V. Sinha A. K. Makkar H. P. S. De Boeck G. Becker K. (2012). Phytate and phytase in fish nutrition. J. Animal Physiology Animal Nutr. 96 (3), 335–364. 10.1111/j.1439-0396.2011.01169.x 21692871

[B46] Labunskyy V. M. Hatfield D. L. Gladyshev V. N. (2014). Selenoproteins: Molecular pathways and physiological roles. Physiol. Rev. 94 (3), 739–777. 10.1152/physrev.00039.2013 24987004 PMC4101630

[B47] Lai S. K. Wang Y. Y. Wirtz D. Hanes J. (2009). Micro- and macrorheology of mucus. Adv. Drug Deliv. Rev. 61 (2), 86–100. 10.1016/j.addr.2008.09.012 19166889 PMC2736374

[B48] Lall S. P. (2022). The minerals. Fish. Nutr., 469–554. 10.1016/b978-0-12-819587-1.00005-7

[B49] Lansdown A. B. G. Mirastschijski U. Stubbs N. Scanlon E. Agren M. S. (2007). Zinc in wound healing: Theoretical, experimental, and clinical aspects. Wound repair Regen. 15 (1), 2–16. 10.1111/j.1524-475X.2006.00179.x 17244314

[B50] Lewis M. J. Hamid N. K. A. Alhazzaa R. Hermon K. Donald J. A. Sinclair A. J. (2013). Targeted dietary micronutrient fortification modulates N−3 LC-PUFA pathway activity in rainbow trout (*Oncorhynchus mykiss*). Aquaculture 412-413, 215–222. 10.1016/j.aquaculture.2013.07.024

[B51] Li R. Wen Y. Lin G. Meng C. Wang F. (2019). Different sources of copper effect on intestinal epithelial cell: Toxicity, oxidative stress, and metabolism. Metabolites 10 (1), 11. 10.3390/metabo10010011 31877957 PMC7022486

[B52] Lim C. Klesius P. H. Shoemaker C. A. (2001). “Dietary iron and fish health,” in Nutrition and fish health (Binghamton, New York: The Haworth Press, Inc.), 189–199.

[B53] Lin H. C. Sung W. T. (2003). The distribution of mitochondria‐rich cells in the gills of air‐breathing fishes. Physiological Biochem. Zoology 76 (2), 215–228. 10.1086/374278 12794675

[B54] Lin Y.-H. Shie Y.-Y. Shiau S.-Y. Lynn D. G. (2008). Capturing the VirA/VirG TCS of agrobacterium tumefaciens. Aquaculture 274 (1), 161–177. 10.1007/978-0-387-78885-2_11

[B55] Lin Y.-H. Shih C. C. Kent M. Shiau S. Y. (2010). Dietary copper requirement reevaluation for juvenile grouper, E*pinephelus malabaricus*, with an organic copper source. Aquaculture 310 (1-2), 173–177. 10.1016/j.aquaculture.2010.10.004

[B56] Lygren B. Hamre K. Waagbø R. (1999). Effects of dietary pro- and antioxidants on some protective mechanisms and health parameters in Atlantic Salmon. J. Aquatic Animal Health 11 (3), 211–221. 10.1577/1548-8667(1999)011<0211:eodpaa>2.0.co;2

[B57] Maage A. Julshamn K. Berge G. E. (2001). Zinc gluconate and zinc sulphate as dietary zinc sources for Atlantic Salmon. Aquac. Nutr. 7 (3), 183–187. 10.1046/j.1365-2095.2001.00170.x

[B58] Maage A. Sveier H. (1998). Addition of dietary iron (III) oxide does not increase iron status of growing Atlantic salmon. Aquac. Int. 6, 249–252. 10.1023/a:1009258828313

[B59] Mahfouz M. M. Kummerow F. A. (1989). Effect of magnesium deficiency on Δ6 desaturase activity and fatty acid composition of rat liver microsomes. Lipids 24 (8), 727–732. 10.1007/BF02535212 2555646

[B60] Marta del Puerto M. Cabrera M. C. Saadoun A. (2017). A note on fatty acids profile of meat from broiler chickens supplemented with inorganic or organic selenium. Int. J. Food Sci. 2017, 1–8. 10.1155/2017/7613069 PMC5282453 28194404

[B61] McGarry J. D. Takabayashi Y. Foster D. W. (1978). The role of malonyl-coa in the coordination of fatty acid synthesis and oxidation in isolated rat hepatocytes. J. Biol. Chem. 253 (22), 8294–8300. 10.1016/S0021-9258(17)34395-8 711753

[B62] Meiler K. A. Cleveland B. Radler L. Kumar V. (2021). Oxidative stress-related gene expression in diploid and triploid rainbow trout (*Oncorhynchus mykiss*) fed diets with organic and inorganic zinc. Aquaculture 533, 736149. 10.1016/j.aquaculture.2020.736149

[B63] Mohseni M. Pourkazemi M. Bai S. C. (2014). Effects of dietary inorganic copper on growth performance and immune responses of juvenile beluga, *Huso huso* . Aquac. Nutr. 20 (5), 547–556. 10.1111/anu.12107

[B64] Mommsen T. P. Vijayan M. M. Moon T. W. (1999). Cortisol in teleosts: Dynamics, mechanisms of action, and metabolic regulation. Rev. fish Biol. Fish. 9, 211–268. 10.1023/A:1008924418720

[B65] Nakatani R. E. (1966). “Biological response of rainbow trout (*Salmo gairdneri*) ingesting Zn-65,” in Proceedings, symposium on the disposal of Radioactive= astes into seas (Vienna, Austria: International Atomic Energy Agency).

[B66] National Research Council (2011). Nutrient requirements of fish and shrimp. Washington, DC, USA: The National Academies Press.

[B67] Nguyen L. Kubitza F. Salem S. M. Hanson T. R. Allen Davis D. (2019). Comparison of organic and inorganic microminerals in all plant diets for Nile tilapia *Oreochromis niloticus* . Aquaculture 498, 297–304. 10.1016/j.aquaculture.2018.08.034

[B68] Noble C. Gismervik K. Iversen M. H. Kolarevic J. Nilsson J. Stien L. H. (2018). Welfare indicators for farmed Atlantic salmon: Tools for assessing fish welfare. Available at https://nofima.no/publikasjon/1636395/95.

[B69] Olmedo P. Hernández A. F. Pla A. Femia P. Navas-Acien A. Gil F. (2013). Determination of essential elements (copper, manganese, selenium and zinc) in fish and shellfish samples. risk and nutritional assessment and Mercury–selenium balance. Food Chem. Toxicol. 62, 299–307. 10.1016/j.fct.2013.08.076 24007738

[B70] Osumi T. Hashimoto T. (1979). Peroxisomal βoxidation system of rat liver. copurification of enoyl-COA hydratase and 3-hydroxyacyl-COA dehydrogenase. Biochem. Biophysical Res. Commun. 89 (2), 580–584. 10.1016/0006-291x(79)90669-7 486181

[B71] Pan C. Huang K. Zhao Y. Qin S. Chen F. Hu Q. (2007). Effect of selenium source and level in hen's diet on tissue selenium deposition and egg selenium concentrations. J. Agric. Food Chem. 55 (3), 1027–1032. 10.1021/jf062010a 17263508

[B72] Pierri B. da Silva A. D. Cadorin D. I. Ferreira T. H. Mouriño J. L. P. Filer K. (2020). Different levels of organic trace minerals in diets for Nile tilapia juveniles alter gut characteristics and body composition, but not growth. Aquac. Nutr. 27 (1), 176–186. 10.1111/anu.13175

[B73] Poirier Y. Antonenkov V. D. Glumoff T. Hiltunen J. K. (2006). Peroxisomal β-oxidation—A metabolic pathway with multiple functions. Biochimica Biophysica Acta (BBA) - Mol. Cell Res. 1763 (12), 1413–1426. 10.1016/j.bbamcr.2006.08.034 17028011

[B74] Prabhu A. J. Holen E. Espe M. Silva M. S. Holme M. H. Hamre K. (2020). Dietary selenium required to achieve body homeostasis and attenuate pro-inflammatory responses in Atlantic salmon post-smolt exceeds the present EU legal limit. Aquaculture 526, 735413. 10.1016/j.aquaculture.2020.735413

[B75] Prabhu A. J. Lock E. J. Hemre G. I. Hamre K. Espe M. Olsvik P. A. (2019a). Recommendations for dietary level of micro-minerals and vitamin D3 to Atlantic salmon (*Salmo salar*) parr and post-smolt when fed low fish meal diets. PeerJ 7, e6996. 10.7717/peerj.6996 31183254 PMC6546083

[B76] Prabhu A. J. Schrama J. W. Kaushik S. J. (2016). Mineral requirements of fish: A systematic review. Rev. Aquac. 8 (2), 172–219. 10.1111/raq.12090

[B77] Prabhu A. J. Silva M. S. Kröeckel S. Holme M. H. Ørnsrud R. Amlund H. (2019b). Effect of levels and sources of dietary manganese on growth and mineral composition of post-smolt Atlantic salmon fed low fish meal, plant-based ingredient diets. Aquaculture 512, 734287. 10.1016/j.aquaculture.2019.734287

[B78] Raynal B. D. E. Hardingham T. E. Sheehan J. K. Thornton D. J. (2003). Calcium-dependent protein interactions in MUC5B provide reversible cross-links in salivary mucus. J. Biol. Chem. 278 (31), 28703–28710. 10.1074/jbc.m304632200 12756239

[B79] Rigos G. Samartzis A. Henry M. Fountoulaki E. Cotou E. Sweetman J. (2010). Effects of additive iron on growth, tissue distribution, haematology and immunology of gilthead sea bream, *Sparus aurata* . Aquac. Int. 18, 1093–1104. 10.1007/s10499-010-9326-7

[B80] Saibu Y. Jamwal A. Feng R. Peak D. Niyogi S. (2018). Distribution and speciation of zinc in the gills of rainbow trout (*Oncorhynchus mykiss)* during acute waterborne zinc exposure: Interactions with cadmium or copper. Comp. Biochem. Physiology Part C Toxicol. Pharmacol. 206-207, 23–31. 10.1016/j.cbpc.2018.02.004 29501824

[B81] Sandnes K. Lie O. Waagbo R. (1988). Normal ranges of some blood chemistry parameters in adult farmed Atlantic salmon, *Salmo salar* . J. Fish Biol. 32 (1), 129–136. 10.1111/j.1095-8649.1988.tb05341.x

[B82] Sandodden R. Finstad B. Iversen M. (2001). Transport stress in atlantic salmon (*Salmo salar* L): Anaesthesia and recovery. Aquac. Res. 32 (2), 87–90. 10.1046/j.1365-2109.2001.00533.x

[B83] Scholz R. W. Roy A. H. Hellums D. T. (2014). “Sustainable phosphorus management: A transdisciplinary challenge,” in Sustainable phosphorus management (Berlin, Germany: Springer). 10.1007/978-94-007-7250-2_1

[B84] Schreck C. B. Tort L. (2016). The concept of stress in fish. Fish. Physiol. 35, 1–34. 10.1016/B978-0-12-802728-8.00001-1

[B85] Sele V. Ørnsrud R. Sloth J. J. Berntssen M. H. G. Amlund H. (2018). Selenium and selenium species in feeds and muscle tissue of Atlantic Salmon. J. Trace Elem. Med. Biol. 47, 124–133. 10.1016/j.jtemb.2018.02.005 29544799

[B86] Sigurgisladottir S. Øtorrissen O. Lie Ø. Thomassen M. Hafsteinsson H. (1997). Salmon Quality: Methods to determine the quality parameters. Rev. Fish. Sci. 5 (3), 223–252. 10.1080/10641269709388599

[B87] Silva M. S. Kröckel S. Jesu Prabhu P. A. Koppe W. Ørnsrud R. Waagbø R. (2019). Apparent availability of zinc, selenium and manganese as inorganic metal salts or organic forms in plant-based diets for Atlantic salmon (*Salmo salar*). Aquaculture 503, 562–570. 10.1016/j.aquaculture.2019.01.005

[B89] Standal H. Dehli A. Rørvik K. -A. Andersen Ø. (1999). Iron status and dietary levels of iron affect the bioavailability of haem and nonhaem iron in Atlantic Salmon, *Salmo salar* . Aquac. Nutr. 5 (3), 193–198. 10.1046/j.1365-2095.1999.00105.x

[B90] Stangl G. I. Kirchgessner M. (1998). Effect of different degrees of moderate iron deficiency on the activities of tricarboxylic acid cycle enzymes, and the cytochrome oxidase, and the iron, copper, and zinc concentrations in rat tissues. Z. für Ernährungswiss. 37 (3), 260–268. 10.1007/s003940050025 9800317

[B91] Sun S. Qin J. Yu N. Ge X. Jiang H. Chen L. (2013). Effect of dietary copper on the growth performance, non-specific immunity and resistance to *aeromonas hydrophila* of juvenile Chinese mitten crab, E*riocheir sinensis* . Fish Shellfish Immunol. 34 (5), 1195–1201. 10.1016/j.fsi.2013.01.021 23422815

[B92] Sveen L. Krasnov A. Timmerhaus G. Bogevik A. S. (2021). Responses to mineral supplementation and Salmon Lice (*Lepeophtheirus Salmonis*) infestation in skin layers of Atlantic salmon (*Salmo salar* L). Genes 12 (4), 602. 10.3390/genes12040602 33921813 PMC8073069

[B93] Tan X. Y. Luo Z. Liu X. Xie C. X. (2011). Dietary copper requirement of juvenile yellow catfish P*elteobagrus fulvidraco* . Aquac. Nutr. 17 (2), 170–176. 10.1111/j.1365-2095.2009.00720.x

[B94] Tang L. Huang K. Xie J. Yu D. Sun L. Huang Q. (2017). Dietary copper affects antioxidant and immune activity in hybrid tilapia (*Oreochromis niloticus* × *Oreochromis aureus*). Aquac. Nutr. 23 (5), 1003–1015. 10.1111/anu.12468

[B95] Taylor J. F. Vera L. M. De Santis C. Lock E. J. Espe M. Skjærven K. H. (2019). The effect of micronutrient supplementation on growth and hepatic metabolism in diploid and triploid Atlantic salmon (*Salmo salar*) parr fed a low marine ingredient diet. Comp. Biochem. Physiology Part B Biochem. Mol. Biol. 227, 106–121. 10.1016/j.cbpb.2018.10.004 30367964

[B97] Torstensen B. E. Espe M. Stubhaug I. Lie Ø. (2011). Dietary plant proteins and vegetable oil blends increase adiposity and plasma lipids in Atlantic salmon (*Salmo salar* L). Br. J. Nutr. 106 (5), 633–647. 10.1017/s0007114511000729 21535902

[B98] Tseng Y. Dominguez D. Bravo J. Acosta F. Robaina L. Geraert P. A. (2021). Organic selenium (OH-metse) effect on whole body fatty acids and MX gene expression against viral infection in gilthead seabream (*Sparus aurata*) juveniles. Animals 11 (10), 2877. 10.3390/ani11102877 34679898 PMC8532762

[B99] Vera L. M. Hamre K. Espe M. Hemre G. I. Skjærven K. Lock E. J. (2020). Higher dietary micronutrients are required to maintain optimal performance of Atlantic salmon (*Salmo salar*) fed a high plant material diet during the full production cycle. Aquaculture 528, 735551. 10.1016/j.aquaculture.2020.735551

[B100] Watanabe T. Kiron V. Satoh S. (1997). Trace minerals in fish nutrition. Aquaculture 151 (1-4), 185–207. 10.1016/s0044-8486(96)01503-7

[B101] Yarahmadi S. S. Silva M. S. Holme M. H. Morken T. Remø S. Araujo P. (2022). Impact of dietary zinc and seawater transfer on zinc status, availability, endogenous loss and osmoregulatory responses in Atlantic salmon smolt fed low fish meal feeds. Aquaculture 549, 737804. 10.1016/j.aquaculture.2021.737804

[B102] Ytrestøyl T. Aas T. S. Åsgård T. (2015). Utilisation of feed resources in production of Atlantic Salmon (*Salmo salar*) in Norway. Aquaculture 448, 365–374. 10.1016/j.aquaculture.2015.06.023

[B103] Zhang L. Feng L. Jiang W. D. Liu Y. Jiang J. Li S. H. (2016). The impaired flesh quality by iron deficiency and excess is associated with increasing oxidative damage and decreasing antioxidant capacity in the muscle of young grass carp (*Ctenopharyngodon idellus*). Aquac. Nutr. 22, 191–201. 10.1111/anu.12237

[B104] Zhao Z. Barcus M. Kim J. Lum K. L. Mills C. Lei X. G. (2016). High dietary selenium intake alters lipid metabolism and protein synthesis in liver and muscle of pigs. J. Nutr. 146 (9), 1625–1633. 10.3945/jn.116.229955 27466604 PMC4997278

[B105] Zhou Y. E. Kubow S. Egeland G. M. (2011). Is iron status associated with highly unsaturated fatty acid status among Canadian Arctic Inuit? Food & Funct. 2 (7), 381–385. 10.1039/c1fo10051c 21894324

[B106] Zikos A. Seale A. P. Lerner D. T. Grau E. G. Korsmeyer K. E. (2014). Effects of salinity on metabolic rate and branchial expression of genes involved in ion transport and metabolism in Mozambique tilapia (*Oreochromis mossambicus*). Comp. Biochem. Physiology Part A Mol. Integr. Physiology 178, 121–131. 10.1016/j.cbpa.2014.08.016 25193178

