# Supplementary material for: Optimisation of trace mineral supplementation in diets for Atlantic salmon smolt with reference to holistic fish performance in terms of growth, health, welfare, and potential environmental impacts
Source: Front Physiol. 2023 Aug 17;14:1214987. doi: 10.3389/fphys.2023.1214987 (PMC10469859; doi:10.3389/fphys.2023.1214987)
Supplement: Supplementary file 1 [file DataSheet1.docx]

Supplementary-Table 1 Salmon feeding trial experimental diet formulations.

| Diet Name | IM 1 | IM 2 | IM 3 | IM 4 | OM 1 | OM 2 | OM 3 | OM 4 |
| --- | --- | --- | --- | --- | --- | --- | --- | --- |
|  | % | % | % | % | % | % | % | % |
| SPC | 22.0 | 22.0 | 22.0 | 22.0 | 22.0 | 22.0 | 22.0 | 22.0 |
| Wheat gluten | 19.22 | 19.22 | 19.22 | 19.22 | 19.22 | 19.22 | 19.22 | 19.22 |
| Horse beans | 11.50 | 11.50 | 11.50 | 11.50 | 11.50 | 12.16 | 11.50 | 11.50 |
| Fish oil | 13.3 | 13.3 | 13.3 | 13.3 | 13.3 | 13.3 | 13.3 | 13.3 |
| Fishmeal | 10.0 | 10.0 | 10.0 | 10.0 | 10.0 | 10.0 | 10.0 | 10.0 |
| Rapeseed oil | 9.7 | 9.7 | 9.7 | 9.7 | 9.7 | 9.7 | 9.7 | 9.7 |
| Soya lecithin | 2.6 | 2.6 | 2.6 | 2.6 | 2.6 | 2.6 | 2.6 | 2.6 |
| NaH_2_PO_4_ | 2.5 | 2.5 | 2.5 | 2.5 | 2.5 | 2.5 | 2.5 | 2.5 |
| Krill hydrolysate | 2.0 | 2.0 | 2.0 | 2.0 | 2.0 | 2.0 | 2.0 | 2.0 |
| Vitamin mix | 2.0 | 2.0 | 2.0 | 2.0 | 2.0 | 2.0 | 2.0 | 2.0 |
| Lys (79 %. 19.41 % Cl) | 1.7 | 1.7 | 1.7 | 1.7 | 1.7 | 1.7 | 1.7 | 1.7 |
| Histidine | 0.6 | 0.6 | 0.6 | 0.6 | 0.6 | 0.6 | 0.6 | 0.6 |
| Choline chloride | 0.5 | 0.5 | 0.5 | 0.5 | 0.5 | 0.5 | 0.5 | 0.5 |
| Cholesterol | 0.5 | 0.5 | 0.5 | 0.5 | 0.5 | 0.5 | 0.5 | 0.5 |
| Methionine 99% | 0.46 | 0.46 | 0.46 | 0.46 | 0.46 | 0.46 | 0.46 | 0.46 |
| Threonine 98.5% | 0.42 | 0.42 | 0.42 | 0.42 | 0.42 | 0.42 | 0.42 | 0.42 |
| MgHPO_4_^+3^H2O (+0.3125%) + K2CO3 (+0.1413%) | 0.4538 | 0.4538 | 0.4538 | 0.4538 | 0.4538 | 0.4538 | 0.4538 | 0.4538 |
| Stay-C | 0.25 | 0.25 | 0.25 | 0.25 | 0.25 | 0.25 | 0.25 | 0.25 |
| Carop. Pink (10%) | 0.05 | 0.05 | 0.05 | 0.05 | 0.05 | 0.05 | 0.05 | 0.05 |
| Yttrium oxide | 0.01 | 0.01 | 0.01 | 0.01 | 0.01 | 0.01 | 0.01 | 0.01 |
| CuSO4+5H2O | 0.0019 | 0.0039 | 0.0059 | 0.0078 |  |  |  |  |
| FeSO4+7 H2O | 0.0613 | 0.0946 | 0.1284 | 0.1618 |  |  |  |  |
| ZnSO4 - H2O | 0.0119 | 0.0206 | 0.0291 | 0.0378 |  |  |  |  |
| MnSO4+H2O | 0.0072 | 0.0115 | 0.0161 | 0.0204 |  |  |  |  |
| Na2SeO3 | 0.0005 | 0.00057 | 0.00068 | 0.00079 |  |  |  |  |
| Copper organic |  |  |  |  | 0.0041 | 0.0082 | 0.0124 | 0.0166 |
| Iron organic |  |  |  |  | 0.0821 | 0.1268 | 0.1721 | 0.2168 |
| Zinc organic |  |  |  |  | 0.0282 | 0.0488 | 0.0688 | 0.0895 |
| Manganese organic |  |  |  |  | 0.0155 | 0.0248 | 0.0348 | 0.0442 |
| Selenium organic |  |  |  |  | 0.01038 | 0.01288 | 0.01538 | 0.01788 |
| Sum | 100 | 100 | 100 | 100 | 100 | 100 | 100 | 100 |

Supplementary-Table 2 Chemical composition of the experimental diets used in the feeding trial of Atlantic salmon smolt

| **Diet Name** |  | **IM 1** | **IM 2** | **IM 3** | **IM 4** | **OM 1** | **OM 2** | **OM 3** | **OM 4** |
| --- | --- | --- | --- | --- | --- | --- | --- | --- | --- |
| Copper* | mg/kg | 9.3 | 13 | 19 | 24 | 9.7 | 14 | 19 | 24 |
| Iron** | mg/kg | 300±4 | 386±22 | 458±9 | 509±12 | 302±5 | 374±13 | 420±13 | 502±45 |
| Zinc** | mg/kg | 78±6 | 107±11 | 133±13 | 161±15 | 87±7 | 118±11 | 149±17 | 176±19 |
| Manganese** | mg/kg | 57±6 | 66±4 | 80±4 | 99±3 | 55±2 | 71±2 | 83±3 | 96±3 |
| Selenium** | mg/kg | 1.20±0.05 | 1.30±0.07 | 1.46±0.13 | 1.47±0.06 | 1.36±0.08 | 1.38±0.04 | 1.45±0.08 | 1.51±0.05 |
| Selenium* | mg/kg | 0.7 | 0.9 | 1.2 | 1.1 | 1.0 | 1.2 | 0.9 | 1.0 |
| Total Phosphorus*** | % | 1.2 | 1.2 | 1.2 | 1.2 | 1.1 | 1.1 | 1.2 | 1.2 |
| Yttrium* | mg/kg | 77 | 75 | 80 | 78 | 74 | 79 | 83 | 81 |
| Free astaxanthin*** | mg/kg | 40 | 42 | 42 | 40 | 40 | 40 | 40 | 40 |
| Moisture *** | % | 7.8 | 6.4 | 6.8 | 7.2 | 7.5 | 7.7 | 7.5 | 7.3 |
| Protein*** | % | 43.3 | 43.9 | 43.5 | 43.1 | 43.1 | 43.5 | 43.4 | 43.8 |
| Fat*** | % | 28.9 | 29.7 | 29.0 | 28.8 | 29.7 | 29.3 | 29.0 | 29.0 |
| Energy*** | KJ/g | 23.08 | 23.33 | 23.06 | 23.4 | 23.06 | 23.28 | 23.49 | 23.11 |
| **Fatty acids (FA) as % in Bligh & Dyer extract** | | | | | | | | | |
| 14:0 | % | 3 | 2.7 | 2.8 | 2.8 | 2.8 | 3 | 3.1 | 3.1 |
| 16:0 | % | 9.4 | 9 | 9.5 | 9 | 9.2 | 9.4 | 9.7 | 9.8 |
| 18:0 | % | 1.7 | 1.6 | 1.7 | 1.7 | 1.7 | 1.6 | 1.7 | 1.7 |
| 20:0 | % | 0.3 | 0.3 | 0.3 | 0.3 | 0.3 | 0.3 | 0.3 | 0.3 |
| 22:0 | % | 0.2 | 0.2 | 0.2 | 0.2 | 0.2 | 0.2 | 0.2 | 0.2 |
| **Total saturated fatty acids** | **%** | **14.6** | **13.8** | **14.5** | **14** | **14.2** | **14.5** | **15** | **15.1** |
| 16:1 n-7 | % | 2.7 | 2.6 | 2.7 | 2.6 | 2.7 | 2.8 | 2.9 | 2.9 |
| 18:1 (n-9)+(n-7)+(n-5) | % | 29 | 29.3 | 30.9 | 30.3 | 30.5 | 27.8 | 28 | 28.5 |
| 20:1 (n-9)+(n-7) | % | 6.6 | 6.2 | 6.3 | 6.5 | 6.6 | 6.7 | 7.1 | 7.1 |
| 22:1 (n-11)+(n-9)+(n-7) | % | 7.8 | 7.2 | 7.4 | 7.3 | 7.4 | 7.8 | 8 | 8.2 |
| 24:1 n-9 | % | 0.4 | 0.4 | 0.4 | 0.4 | 0.4 | 0.4 | 0.4 | 0.4 |
| **Total monosaturated fatty acids** | **%** | **46.5** | **45.7** | **47.7** | **47.1** | **47.6** | **45.5** | **46.4** | **47.1** |
| 16:2 n-4 | % | 0.1 | 0.1 | 0.1 | 0.1 | 0.1 | 0.1 | 0.1 | 0.1 |
| 16:3 n-4 | % | 0.1 | 0.1 | 0.1 | 0.1 | 0.1 | 0.1 | 0.1 | 0.1 |
| 18:2 n-6 | % | 12.8 | 12.9 | 13.5 | 13.2 | 13.2 | 12.3 | 12.5 | 12.6 |
| 18:3 n-6 | % | 0.1 | 0.1 | 0 | 0.1 | 0.1 | 0.1 | 0 | 0.1 |
| 20:2 n-6 | % | 0.2 | 0.1 | 0.1 | 0.1 | 0.1 | 0.2 | 0.2 | 0.2 |
| 20:3 n-6 | % | 0 | 0 | 0 | 0 | 0 | 0 | 0 | 0 |
| 20:4 n-6 | % | 0.1 | 0.1 | 0.1 | 0.1 | 0.1 | 0.1 | 0.1 | 0.2 |
| 22:4 n-6 | % | 0 | 0 | 0 | 0 | 0 | 0 | 0 | 0 |
| **Total n-6 polyunsaturated FA** | **%** | **13.2** | **13.2** | **13.7** | **13.5** | **13.5** | **12.7** | **12.8** | **13.1** |
| 18:3 n-3 | % | 4 | 4.1 | 4.3 | 4.2 | 4.3 | 3.8 | 3.8 | 3.8 |
| 18:4 n-3 | % | 1.1 | 1 | 1 | 1 | 1 | 1.1 | 1.1 | 1.1 |
| 20:3 n-3 | % | 0 | 0 | 0 | 0 | 0.1 | 0 | 0 | 0 |
| 20:4 n-3 | % | 0.2 | 0.2 | 0.2 | 0.2 | 0.2 | 0.2 | 0.2 | 0.2 |
| 20:5 n-3 (EPA) | % | 3.2 | 2.8 | 3 | 2.9 | 2.9 | 3.1 | 3.2 | 3.3 |
| 21:5 n-3 | % | 0.1 | 0.1 | 0.1 | 0.1 | 0.1 | 0.1 | 0.1 | 0.1 |
| 22:5 n-3 | % | 0.4 | 0.4 | 0.4 | 0.4 | 0.4 | 0.4 | 0.4 | 0.5 |
| 22:6 n-3 (DHA) | % | 4 | 3.7 | 3.8 | 3.7 | 3.8 | 3.9 | 4.1 | 4.1 |
| **Total n-3 polyunsaturated FA** | **%** | **13** | **12.3** | **12.8** | **12.5** | **12.8** | **12.6** | **12.9** | **13.1** |
| omega-6/omega-3 |  | 1 | 1.07 | 1.08 | 1.08 | 1.06 | 1.01 | 0.99 | 0.98 |
| EPA + DHA | % | 7.2 | 6.5 | 6.8 | 6.6 | 6.7 | 7 | 7.3 | 7.4 |
| Identified fatty acids | % | 87.5 | 85.5 | 88.9 | 87.3 | 88.3 | 85.5 | 87.3 | 88.6 |
| Unidentified fatty acids | % | 3.4 | 3.1 | 3 | 3.2 | 3.3 | 3.3 | 3.5 | 3.4 |

*Analysed at Eurofins WEJ Contaminants, Hamburg, Germany

**Analysed at Alltech Inc, Dunboyne, Ireland

***Analysed at Nofima’s

Supplementary-Table 3 Atlantic salmon growth and feed utilisation performance when fed diets (8 treatments (T)) with variable source (S) and levels (L) of trace minerals^*^ (values are means± standard variation; n=3 tanks). Values with significant differences or trends are marked bold.

|  |  |  |  |  |  |  |  |  | **p-values*** | | | |
| --- | --- | --- | --- | --- | --- | --- | --- | --- | --- | --- | --- | --- |
|  | **IM1** | **IM2** | **IM3** | **IM4** | **OM1** | **OM2** | **OM3** | **OM4** | **T** | **S** | **L** | **S x L** |
| Start No | 55 | 55 | 55 | 55 | 55 | 55 | 55 | 55 |  |  |  |  |
| Mortality | 0 | 0 | 0 | 0 | 0 | 0 | 0 | 0 |  |  |  |  |
| Start BW (g) | 216±1 | 214±0 | 215±1 | 216±1 | 215±1 | 216±2 | 215±2 | 216±1 | 0.779 | 0.935 | 0.932 | 0.356 |
| **MBW1 (g)** | 370±8 | 370±1 | 365±19 | 361±8 | **377±5** | **385±5** | **374±12** | **372±6** | 0.150 | **0.016^⭘^** | 0.279 | 0.896 |
| **MBW2 (g)** | 413±8 | 412±3 | 405±23 | 405±10 | **422±8** | **432±10** | **419±16** | **411±8** | 0.191 | **0.025^⭘^** | 0.309 | 0.791 |
| **MBW3 (g)** | 461±10 | 455±5 | 454±25 | 448±14 | **457±12** | **482±15** | **461±16** | **459±8** | 0.258 | **0.098^⭘^** | 0.399 | 0.357 |
| **Final BW (g)** | 680±45 | 693±2 | 697±47 | 694±19 | **702±23** | **730±30** | **710±27** | **708±10** | 0.607 | **0.087^⭘^** | 0.681 | 0.879 |
| **Total SGR** | 1.38±0.08 | 1.41±0.- | 1.41±0.08 | 1.41±0.04 | **1.43±0.04** | **1.47±0.05** | **1.44±0.04** | **1.43±0.02** | 0.602 | **0.082^⭘^** | 0.638 | 0.935 |
| **TGC1** | 3.14±0.17 | 3.20±0.02 | 3.08±0.31 | 3.01±0.15 | **3.31±0.07** | **3.42±0.12** | **3.23±0.17** | **3.19±0.10** | 0.128 | **0.013^⭘^** | 0.217 | 0.986 |
| TGC2 | 5.16±0.13 | 4.97±0.40 | 4.85±0.37 | 5.29±0.66 | 5.35±0.34 | 5.40±0.57 | 5.34±0.50 | 4.72±0.25 | 0.436 | 0.475 | 0.800 | 0.171 |
| **TGC3** | **6.40^b^± 0.23** | **5.79^b^± 0.34** | **6.54^b^± 0.09** | **5.79^b^± 0.50** | **4.61^a^± 1.32** | **6.42^b^± 0.66** | **5.60^ab^± 0.48** | **6.39^b^± 0.45** | **0.023** | 0.265 | 0.527 | **0.008** |
| TGC4 | 4.18±0.70 | 4.54±0.15 | 4.63±0.24 | 4.72±0.00 | 4.65±0.12 | 4.58±0.24 | 4.69±0.12 | 4.71±0.04 | 0.408 | 0.260 | 0.316 | 0.479 |
| **Total TGC** | 3.85±0.27 | 3.95±0.01 | 3.96±0.26 | 3.94±0.13 | **4.00±0.14** | **4.15±0.17** | **4.04±0.13** | **4.02±0.07** | 0.604 | **0.083^⭘^** | 0.650 | 0.923 |
| **Total feed/fish** | 270±9 | 268±16 | 265±25 | 261±9 | **273±13** | **285±18** | **272±13** | **274±7** | 0.668 | **0.080^⭘^** | 0.719 | 0.840 |
| FCR1 | 0.83±0.02 | 0.83±0.02 | 0.82±0.02 | 0.84±0.03 | 0.80±0.05 | 0.82±0.01 | 0.81±0.02 | 0.83±0.01 | 0.626 | 0.165 | 0.366 | 0.928 |
| FCR2 | 0.59±0.03 | 0.62±0.07 | 0.58±0.03 | 0.55±0.07 | 0.55±0.01 | 0.60±0.10 | 0.56±0.05 | 0.63±0.03 | 0.537 | 0.982 | 0.610 | 0.267 |
| **FCR3** | **0.44^a^± 0.01** | **0.46^a^± 0.02** | **0.43^a^± 0.04** | **0.46^a^± 0.03** | **0.66^b^± 0.20** | **0.48^a^± 0.04** | **0.49^a^± 0.03** | **0.44^a^± 0.03** | **0.039** | **0.047^⭘^** | 0.231 | **0.070** |
| FCR4 | 0.56±0.12 | 0.48±0.07 | 0.49±0.01 | 0.48±0.01 | 0.48±0.01 | 0.51±0.02 | 0.48±0.01 | 0.49±0.01 | 0.436 | 0.488 | 0.465 | 0.288 |
| Total FCR DM | 0.58±0.05 | 0.56±0.03 | 0.55±0.00 | 0.55±0.01 | 0.56±0.00 | 0.56±0.01 | 0.55±0.00 | 0.56±0.01 | 0.562 | 0.661 | 0.283 | 0.577 |
| Total FE | 1.58±0.14 | 1.66±0.10 | 1.68±0.01 | 1.68±0.02 | 1.65±0.01 | 1.65±0.02 | 1.68±0.01 | 1.66±0.03 | 0.590 | 0.752 | 0.283 | 0.603 |

^*^Values in the same row with different small letter are significantly different (p<0.05) following Duncan’s HSD post hoc test

^⭘^OM>IM

Supplementary-Table 4 Atlantic salmon growth and feed utilisation performance when fed diets with added trace minerals in either inorganic (IM) or organic (OM) form at variable levels^*^ (values are means± standard variation; n=3 tanks). Values with significant differences or trends are marked bold.

|  | **IM1** | **IM2** | **IM3** | **IM4** | **p values*** | **OM1** | **OM2** | **OM3** | **OM4** | **p values*** |
| --- | --- | --- | --- | --- | --- | --- | --- | --- | --- | --- |
| start no | 55 | 55 | 55 | 55 |  | 55 | 55 | 55 | 55 |  |
| mortality (%) | 0 | 0 | 0 | 0 |  | 0 | 0 | 0 | 0 |  |
| start bw (g) | 216±1 | 214±0 | 215±1 | 216±1 | 0.255 | 215±1 | 216±2 | 215±2 | 216±1 | 0.835 |
| MBW1 (g) | 370±8 | 370±1 | 365±19 | 361±8 | 0.729 | 377±5 | 385±5 | 374±12 | 372±6 | 0.196 |
| MBW2 (g) | 413±8 | 412±3 | 405±23 | 405±10 | 0.831 | 422±8 | 432±10 | 419±16 | 411±8 | 0.212 |
| MBW3 (g) | 461±10 | 455±5 | 454±25 | 448±14 | 0.770 | 457±12 | 482±15 | 461±16 | 459±8 | 0.164 |
| final bw (g) | 680±45 | 693±2 | 697±47 | 694±19 | 0.929 | 702±23 | 730±30 | 710±27 | 708±10 | 0.524 |
| total SGR | 1.38±0.08 | 1.41±0.00 | 1.41±0.08 | 1.41±0.04 | 0.873 | 1.43±0.04 | 1.47±0.05 | 1.44±0.04 | 1.43±0.02 | 0.611 |
| TGC1 | 3.14±0.17 | 3.20±0.02 | 3.08±0.31 | 3.01±0.15 | 0.657 | 3.31±0.07 | 3.42±0.12 | 3.23±0.17 | 3.19±0.10 | 0.180 |
| TGC2 | 5.16±0.13 | 4.97±0.40 | 4.85±0.37 | 5.29±0.66 | 0.618 | 5.35±0.34 | 5.40±0.57 | 5.34±0.50 | 4.72±0.25 | 0.254 |
| **TGC3** | **6.40^ab^±0.23** | **5.79^a^± 0.34** | **6.54^b^± 0.09** | **5.79^a^± 0.50** | **0.040** | **4.61^A^± 1.32** | **6.42^B^± 0.66** | **5.60^AB^± 0.48** | **6.39^B^± 0.45** | **0.077** |
| TGC4 | 4.18±0.70 | 4.54±0.15 | 4.63±0.24 | 4.72±0.00 | 0.385 | 4.65±0.12 | 4.58±0.24 | 4.69±0.12 | 4.71±0.04 | 0.724 |
| total TGC | 3.85±0.27 | 3.95±0.01 | 3.96±0.26 | 3.94±0.13 | 0.894 | 4.00±0.14 | 4.15±0.17 | 4.04±0.13 | 4.02±0.07 | 0.578 |
| total feed DM per FISH | 270±9 | 268±16 | 265±25 | 261±9 | 0.909 | 273±13 | 285±18 | 272±13 | 274±7 | 0.620 |
| FCR1 | 0.83±0.02 | 0.83±0.02 | 0.82±0.02 | 0.84±0.03 | 0.755 | 0.80±0.05 | 0.82±0.01 | 0.81±0.02 | 0.83±0.01 | 0.568 |
| FCR2 | 0.59±0.03 | 0.62±0.07 | 0.58±0.03 | 0.55±0.07 | 0.498 | 0.55±0.01 | 0.60±0.10 | 0.56±0.05 | 0.63±0.03 | 0.374 |
| FCR3 | 0.44± 0.01 | 0.46± 0.02 | 0.43± 0.04 | 0.46± 0.03 | 0.507 | 0.66± 0.20 | 0.48± 0.04 | 0.49± 0.03 | 0.44± 0.03 | 0.118 |
| **FCR4** | 0.56±0.12 | 0.48±0.07 | 0.49±0.01 | 0.48±0.01 | 0.415 | **0.48^AB^± 0.01** | **0.51^B^± 0.02** | **0.48^A^± 0.01** | **0.49 ^AB^± 0.01** | **0.076** |
| total FCR DM | 0.58±0.05 | 0.56±0.03 | 0.55±0.00 | 0.55±0.01 | 0.475 | 0.56±0.00 | 0.56±0.01 | 0.55±0.00 | 0.56±0.01 | 0.135 |
| total FE | 1.58±0.14 | 1.66±0.10 | 1.68±0.01 | 1.68±0.02 | 0.493 | 1.65±0.01 | 1.65±0.02 | 1.68±0.01 | 1.66±0.03 | 0.128 |

*Values in the same row with different small letter are significantly different (p<0.05), whereas those with different capital letter show tendency for difference (p<0.1) following Duncan post hoc test

Supplementary-Table 5 Apparent digestibility coefficient of dietary protein, lipid, and energy, as well as ADC of P and tested minerals Cu, Fe, Zn, Se, Mn as well as their levels in the faeces of Atlantic salmon fed diets (8 treatments (T)) with variable source (S) and levels (L) of trace minerals^*^. Values with significant differences or trends are marked bold.

| **%** | **IM1** | **IM2** | **IM3** | **IM4** | **OM1** | **OM2** | **OM3** | **OM4** | **p-values*** | | | |
| --- | --- | --- | --- | --- | --- | --- | --- | --- | --- | --- | --- | --- |
|  |  |  |  |  |  |  |  |  | **T** | **S** | **L** | **S x L** |
| ADC Protein | 90.9±0.6 | 90.6±0.2 | 90.8±1.0 | 89.6±0.6 | 90.5±0.5 | 90.0±0.3 | 90.3±1.0 | 90.7±0.3 | 0.239 | 0.696 | 0.419 | 0.103 |
| ADC Fat | 96.3±0.7 | 97.4±0.3 | 97.1±0.1 | 96.9±0.6 | 96.9±0.3 | 96.8±0.7 | 96.6±0.2 | 96.5±0.4 | 0.185 | 0.290 | 0.250 | 0.153 |
| ADC Energy | 86.3±0.8 | 86.2±0.2 | 85.9±1.2 | 84.8±0.9 | 86.2±0.3 | 85.0±0.5 | 85.5±1.2 | 85.5±0.7 | 0.217 | 0.452 | 0.149 | 0.275 |
| **ADC P** | **55.7±1.4** | **51.4±2.4** | **44.9±8.9** | **42.5±3.3** | **50.8±7.6** | **48.4±2.5** | **52.1±1.4** | **50.0±5.2** | 0.108 | 0.905 | 0.207 | **0.055** |
| **ADC Cu** | **32.0±6.0^c^** | **17.3±2.5^ab^** | **23.8±2.6^bc^** | **13.5±8.9^a^** | **31.0±0.1^c^** | **16.6±2.0^ab^** | **16.3±6.5^ab^** | **12.1±6.5^a^** | **0.001** | 0.232 | **0.000** | 0.631 |
| **ADC Fe** | **12.6±3.2^b^** | **2.4±2.1^ab^** | **-3.9±3.5^a^** | **-3.9±7.1^a^** | **4.9±6.3^ab^** | **-3.0±2.0^a^** | **4.9±10.0^ab^** | **0.1±6.4^a^** | **0.033** | 0.977 | **0.023** | **0.075** |
| **ADC Zn** | **36.9±3.0^d^** | **27.5±0.1^cd^** | **24.2±11.6^bc^** | **11.3±7.9^a^** | **31.5±1.5^cd^** | **20.8±0.1^abc^** | **14.3±9.0^ab^** | **9.8±3.8^a^** | **0.000** | **0.033^Ι^** | **0.000** | 0.700 |
| **ADC Se** | **42.7±6.7^a^** | **56.4±4.6^b^** | **60.8±3.4^b^** | **56.2±3.1^b^** | **63.4±1.9^b^** | **62.6±3.0^b^** | **44.8±7.3^a^** | **47.0±4.9^a^** | **0.000** | 0.825 | **0.044** | **0.000** |
| **ADC Mn** | **54.2±8.8^b^** | **32.4±2.1^a^** | **11.7±5.2^a^** | **12.4±7.8^a^** | **14.6±4.6^a^** | **18.3±6.0^a^** | **10.2±3.9^a^** | **11.0±9.8^a^** | **0.006** | 0.189 | **0.002** | 0.102 |
| **Mineral level in the faeces** | | | | | | | | | | | | |
| **Cu (mg/kg)** | **25.7^a^** | **43.0^b^** | **56.7^c^** | **76.0^e^** | **27.7^a^** | **43.3^b^** | **61.0^d^** | **79.7^e^** | **0.001** | 0.753 | **0.018** | 0.490 |
| **Fe (mg/kg)** | **1066.7^a^** | **1366.7^b^** | **1666.7^cd^** | **1866.7^d^** | **1066.7^a^** | **1300.0^b^** | **1566.7^c^** | **1700.0^d^** | **0.001** | 0.494 | **0.011** | 0.267 |
| **Zn (mg/kg)** | **200.0^a^** | **290.0^b^** | **383.3^c^** | **520.0^e^** | **233.3^a^** | **323.3^b^** | **460.0^d^** | **580.0^f^** | **0.001** | 0.356 | **0.001** | 0.195 |
| **Se (mg/kg)** | **1.6^abc^** | **1.6^ab^** | **1.8^cde^** | **1.8^bcd^** | **1.5^a^** | **1.7^abc^** | **1.9^cd^** | **2.0^d^** | **0.001** | 0.475 | 0.219 | **0.047** |
| **Mn (mg/kg)** | **156.7^a^** | **233.3^b^** | **273.3^c^** | **310.0^d^** | **180.0^a^** | **236.7^b^** | **283.3^c^** | **296.7^cd^** | **0.001** | 0.798 | 0.327 | 0.199 |
| Total P (%) | 2.2 | 2.3 | 2.6 | 2.5 | 2.2 | 2.4 | 2.2 | 2.3 | 0.256 | 0.194 | 0.180 | 0.299 |

* Values in the same row with different small letter are significantly different (p<0.05) following Duncan post hoc test

^Ι^ΙM>ΟM

Supplementary-Table 6 Blood serum metabolites in Atlantic salmon fed diets with added trace minerals in either inorganic (IM) or organic (OM) form at variable levels after handling stress (HS) and at final sampling (end)^*^ (values are means± standard variation; n=3 tanks). Values with significant differences or trends are marked bold.

|  | | **Sampling** | **IM1** | **IM2** | **IM3** | **IM4** | **OM1** | **OM2** | **OM3** | **OM4** | **p-value*** |
| --- | --- | --- | --- | --- | --- | --- | --- | --- | --- | --- | --- |
| **Cortisol (nmol/L)** | **HS 1** | **86±26^A^** | **88 ±24^A^** | **414 ±300^B^** | **197 ±73^AB^** | **174 ±62^A^** | **242 ±140^AB^** | **133 ±60^A^** | **157 ±30^A^** | **0.095** |  |
|  | HS 2 | 205 ±91 | 259 ±203 | 198 ±101 | 189 ±123 | 148 ±80 | 140 ±34 | 283 ±202 | 107 ±27 | 0.681 |  |
|  | HS 3 | 117 ±13 | 123 ±30 | 167 ±84 | 81 ±31 | 143 ±139 | 217 ±107 | 315 ±203 | 84 ±40 | 0.162 |  |
|  | **end** | **114 ±71^A^** | **200 ±87^AB^** | **405 ±173^B^** | **209 ±167^AB^** | **287 ±33^AB^** | **111 ±41^A^** | **200 ±88^AB^** | **165 ±121^A^** | **0.077** |  |
| K (mmol/L) | HS1 | 0.95 ±0.44 | 0.86 ±0.41 | 0.73 ±0.44 | 0.53 ±0.03 | 1.29 ±0.62 | 0.58 ±0.19 | 0.82 ±0.28 | 0.60 ±0.10 | 0.274 |  |
|  | HS 2 | 2.14 ±1.62 | 1.10 ±0.43 | 1.06 ±0.76 | 0.80 ±0.15 | 2.21 ±1.52 | 0.81 ±0.24 | 1.76 ±1.26 | 1.36 ±1.15 | 0.539 |  |
|  | HS 3 | 0.98 ±0.77 | 0.69 ±0.19 | 0.71 ±0.40 | 0.53 ±0.03 | 0.68 ±0.04 | 0.57 ±0.15 | 0.58 ±0.17 | 0.52 ±0.04 | 0.724 |  |
|  | end | 0.82 ±0.27 | 1.50 ±0.70 | 0.77 ±0.26 | 0.71 ±0.11 | 0.84 ±0.31 | 1.26 ±0.77 | 0.67 ±0.19 | 1.04 ±0.91 | 0.508 |  |
| Glucose (mmol/L) | HS1 | 4.49 ±0.16 | 4.78 ±0.05 | 4.73 ±0.34 | 4.80 ±0.10 | 4.69 ±0.30 | 4.87 ±0.15 | 4.81 ±0.12 | 5.17 ±0.39 | 0.122 |  |
|  | HS 2 | 4.81 ±0.50 | 5.01 ±0.34 | 4.77 ±0.32 | 5.08 ±0.17 | 4.57 ±0.13 | 4.88 ±0.32 | 4.93 ±0.37 | 4.81 ±0.17 | 0.621 |  |
|  | HS 3 | 5.07 ±0.42 | 4.69 ±0.55 | 5.04 ±0.45 | 5.38 ±0.18 | 5.21 ±0.23 | 5.09 ±0.41 | 4.84 ±0.36 | 5.36 ±0.47 | 0.443 |  |
|  | end | 4.90 ±0.32 | 4.92 ±0.61 | 4.36 ±0.10 | 4.88 ±0.36 | 5.06 ±0.20 | 4.89 ±0.45 | 5.19 ±0.39 | 5.26 ±0.75 | 0.381 |  |
| Total Protein (g/L) | HS 1 | 45.0 ±2.7 | 45.2 ±0.8 | 44.1 ±2.5 | 45.5 ±4.2 | 46.5 ±2.8 | 44.6 ±4.5 | 44.5 ±3.1 | 48.9 ±5.1 | 0.745 |  |
|  | HS 2 | 46.3 ±5.6 | 46.2 ±1.1 | 44.7 ±2.7 | 49.3 ±3.9 | 48.8 ±4.9 | 51.5 ±5.7 | 52.6 ±5.0 | 50.1 ±2.1 | 0.310 |  |
|  | HS 3 | 51.4 ±4.5 | 49.2 ±3.7 | 51.9 ±4.1 | 54.1 ±2.5 | 52.2 ±3.4 | 51.0 ±1.6 | 54.1 ±6.2 | 55.7 ±1.2 | 0.502 |  |
|  | end | 55.1 ±7.3 | 51.8 ±4.1 | 55.5 ±6.7 | 49.7 ±8.3 | 52.0 ±4.7 | 48.0 ±3.9 | 56.1 ±3.4 | 55.3 ±13.0 | 0.789 |  |
| **Cholesterol (mmol/L)** | HS 1 | 19.4 ±1.3 | 19.2 ±3.4 | 20.1 ±1.5 | 20.9 ±0.5 | 18.6 ±1.1 | 20.0 ±0.4 | 20.6 ±0.9 | 21.1 ±0.9 | 0.503 |  |
|  | HS 2 | 20.0 ±2.9 | 19.1 ±1.2 | 20.9 ±1.8 | 19.6 ±1.8 | 20.8 ±1.1 | 20.3 ±2.2 | 21.8 ±2.9 | 21.5 ±1.1 | 0.694 |  |
|  | **HS 3** | **22.1 ±0.5^ABC^** | **20.2 ±1.3^A^** | **21.9 ±1.5^ABC^** | **22.3 ±0.5^BC^** | **20.8 ±0.2^A^** | **21.3 ±0.5 ^ABC^** | **22.4 ±1.6^BC^** | **23.0 ±1.0^C^** | **0.067** |  |
|  | end | 22.9 ±0.8 | 22.8 ±0.5 | 23.4 ±0.6 | 21.9 ±1.9 | 23.0 ±0.7 | 23.1 ±0.4 | 23.8 ±0.7 | 23.1 ±2.0 | 0.671 |  |
| ASAT (U/L) | HS 1 | 681 ±150 | 1528 ±1344 | 1085 ±527 | 1064 ±356 | 1925 ±981 | 1262 ±448 | 872 ±305 | 1389 ±563 | 0.485 |  |
|  | HS 2 | 833 ±600 | 1638 ±1029 | 753 ±273 | 1293 ±903 | 1739 ±1415 | 2502 ±3074 | 1203 ±153 | 1233 ±401 | 0.791 |  |
|  | HS 3 | 757 ±381 | 906 ±354 | 845 ±476 | 617 ±60 | 1312 ±344 | 866 ±470 | 1186 ±652 | 870 ±329 | 0.540 |  |
|  | end | 848 ±253 | 755 ±171 | 790 ±241 | 510 ±192 | 760 ±256 | 808 ±177 | 560 ±141 | 564 ±187 | 0.340 |  |
| ALAT (U/L) | HS 1 | 6.0 ±1.9 | 17.4 ±18.1 | 9.3 ±4.3 | 9.1 ±3.6 | 17.8 ±8.0 | 11.2 ±2.6 | 7.3 ±2.8 | 17.4 ±14.3 | 0.543 |  |
|  | HS 2 | 6.1 ±1.7 | 10.0 ±5.9 | 7.1 ±1.6 | 8.2 ±5.8 | 13.2 ±8.1 | 19.9 ±18.0 | 9.2 ±2.7 | 7.7 ±3.2 | 0.468 |  |
|  | HS 3 | 7.8 ±4.2 | 6.1 ±1.7 | 6.1 ±2.5 | 8.6 ±4.5 | 11.1 ±1.9 | 6.7 ±3.6 | 9.1 ±3.5 | 7.0 ±3.1 | 0.563 |  |
|  | end | 8.6 ±3.0 | 9.1 ±2.9 | 8.1 ±1.4 | 4.9 ±3.9 | 8.6 ±1.9 | 8.8 ±3.0 | 5.7 ±1.8 | 5.7 ±3.0 | 0.370 |  |
| CK (U/L) | HS 1 | 28272 ±21792 | 172753 ±263445 | 70031 ±62185 | 61316 ±62797 | 236405 ±209799 | 66375 ±63476 | 43241 ±21164 | 168939 ±160679 | 0.524 |  |
|  | HS 2 | 49689 ±59874 | 130462 ±100306 | 23026 ±13164 | 131539 ±115902 | 175920 ±220604 | 168324 ±220547 | 63546 ±43399 | 80486 ±27935 | 0.743 |  |
|  | HS 3 | 28309 ±24861 | 49368 ±29298 | 19258 ±11553 | 17961 ±3478 | 75270 ±42511 | 38657 ±38087 | 44149 ±24689 | 46383 ±33436 | 0.326 |  |
|  | end | 23357 ±5803 | 13958 ±4041 | 17822 ±6116 | 16653 ±11278 | 15025 ±5660 | 17849 ±11874 | 16058 ±7417 | 19372 ±9781 | 0.899 |  |

* Values in the same row with different small letter are significantly different (p<0.05), whereas those with different capital letter show tendency for difference (p<0.1) following Duncan post hoc test

Supplementary-Table 7 Trace mineral retention efficiency (% RE) and tissue mineralisation (given in mg/kg and are means ±standard deviation, N=3 tanks) in Atlantic salmon fed diets (8 treatments (T)) with variable source (S) and levels (L) of trace minerals^*^.

|  | |  | | | | | | | | **p-values*** | | | |
| --- | --- | --- | --- | --- | --- | --- | --- | --- | --- | --- | --- | --- | --- |
|  |  | **IM1** | **IM2** | **IM3** | **IM4** | **OM1** | **OM2** | **OM3** | **OM4** | **T** | **S** | **L** | **S x L** |
| **% RE** | |  |  |  |  |  |  |  |  |  |  |  |  |
|  | **Cu** | **23.39 ±4.32^cd^** | **19.48 ±3.51^bc^** | **12.16 ±2.99^a^** | **8.13 ±1.67^a^** | **24.36 ±2.37^d^** | **18.32 ±0.21^b^** | **11.21 ±0.58^a^** | **7.44 ±1.8^a^** | **0.000** | 0.672 | **0.000; 1>2>3≥4** | 0.887 |
|  | **Fe** | **6.06 ±1.33^c^** | **4.86 ±0.45^abc^** | **4.97 ±0.26^abc^** | **3.51 ±0.20^a^** | **7.81 ±1.25^d^** | **5.41 ±0.39^bc^** | **3.98 ±0.26^ab^** | **4.01 ±0.98^ab^** | **0.000** | 0.172 | **0.000**  **1≥3,2≥4 ^I^**  **1>2≥3=4 ^⭘^** | **0.057** |
|  | **Mn** | **4.73 ±0.87^b^** | **3.50 ±0.27^a^** | **3.45 ±0.57^a^** | **3.01 ±0.30^a^** | **5.65 ±1.12^b^** | **3.28 ±0.06^a^** | **3.31 ±0.71^a^** | **2.68 ±0.69^a^** | **0.001** | 0.878 | **0.000 1>2,3,4** | 0.378 |
|  | **Se** | **27.03 ±12.16^abc^** | **13.17 ±9.04^a^** | **30.59 ±8.77^abc^** | **19.36 ±8.50^a^** | **42.89 ±1.22^c^** | **38.84 ±13.98^bc^** | **21.20 ±1.01^ab^** | **26.15 ±13.78^abc^** | **0.030** | **0.027 OM>IM** | 0.200 | **0.041** |
|  | **Zn** | **20.55 ±7.50^ab^** | **28.92 ±0.86^b^** | **25.86 ±5.25^ab^** | **18.71 ±4.36^a^** | **29.29 ±0.35^b^** | **27.91 ±1.30^b^** | **21.82 ±1.70^ab^** | **18.34 ±8.66^a^** | **0.044** | 0.670 | **0.020**  **2≥3≥1≥4^IM^ 1,2≥3≥4^OM^** | 0.157 |
| **Mineralisation** | | |  |  |  |  |  |  |  |  |  |  |  |
| **Gills** | **Cu** | 0.961^ab^ ±0.354 | 0.948 ^ab^ ±0.233 | 0.815^a^ ±0.073 | 1.046 ^ab^ ±0.193 | 1.158^ab^ ±0.433 | 1.779^c^ ±0.064 | 0.678^a^ ±0.162 | 1.54^bc^ ±0.659 | **0.013** | **0.021^⭘^** | **0.022**  **3<2, 4** | 0.112 |
|  | Fe | 23.5±3.5 | 29.9±10.8 | 23.6±3 | 20.2±1 | 25.1±3.5 | 26.1±3.8 | 25.7±2 | 34.7±20.9 | 0.613 | 0.325 | 0.836 | 0.344 |
|  | **Mn** | 6.73±1.08 | 6.88±1.11 | 6.86±0.73 | 6.42±1.03 | 6.88±0.48 | 7.22±0.95 | 5.3±1.18 | 9.42±2.92 | 0.102 | 0.397 | 0.180 | **0.071** |
|  | Se | 0.233±0.036 | 0.254±0.043 | 0.215±0.046 | 0.22±0.018 | 0.206±0.029 | 0.252±0.062 | 0.235±0.016 | 0.379±0.187 | 0.204 | 0.243 | 0.274 | 0.180 |
|  | **Zn** | 72^a^ ±9.6 | 89.4^a^ ±11.7 | 90.2^a^ ±12 | 90.3^a^ ±14 | 71.2^a^ ±2.4 | 99.8^a^ ±3.8 | 87.4^a^ ±9 | 144^b^ ±49 | **0.010** | **0.079^⭘^** | **0.009**  **4>1** | **0.082** |
|  | K | 650±177 | 830±144 | 683±58 | 611±85 | 715±204 | 771±209 | 711±156 | 828±287 | 0.747 | 0.401 | 0.671 | 0.608 |
|  | **Mg** | 526±92 | 541±90 | 547±58 | 475±13 | 521±17 | 592±24 | 452±61 | 715±263 | 0.188 | 0.298 | 0.449 | **0.090** |
|  | Na | 866±204 | 1087±137 | 964±283 | 792±64 | 899±159 | 935±218 | 860±160 | 1086±411 | 0.704 | 0.850 | 0.787 | 0.354 |
|  | **Ca** | 9070^AB^ ±1289 | 9359^ABC^ ±1076 | 9599^ABC^ ±1244 | 8859^AB^ ±1025 | 8890^AB^ ±619 | 11213^BC^ ±775 | 7570^A^ ±1215 | 12613^C^ ±4210 | **0.079** | 0.262 | 0.158 | **0.065** |
|  | **Y** | 0.045±0.003 | 0.044±0.012 | 0.036±0.009 | 0.039±0.026 | 0.071±0.034 | 0.056±0.025 | 0.062±0.018 | 0.094±0.061 | 0.290 | **0.023^⭘^** | 0.692 | 0.645 |
| **NQC** | Cu | 0.321±0.13 | 0.385±0.097 | 0.297±0.122 | 0.295±0.075 | 0.225±0.058 | 0.237±0.056 | 0.371±0.125 | 0.278±0.068 | 0.427 | 0.248 | 0.703 | 0.243 |
|  | Fe | 2.456±1.065 | 2.664±0.616 | 2.132±0.652 | 2.222±0.651 | 1.802±0.558 | 1.901±0.536 | 2.278±0.76 | 3.043±2.087 | 0.822 | 0.785 | 0.824 | 0.493 |
|  | Mn | 0.124±0.043 | 0.14±0.051 | 0.115±0.028 | 0.106±0.024 | 0.107±0.037 | 0.133±0.005 | 0.1±0.034 | 0.121±0.045 | 0.862 | 0.699 | 0.549 | 0.862 |
|  | Se | 0.145±0.014 | 0.173±0.021 | 0.129±0.011 | 0.125±0.035 | 0.125±0.05 | 0.146±0.033 | 0.179±0.051 | 0.16±0.033 | 0.377 | 0.510 | 0.598 | 0.168 |
|  | Zn | 4.213±1.081 | 4.765±1.412 | 4.308±0.568 | 3.684±1.332 | 3.497±1.262 | 3.931±0.797 | 4.1±1.393 | 4.47±1.272 | 0.906 | 0.619 | 0.902 | 0.629 |
| **nqc** | K | 5007±936 | 6069±1512 | 4881±842 | 4383±1151 | 4121±988 | 4363±845 | 5279±1864 | 4859±796 | 0.573 | 0.382 | 0.711 | 0.336 |
|  | Mg | 378±84 | 447±119 | 370±63 | 330±97 | 307±82 | 328±63 | 381±131 | 364±66 | 0.691 | 0.344 | 0.795 | 0.456 |
|  | Na | 544±183 | 642±179 | 506±96 | 509±177 | 406±125 | 434±91 | 498±176 | 518±110 | 0.668 | 0.168 | 0.901 | 0.533 |
|  | Ca | 82±41 | 122±87 | 84±30 | 70±31 | 80±36 | 88±41 | 66±22 | 85±45 | 0.883 | 0.602 | 0.663 | 0.809 |
|  | Y | 0.007±0.008 | 0.001±0.002 | 0.016±0.014 | 0.006±0.005 | 0.003±0.004 | 0.006±0.008 | 0.002±0.002 | 0.013±0.012 | 0.327 | 0.709 | 0.532 | 0.135 |
| **LIVER** | **Cu** | 153±58 | 192±67 | 127±51 | 113±35 | 111±37 | 179±43 | 124±9 | 102±4 | 0.161 | 0.338 | **0.037**  **2>4** | 0.871 |
|  | Fe | 176±71 | 201±59 | 144±49 | 134±37 | 122±31 | 184±33 | 144±12 | 128±4 | 0.278 | 0.298 | 0.111 | 0.695 |
|  | Mn | 2.85±1.04 | 3.12±0.71 | 2.44±0.66 | 2.17±0.62 | 2±0.56 | 2.73±0.64 | 2.03±0.26 | 2.1±0.25 | 0.297 | 0.117 | 0.185 | 0.766 |
|  | Se | 4.49±1.69 | 5.53±1.64 | 3.79±1.3 | 3.81±1.26 | 3.07±0.96 | 4.63±0.84 | 3.53±0.45 | 3.25±0.1 | 0.232 | 0.115 | 0.113 | 0.841 |
|  | Zn | 31.9±11.7 | 37±11.5 | 31.6±12.8 | 26.5±6.6 | 22.6±6.5 | 36.4±7.3 | 26.3±3.2 | 28±5.8 | 0.462 | 0.357 | 0.230 | 0.715 |
|  | K | 6423±2303 | 7119±2208 | 5391±2092 | 4667±1166 | 4530±1424 | 6791±1473 | 4827±714 | 4672±401 | 0.310 | 0.307 | 0.120 | 0.755 |
|  | Mg | 410±152 | 458±123 | 331±97 | 306±94 | 298±96 | 433±106 | 302±37 | 309±25 | 0.292 | 0.328 | 0.103 | 0.772 |
|  | Na | 1882±690 | 2215±738 | 1560±551 | 1543±448 | 1510±715 | 2143±713 | 1488±95 | 1468±55 | 0.533 | 0.531 | 0.179 | 0.956 |
|  | Ca | 122±48 | 187±97 | 122±17 | 142±68 | 106±36 | 146±63 | 83±5 | 106±20 | 0.417 | 0.143 | 0.213 | 0.975 |
|  | Y | 0±0 | 0.119±0.068 | 0±0 | 0.076±0.132 | 0.048±0.042 | 0.071±0.072 | 0.018±0.032 | 0.116±0.128 | 0.366 | 0.645 | 0.128 | 0.698 |
| **s pleen** | Cu | 2.15±1.31 | 2.34±2.2 | 2.05±1.79 | 3.47±1.35 | 1.95±0.89 | 2.45±2.04 | 4.2±0.17 | 2.59±0.86 | 0.558 | 0.630 | 0.544 | 0.348 |
|  | Fe | 178±47 | 159±31 | 166±71 | 148±33 | 152±24 | 152±46 | 141±28 | 151±36 | 0.971 | 0.429 | 0.931 | 0.912 |
|  | Mn | 0.92±0.32 | 1.02±0.34 | 1.03±0.51 | 1.52±0.32 | 1.01±0.34 | 1.22±0.36 | 1.34±0.09 | 1.31±0.16 | 0.347 | 0.486 | 0.158 | 0.561 |
|  | **Se** | 0.457±0.023 | 0.45±0.055 | 0.469±0.044 | 0.516±0.056 | 0.442±0.034 | 0.59±0.135 | 0.512±0.016 | 0.574±0.117 | 0.144 | **0.075^⭘^** | 0.162 | 0.351 |
|  | **Zn** | 51.5^a^ ±21.5 | 73.6^abc^ ±15.3 | 59.5^ab^ ±23.7 | 91^bc^ ±14.7 | 64.2^ab^ ±17.3 | 83^abc^ ±23.9 | 106.1^c^ ±13.8 | 100.5^c^ ±9.6 | **0.016** | **0.018^⭘^** | **0.018 4>1** | 0.254 |
|  | K | 3666±609 | 3064±757 | 3188±804 | 3436±602 | 3484±989 | 3688±799 | 3890±555 | 3321±320 | 0.852 | 0.385 | 0.939 | 0.584 |
|  | Mg | 458±129 | 468±58 | 402±118 | 493±55 | 480±138 | 489±106 | 552±48 | 468±89 | 0.790 | 0.314 | 0.997 | 0.467 |
|  | Na | 1519±90 | 1355±34 | 1386±170 | 1395±116 | 1423±83 | 1508±44 | 1438±125 | 1404±82 | 0.480 | 0.486 | 0.647 | 0.241 |
|  | Ca | 208±112 | 186±41 | 192±83 | 224±44 | 185±72 | 258±131 | 203±25 | 199±58 | 0.949 | 0.786 | 0.932 | 0.687 |
|  | Y | 0.033±0.02 | 0.017±0.012 | 0.039±0.014 | 0.018±0.001 | 0.015±0.007 | 0.06±0.075 | 0.018±0.004 | 0.058±0.034 | 0.421 | 0.401 | 0.815 | 0.162 |
| **KIDNEY** | Cu | 3.56±2.65 | 2.86±1.06 | 2.3±1.21 | 2.98±1.89 | 4.64±1.16 | 3.74±2.38 | 3.54±2.04 | 2.6±0.87 | 0.799 | 0.346 | 0.588 | 0.852 |
|  | Fe | 125±24 | 128±19 | 123±36 | 119±31 | 125±8 | 108±15 | 122±24 | 102±8 | 0.844 | 0.328 | 0.717 | 0.829 |
|  | Mn | 0.64±0.079 | 0.57±0.067 | 0.721±0.138 | 0.705±0.07 | 0.644±0.087 | 0.696±0.131 | 0.859±0.229 | 0.706±0.226 | 0.449 | 0.264 | 0.243 | 0.745 |
|  | Se | 0.446±0.142 | 0.574±0.051 | 0.551±0.079 | 0.522±0.111 | 0.552±0.033 | 0.524±0.058 | 0.455±0.104 | 0.455±0.017 | 0.414 | 0.448 | 0.623 | 0.199 |
|  | **Zn** | **22.1^ab^ ±0.7** | **25.9^bc^ ±2.4** | **25.8^bc^ ±2.8** | **26.1^bc^ ±0.8** | **21^a^ ±0.7** | **26.5^bc^ ±2.3** | **27.6^c^ ±2.7** | **25.3^bc^ ±3.8** | **0.037** | 0.894 | **0.005**  **1<2,3,4^IM^**  **1<2,3,4^OM^** | 0.688 |
|  | **K** | **4291±215** | **4265±127** | **4227±173** | **4559±431** | **4191±212** | **4309±321** | **3887±398** | **4036±84** | 0.207 | **0.055^I^** | 0.412 | 0.314 |
|  | Mg | 310±40 | 292±23 | 305±51 | 327±30 | 303±57 | 318±26 | 321±44 | 297±25 | 0.944 | 0.927 | 0.980 | 0.604 |
|  | Na | 1636±54 | 1572±100 | 1601±32 | 1543±108 | 1563±63 | 1619±166 | 1574±68 | 1442±76 | 0.329 | 0.318 | 0.179 | 0.544 |
|  | Ca | 258±105 | 182±67 | 303±127 | 285±31 | 250±68 | 294±154 | 383±55 | 293±78 | 0.408 | 0.227 | 0.260 | 0.640 |
|  | Y | 0.015±0.011 | 0.013±0.014 | 0.092±0.07 | 0.035±0.034 | 0.065±0.083 | 0.044±0.052 | 0.007±0.011 | 0.108±0.09 | 0.267 | 0.452 | 0.587 | 0.102 |
| **Skin** | Cu | 0.647±0.095 | 0.823±0.239 | 0.61±0.017 | 0.677±0.025 | 0.673±0.105 | 0.783±0.158 | 0.647±0.06 | 0.78±0.157 | 0.391 | 0.738 | 0.102 | 0.766 |
|  | Fe | 7.03±0.22 | 8.2±1.63 | 6.92±0.59 | 9.22±2.18 | 10.35±3.48 | 8.58±2.24 | 8.01±2.01 | 7.17±0.62 | 0.389 | 0.370 | 0.694 | 0.139 |
|  | Mn | 22.7 ±2.1 | 27.9 ±7.0 | 34.1 ±3.9 | 35.6 ±3.2 | 20.9 ±1.6 | 32.1±6.8 | 38.9 ±2 | 38.4 ±3.7 | 0.833 | 0.257 | 0.360 | 0.346 |
|  | **Se** | **0.24±0.03** | **0.343±0.121** | **0.357±0.099** | **0.217±0.04** | **0.21±0.046** | **0.307±0.031** | **0.353±0.167** | **0.243±0.085** | 0.255 | 0.883 | **0.085** | 0.948 |
|  | **Zn** | **5.14^a^ ±0.19** | **4.67^ab^ ±1.19** | **5.31^bc^±0.56** | **4.65^bc^±0.75** | **5.34^a^±1.17** | **4.96^bc^±0.57** | **4.8^c^±0.14** | **5.37^c^±0.65** | **0.000** | 0.155 | **0.000** | 0.724 |
| **WB** | **Cu** | **1.42±0.09** | **1.53±0.25** | **1.42±0.24** | **1.27±0.17** | **1.49±0.11** | **1.54±0.02** | **1.34±0.04** | **1.21±0.18** | 0.162 | 0.809 | **0.031**  **4<2** | 0.831 |
|  | **Fe** | 10.08±1.27 | 9.29±0.9 | 10.57±0.37 | 9.24±0.38 | 11.36±1.47 | 9.93±0.61 | 9.24±0.42 | 9.73±1.8 | 0.222 | 0.533 | 0.202 | 0.197 |
|  | **Mn** | 1.45±0.21 | 1.23±0.04 | 1.41±0.19 | 1.49±0.13 | 1.58±0.27 | 1.25±0.02 | 1.41±0.25 | 1.35±0.27 | 0.420 | 0.992 | 0.148 | 0.704 |
|  | **Se** | **0.193^ABC^ ±0.04** | **0.143^A^ ±0.035** | **0.213^ABC^ ±0.035** | **0.173^AB^ ±0.035** | **0.247^C^±0.006** | **0.237^BC^ ±0.051** | **0.173^AB^ ±0.006** | **0.2^ABC^ ±0.056** | **0.056** | **0.044^⭘^** | 0.417 | **0.044** |
|  | **Zn** | **17.2±1.6** | **22.1±0.6** | **24±2.9** | **22.4±2.9** | **20.4±0.3** | **22.7±0.4** | **22.5±1** | **23±6** | 0.148 | 0.506 | **0.044**  **1<3^IM^** | 0.531 |

* Values in the same row with different small letter are significantly different (p<0.05), whereas those with different capital letter show tendency for difference (p<0.1) following Duncan post hoc test

^⭘^OM>IM

^I^IM>OM

Supplementary-Table 8 Tissue mineralisation in Atlantic salmon when fed diets with added trace minerals in either inorganic (IM) or organic (OM) form at variable levels ^*^(values are means± standard variation; n=3 tanks). Values with significant differences or trends are marked bold.

|  |  | **IM1** | **IM2** | **IM3** | **IM4** | **p-value*** | **OM1** | **OM2** | **OM3** | **OM4** | **p-value*** |
| --- | --- | --- | --- | --- | --- | --- | --- | --- | --- | --- | --- |
| **Gills** | **Cu** | 0.961±0.354 | 0.948±0.233 | 0.815 ±0.073 | 1.046 ±0.193 | 0.696 | **1.158^AB^±0.433** | **1.779^B^±0.064** | **0.678^A^ ±0.162** | **1.54^B^ ±0.659** | **0.069** |
|  | Fe | 23.5±3.5 | 29.9±10.8 | 23.6±3 | 20.2±1 | 0.305 | 25.1±3.5 | 26.1±3.8 | 25.7±2 | 34.7±20.9 | 0.748 |
|  | Mn | 6.73±1.08 | 6.88±1.11 | 6.86±0.73 | 6.42±1.03 | 0.936 | 6.88±0.48 | 7.22±0.95 | 5.3±1.18 | 9.42±2.92 | 0.119 |
|  | Se | 0.233±0.036 | 0.254±0.043 | 0.215±0.046 | 0.22±0.018 | 0.595 | 0.206±0.029 | 0.252±0.062 | 0.235±0.016 | 0.379±0.187 | 0.288 |
|  | **Zn** | 72 ±9.6 | 89.4 ±11.7 | 90.2 ±12 | 90.3 ±14 | 0.244 | **71.2^A^ ±2.4** | **99.8^AB^ ±3.8** | **87.4^A^ ±9** | **144^B^ ±49** | **0.074** |
|  | K | 650±177 | 830±144 | 683±58 | 611±85 | 0.232 | 715±204 | 771±209 | 711±156 | 828±287 | 0.934 |
|  | Mg | 526±92 | 541±90 | 547±58 | 475±13 | 0.608 | 521±17 | 592±24 | 452±61 | 715±263 | 0.236 |
|  | Na | 866±204 | 1087±137 | 964±283 | 792±64 | 0.324 | 899±159 | 935±218 | 860±160 | 1086±411 | 0.775 |
|  | Ca | 9070 ±1289 | 9359 ±1076 | 9599 ±1244 | 8859 ±1025 | 0.871 | 8890 ±619 | 11213 ±775 | 7570 ±1215 | 12613 ±4210 | 0.124 |
|  | Y | 0.045±0.003 | 0.044±0.012 | 0.036±0.009 | 0.039±0.026 | 0.873 | 0.071±0.034 | 0.056±0.025 | 0.062±0.018 | 0.094±0.061 | 0.604 |
| **NQC** | Cu | 0.321±0.13 | 0.385±0.097 | 0.297±0.122 | 0.295±0.075 | 0.720 | 0.225±0.058 | 0.237±0.056 | 0.371±0.125 | 0.278±0.068 | 0.200 |
|  | Fe | 2.456±1.065 | 2.664±0.616 | 2.132±0.652 | 2.222±0.651 | 0.830 | 1.802±0.558 | 1.901±0.536 | 2.278±0.76 | 3.043±2.087 | 0.610 |
|  | Mn | 0.124±0.043 | 0.14±0.051 | 0.115±0.028 | 0.106±0.024 | 0.723 | 0.107±0.037 | 0.133±0.005 | 0.1±0.034 | 0.121±0.045 | 0.629 |
|  | Se | 0.145±0.014 | 0.173±0.021 | 0.129±0.011 | 0.125±0.035 | 0.106 | 0.125±0.05 | 0.146±0.033 | 0.179±0.051 | 0.16±0.033 | 0.504 |
|  | Zn | 4.213±1.081 | 4.765±1.412 | 4.308±0.568 | 3.684±1.332 | 0.725 | 3.497±1.262 | 3.931±0.797 | 4.1±1.393 | 4.47±1.272 | 0.780 |
|  | K | 5007±936 | 6069±1512 | 4881±842 | 4383±1151 | 0.382 | 4121±988 | 4363±845 | 5279±1864 | 4859±796 | 0.629 |
|  | Mg | 378±84 | 447±119 | 370±63 | 330±97 | 0.519 | 307±82 | 328±63 | 381±131 | 364±66 | 0.674 |
|  | Na | 544±183 | 642±179 | 506±96 | 509±177 | 0.719 | 406±125 | 434±91 | 498±176 | 518±110 | 0.562 |
|  | Ca | 82±41 | 122±87 | 84±30 | 70±31 | 0.671 | 80±36 | 88±41 | 66±22 | 85±45 | 0.853 |
|  | Y | 0.007±0.008 | 0.001±0.002 | 0.016±0.014 | 0.006±0.005 | 0.256 | 0.003±0.004 | 0.006±0.008 | 0.002±0.002 | 0.013±0.012 | 0.339 |
| **LIVER** | **Cu** | 153±58 | 192±67 | 127±51 | 113±35 | 0.357 | **111^A^±37** | **179^B^±43** | **124^A^±9** | **102^A^±4** | **0.060** |
|  | **Fe** | 176±71 | 201±59 | 144±49 | 134±37 | 0.476 | **122^A^±31** | **184^B^±33** | **144^A^±12** | **128^A^±4** | **0.084** |
|  | Mn | 2.85±1.04 | 3.12±0.71 | 2.44±0.66 | 2.17±0.62 | 0.484 | 2±0.56 | 2.73±0.64 | 2.03±0.26 | 2.1±0.25 | 0.336 |
|  | Se | 4.49±1.69 | 5.53±1.64 | 3.79±1.3 | 3.81±1.26 | 0.478 | 3.07±0.96 | 4.63±0.84 | 3.53±0.45 | 3.25±0.1 | 0.125 |
|  | Zn | 31.9±11.7 | 37±11.5 | 31.6±12.8 | 26.5±6.6 | 0.715 | 22.6±6.5 | 36.4±7.3 | 26.3±3.2 | 28±5.8 | 0.189 |
|  | K | 6423±2303 | 7119±2208 | 5391±2092 | 4667±1166 | 0.487 | 4530±1424 | 6791±1473 | 4827±714 | 4672±401 | 0.147 |
|  | Mg | 410±152 | 458±123 | 331±97 | 306±94 | 0.421 | 298±96 | 433±106 | 302±37 | 309±25 | 0.210 |
|  | Na | 1882±690 | 2215±738 | 1560±551 | 1543±448 | 0.531 | 1510±715 | 2143±713 | 1488±95 | 1468±55 | 0.395 |
|  | Ca | 122±48 | 187±97 | 122±17 | 142±68 | 0.591 | 106±36 | 146±63 | 83±5 | 106±20 | 0.360 |
|  | Y | 0±0 | 0.119±0.068 | 0±0 | 0.076±0.132 | 0.211 | 0.048±0.042 | 0.071±0.072 | 0.018±0.032 | 0.116±0.128 | 0.557 |
| **s pleen** | Cu | 2.15±1.31 | 2.34±2.2 | 2.05±1.79 | 3.47±1.35 | 0.726 | 1.95±0.89 | 2.45±2.04 | 4.2±0.17 | 2.59±0.86 | 0.301 |
|  | Fe | 178±47 | 159±31 | 166±71 | 148±33 | 0.890 | 152±24 | 152±46 | 141±28 | 151±36 | 0.953 |
|  | Mn | 0.92±0.32 | 1.02±0.34 | 1.03±0.51 | 1.52±0.32 | 0.282 | 1.01±0.34 | 1.22±0.36 | 1.34±0.09 | 1.31±0.16 | 0.850 |
|  | Se | 0.457±0.023 | 0.45±0.055 | 0.469±0.044 | 0.516±0.056 | 0.359 | 0.442±0.034 | 0.59±0.135 | 0.512±0.016 | 0.574±0.117 | 0.460 |
|  | Zn | 51.5 ±21.5 | 73.6 ±15.3 | 59. ±23.7 | 91 ±14.7 | 0.138 | 64.2 ±17.3 | 83 ±23.9 | 106.1 ±13.8 | 100.5^c^ ±9.6 | 0.164 |
|  | K | 3666±609 | 3064±757 | 3188±804 | 3436±602 | 0.731 | 3484±989 | 3688±799 | 3890±555 | 3321±320 | 0.556 |
|  | Mg | 458±129 | 468±58 | 402±118 | 493±55 | 0.702 | 480±138 | 489±106 | 552±48 | 468±89 | 0.492 |
|  | Na | 1519±90 | 1355±34 | 1386±170 | 1395±116 | 0.367 | 1423±83 | 1508±44 | 1438±125 | 1404±82 | 0.511 |
|  | Ca | 208±112 | 186±41 | 192±83 | 224±44 | 0.922 | 185±72 | 258±131 | 203±25 | 199±58 | 0.799 |
|  | Y | 0.033±0.02 | 0.017±0.012 | 0.039±0.014 | 0.018±0.001 | 0.196 | 0.015±0.007 | 0.06±0.075 | 0.018±0.004 | 0.058±0.034 | 0.550 |
| **KIDNEY** | Cu | 3.56±2.65 | 2.86±1.06 | 2.3±1.21 | 2.98±1.89 | 0.865 | 4.64±1.16 | 3.74±2.38 | 3.54±2.04 | 2.6±0.87 | 0.511 |
|  | Fe | 125±24 | 128±19 | 123±36 | 119±31 | 0.985 | 125±8 | 108±15 | 122±24 | 102±8 | 0.444 |
|  | Mn | 0.64±0.079 | 0.57±0.067 | 0.721±0.138 | 0.705±0.07 | 0.255 | 0.644±0.087 | 0.696±0.131 | 0.859±0.229 | 0.706±0.226 | 0.671 |
|  | Se | 0.446±0.142 | 0.574±0.051 | 0.551±0.079 | 0.522±0.111 | 0.480 | 0.552±0.033 | 0.524±0.058 | 0.455±0.104 | 0.455±0.017 | 0.386 |
|  | **Zn** | **22.1^A^ ±0.7** | **25.9^B^ ±2.4** | **25.8^B^ ±2.8** | **26.1^B^ ±0.8** | **0.092** | 21 ±0.7 | 26.5 ±2.3 | 27.6 ±2.7 | 25.3 ±3.8 | 0.164 |
|  | K | 4291±215 | 4265±127 | 4227±173 | 4559±431 | 0.447 | 4191±212 | 4309±321 | 3887±398 | 4036±84 | 0.295 |
|  | Mg | 310±40 | 292±23 | 305±51 | 327±30 | 0.722 | 303±57 | 318±26 | 321±44 | 297±25 | 0.578 |
|  | Na | 1636±54 | 1572±100 | 1601±32 | 1543±108 | 0.562 | 1563±63 | 1619±166 | 1574±68 | 1442±76 | 0.263 |
|  | Ca | 258±105 | 182±67 | 303±127 | 285±31 | 0.423 | 250±68 | 294±154 | 383±55 | 293±78 | 0.616 |
|  | Y | 0.015±0.011 | 0.013±0.014 | 0.092±0.07 | 0.035±0.034 | 0.129 | 0.065±0.083 | 0.044±0.052 | 0.007±0.011 | 0.108±0.09 | 0.398 |
| **Skin** | Cu | 0.647±0.095 | 0.823±0.239 | 0.61±0.017 | 0.677±0.025 | 0.259 | 0.673±0.105 | 0.783±0.158 | 0.647±0.06 | 0.78±0.157 | 0.375 |
|  | Fe | 7.03±0.22 | 8.2±1.63 | 6.92±0.59 | 9.22±2.18 | 0.222 | 10.35±3.48 | 8.58±2.24 | 8.01±2.01 | 7.17±0.62 | 0.464 |
|  | **Mn** | **22.7^a^ ±2.1** | **27.9^ab^ ±7.0** | **34.1^b^ ±3.9** | **35.6^b^ ±3.2** | **0.026** | **20.9 ^a^±1.6** | **32.1^b^±6.8** | **38.9^b^ ±2** | **38.4^b^ ±3.7** | **0.011** |
|  | **Se** | 0.24±0.03 | 0.343±0.121 | 0.357±0.099 | 0.217±0.04 | 0.162 | 0.21±0.046 | 0.307±0.031 | 0.353±0.167 | 0.243±0.085 | 0.514 |
|  | Zn | 5.14 ±0.19 | 4.67 ±1.19 | 5.31±0.56 | 4.65±0.75 | 0.647 | 5.34±1.17 | 4.96±0.57 | 4.8±0.14 | 5.37±0.65 | 0.165 |
| **WB** | **Cu** | 1.42±0.09 | 1.53±0.25 | 1.42±0.24 | 1.27±0.17 | 0.484 | **1.49^ab^±0.11** | **1.54^b^±0.02** | **1.34^ab^±0.04** | **1.21^a^±0.18** | **0.030** |
|  | Fe | 10.08±1.27 | 9.29±0.9 | 10.57±0.37 | 9.24±0.38 | 0.218 | 11.36±1.47 | 9.93±0.61 | 9.24±0.42 | 9.73±1.8 | 0.618 |
|  | Mn | 1.45±0.21 | 1.23±0.04 | 1.41±0.19 | 1.49±0.13 | 0.260 | 1.58±0.27 | 1.25±0.02 | 1.41±0.25 | 1.35±0.27 | 0.511 |
|  | Se | 0.193 ±0.04 | 0.143 ±0.035 | 0.213 ±0.035 | 0.173 ±0.035 | 0.179 | 0.247±0.006 | 0.237 ±0.051 | 0.173 ±0.006 | 0.2 ±0.056 | 0.245 |
|  | **Zn** | **17.2^a^±1.6** | **22.1^ab^±0.6** | **24^b^±2.9** | **22.4^b^±2.9** | **0.028** | 20.4±0.3 | 22.7±0.4 | 22.5±1 | 23±6 | 0.827 |

* Values in the same row with different small letter are significantly different (p<0.05), whereas those with different capital letter show tendency for difference (p<0.1) following Duncan post hoc test

Supplementary-Table 9 Whole body (WB) (round/wet) total lipids (Bligh and Dyer extract) and fatty acid content (as % of whole body) of Atlantic salmon fed diets (8 treatments (T)) with variable source (S) and levels (L) of trace minerals* (values are means± standard variation; n=3 tanks)

|  | **IM1** | **IM2** | **IM3** | **IM4** | **OM1** | **OM2** | **OM3** | **OM4** | **p-values** | | | |
| --- | --- | --- | --- | --- | --- | --- | --- | --- | --- | --- | --- | --- |
|  |  |  |  |  |  |  |  |  | **T** | **S** | **L** | **S x L** |
| **WB B&D extract** | 14.33±0.33 | 14.78±0.20 | 14.72±0.55 | 14.18±0.38 | 15.10±0.27 | 14.63±1.06 | 14.17±0.13 | 14.29±0.44 | 0.297 | 0.835 | 0.314 | 0.185 |
| **14:0** | **0.39±0.01** | **0.37±0.01** | **0.38±0.03** | **0.38±0.01** | **0.39±0.01** | **0.40±0.03** | **0.40±0.01** | **0.40±0.04** | 0.514 | **0.036^⭘^** | 0.993 | 0.765 |
| **16:0** | 1.61±0.02 | 1.61±0.03 | 1.60±0.14 | 1.60±0.04 | 1.67±0.03 | 1.67±0.12 | 1.66±0.04 | 1.62±0.11 | 0.860 | 0.150 | 0.897 | 0.973 |
| **18:0** | 0.33±0.01 | 0.33±0.00 | 0.33±0.04 | 0.33±0.01 | 0.35±0.01 | 0.35±0.03 | 0.34±0.01 | 0.33±0.03 | 0.944 | 0.452 | 0.849 | 0.871 |
| **20:0** | 0.04±0.01 | 0.03±0.00 | 0.03±0.00 | 0.04±0.01 | 0.04±0.01 | 0.04±0.01 | 0.03±0.01 | 0.03±0.01 | 0.589 | 0.651 | 0.588 | 0.353 |
| **22:0** | 0.01±0.00 | 0.01±0.00 | 0.01±0.00 | 0.01±0.00 | 0.02±0.00 | 0.01±0.00 | 0.01±0.00 | 0.01±0.00 | 0.297 | 0.835 | 0.314 | 0.185 |
| **saturated FA** | 2.38±0.04 | 2.36±0.05 | 2.38±0.19 | 2.36±0.05 | 2.46±0.05 | 2.48±0.19 | 2.45±0.06 | 2.39±0.19 | 0.864 | 0.159 | 0.915 | 0.944 |
| **16:1 n-7** | **0.38±0.00** | **0.38±0.01** | **0.38±0.03** | **0.38±0.00** | **0.39±0.01** | **0.41±0.03** | **0.41±0.02** | **0.40±0.03** | 0.415 | **0.029^⭘^** | 0.781 | 0.860 |
| **18:1 (n-9)+(n-7)+(n-5)** | **4.36±0.08** | **4.52±0.08** | **4.49±0.34** | **4.51±0.09** | **4.68±0.11** | **4.51±0.35** | **4.29±0.07** | **4.23±0.38** | 0.351 | 0.650 | 0.556 | 0.149 |
| **20:1 (n-9)+(n-7)** | **0.86±0.02** | **0.88±0.02** | **0.86±0.06** | **0.88±0.03** | **0.91±0.03** | **0.96±0.06** | **0.92±0.02** | **0.91±0.07** | 0.201 | **0.011^⭘^** | 0.549 | 0.820 |
| **22:1(n-11)+(n-9)+(n-7)** | **0.79±0.02** | **0.78±0.02** | **0.76±0.06** | **0.76±0.01** | **0.79±0.05** | **0.85±0.04** | **0.84±0.01** | **0.81±0.05** | 0.059 | **0.003^⭘^** | 0.517 | 0.356 |
| **24:1 n-9** | **0.06±0.01** | **0.06±0.00** | **0.06±0.00** | **0.06±0.00** | **0.06±0.00** | **0.07±0.01** | **0.07±0.01** | **0.06±0.01** | 0.299 | **0.050^⭘^** | 0.620 | 0.421 |
| **monounsaturated** **FA** | 6.45±0.05 | 6.63±0.14 | 6.56±0.49 | 6.59±0.12 | 6.84±0.18 | 6.80±0.49 | 6.53±0.11 | 6.41±0.55 | 0.715 | 0.529 | 0.677 | 0.490 |
| **16:2 n-4** | 0.01±0.00 | 0.01±0.00 | 0.01±0.00 | 0.01±0.00 | 0.02±0.00 | 0.01±0.00 | 0.02±0.01 | 0.02±0.01 | 0.662 | 0.175 | 0.713 | 0.665 |
| **18:2 n-6** | 1.58±0.04 | 1.66±0.05 | 1.65±0.12 | 1.64±0.05 | 1.72±0.04 | 1.65±0.09 | 1.58±0.04 | 1.56±0.12 | 0.277 | 0.836 | 0.578 | **0.093** |
| **18:3 n-6** | 0.01±0.00 | 0.01±0.00 | 0.01±0.00 | 0.01±0.00 | 0.02±0.00 | 0.01±0.00 | 0.01±0.00 | 0.01±0.00 | 0.297 | 0.835 | 0.314 | 0.185 |
| **20:2 n-6** | 0.14±0.01 | 0.14±0.01 | 0.14±0.01 | 0.14±0.00 | 0.15±0.01 | 0.15±0.01 | 0.14±0.00 | 0.09±0.07 | 0.145 | 0.304 | 0.166 | 0.151 |
| **20:3 n-6** | 0.03±0.00 | 0.03±0.00 | 0.03±0.01 | 0.03±0.00 | 0.03±0.00 | 0.03±0.00 | 0.03±0.00 | 0.03±0.00 | 0.472 | 0.430 | 0.550 | 0.289 |
| **20:4 n-6** | 0.03±0.00 | 0.03±0.00 | 0.03±0.00 | 0.02±0.01 | 0.03±0.01 | 0.03±0.00 | 0.03±0.00 | 0.03±0.00 | 0.545 | 0.994 | 0.467 | 0.358 |
| **PUFA (n-6) FA** | 1.79±0.04 | 1.88±0.05 | 1.87±0.14 | 1.86±0.05 | 1.93±0.05 | 1.87±0.11 | 1.79±0.04 | 1.77±0.14 | 0.376 | 0.845 | 0.637 | 0.138 |
| **18:3 n-3** | 0.46±0.01 | 0.48±0.02 | 0.48±0.03 | 0.48±0.02 | 0.51±0.02 | 0.48±0.02 | 0.45±0.01 | 0.45±0.04 | 0.118 | 0.736 | 0.369 | **0.040** |
| **18:4 n-3** | 0.10±0.00 | 0.10±0.01 | 0.10±0.01 | 0.09±0.01 | 0.10±0.02 | 0.10±0.01 | 0.10±0.01 | 0.10±0.01 | 0.800 | 0.321 | 0.939 | 0.537 |
| **20:3 n-3** | 0.04±0.00 | 0.04±0.01 | 0.04±0.00 | 0.04±0.00 | 0.05±0.00 | 0.04±0.00 | 0.04±0.00 | 0.04±0.00 | 0.546 | 0.309 | 0.588 | 0.413 |
| **20:4 n-3** | 0.11±0.00 | 0.11±0.01 | 0.11±0.01 | 0.10±0.00 | 0.11±0.01 | 0.12±0.01 | 0.11±0.01 | 0.10±0.01 | 0.369 | 0.706 | 0.241 | 0.359 |
| **20:5 n-3 (EPA)** | 0.28±0.01 | 0.27±0.01 | 0.27±0.02 | 0.26±0.01 | 0.28±0.00 | 0.29±0.01 | 0.29±0.01 | 0.28±0.02 | 0.073 | **0.003^⭘^** | 0.780 | 0.418 |
| **21:5 n-3** | 0.01±0.00 | 0.01±0.00 | 0.01±0.00 | 0.01±0.00 | 0.02±0.00 | 0.01±0.00 | 0.01±0.00 | 0.01±0.00 | 0.297 | 0.835 | 0.314 | 0.185 |
| **22:5 n-3** | 0.12±0.01 | 0.12±0.00 | 0.12±0.00 | 0.11±0.00 | 0.12±0.00 | 0.13±0.01 | 0.13±0.01 | 0.12±0.01 | 0.510 | **0.049^⭘^** | 0.842 | 0.772 |
| **22:6 n-3 (DHA)** | 0.69±0.03 | 0.67±0.02 | 0.66±0.04 | 0.64±0.00 | 0.68±0.01 | 0.70±0.03 | 0.69±0.01 | 0.70±0.05 | 0.176 | **0.026^⭘^** | 0.659 | 0.269 |
| **PUFA (n-3) FA** | 1.82±0.05 | 1.80±0.06 | 1.79±0.12 | 1.75±0.03 | 1.85±0.05 | 1.87±0.08 | 1.83±0.03 | 1.82±0.14 | 0.652 | 0.105 | 0.631 | 0.946 |
| **total-PUFA FA** | 3.62±0.08 | 3.69±0.12 | 3.68±0.26 | 3.62±0.07 | 3.80±0.09 | 3.76±0.19 | 3.64±0.08 | 3.61±0.29 | 0.813 | 0.471 | 0.663 | 0.705 |
| **omega-6/omega-3 ratio** | **0.98±0.02^a^** | **1.04±0.01^b^** | **1.04±0.02^b^** | **1.06±0.02^b^** | **1.04±0.01^b^** | **1.00±0.02^a^** | **0.98±0.01^a^** | **0.97±0.01^a^** | **0.000** | **0.000^I^** | 0.590 | **0.000** |
| **EPA + DHA** | **0.96±0.03** | **0.94±0.03** | **0.93±0.06** | **0.90±0.01** | **0.96±0.01** | **0.99±0.04** | **0.99±0.01** | **0.98±0.07** | 0.141 | **0.011^⭘^** | 0.690 | 0.368 |
| **identified FA** | 12.46±0.15 | 12.68±0.30 | 12.61±0.94 | 12.57±0.25 | 13.09±0.31 | 13.04±0.87 | 12.61±0.24 | 12.41±1.02 | 0.818 | 0.410 | 0.732 | 0.685 |
| **unidentified FA** | 0.53±0.08 | 0.48±0.02 | 0.50±0.03 | 0.54±0.07 | 0.50±0.04 | 0.53±0.02 | 0.55±0.05 | 0.52±0.07 | 0.771 | 0.651 | 0.826 | 0.437 |

*Values in the same row with different small letter are significantly different (p<0.05), following Duncan post hoc test.

Supplementary-Table 10 Biometrics, welfare, and technical fillet quality of Atlantic salmon post smolts, fed diets with variable source (organic or inorganic) and levels of trace minerals*

|  | **IM1** | **IM2** | **IM3** | **IM4** | **p-value*** | **OM1** | **OM2** | **OM3** | **OM4** | **p-value*** |
| --- | --- | --- | --- | --- | --- | --- | --- | --- | --- | --- |
| **Biometrics** |  |  |  |  |  |  |  |  |  |  |
| BW fish | 637.3±5.9 | 630.2±4 | 629.2±4.9 | 629.7±4.9 | ns | 632±5.1 | 630.3±6.4 | 640.6±4.1 | 646.8±5.5 | ns |
| BW bled out | 629.4±5.9 | 623.2±4 | 619.4±4.9 | 620.1±4.5 | ns | 623.7±5.1 | 619.6±6.3 | 631.4±4.1 | 636.4±5.3 | ns |
| **Gutted BW** | 566.5±5.5 | 564.1±3.7 | 559.9±4.6 | 559.8±3.4 | ns | **565.4±4.5^AB^** | **560.3±6.6^A^** | **573.4±4.4^AB^** | **578.6±5.2^B^** | **0.074** |
| Slaughter yield (%) | 89.1±0.2 | 89.5±0.2 | 89±0.2 | 88.9±0.3 | ns | 89.5±0.1 | 89.2±0.3 | 89.5±0.2 | 89.4±0.2 | ns |
| **Fillet yield, round W (%)** | **69±0.5^b^** | **69.4±0.4^b^** | **68.8±0.6^b^** | **67.4±0.5^a^** | **0.032** | 69.1±0.3 | 68.5±0.4 | 69.3±0.4 | 69.9±0.5 | ns |
| **Fillet yield, Gutted W (%)** | **77.2±0.5^AB^** | **77.5±0.4^B^** | **77.3±0.6^B^** | **75.7±0.5^A^** | **0.068** | 77.3±0.4 | 77±0.4 | 77.4±0.5 | 78.1±0.5 | ns |
| Length (cm) | 35.3±0.2 | 35.3±0.2 | 35.2±0.2 | 35.3±0.1 | ns | 35.5±0.2 | 35.2±0.2 | 35.8±0.2 | 35.8±0.1 | ns |
| Condition factor, round W | 1.44±0.02 | 1.44±0.03 | 1.45±0.02 | 1.43±0.02 | ns | 1.42±0.02 | 1.45±0.02 | 1.4±0.01 | 1.41±0.02 | ns |
| Condition factor, gutted W | 1.28±0.01 | 1.29±0.02 | 1.29±0.02 | 1.27±0.01 | ns | 1.27±0.02 | 1.3±0.02 | 1.25±0.01 | 1.26±0.01 | ns |
| HSI | 1.15±0.03 | 1.07±0.02 | 1.08±0.02 | 1.16±0.05 | ns | 1.07±0.02 | 1.07±0.04 | 1.05±0.02 | 1.1±0.03 | ns |
| **Welfare** |  |  |  |  |  |  |  |  |  |  |
| Cataract, sum | 0.7±0.2 | 0.3±0.1 | 0.3±0.2 | 0.5±0.2 | ns | 0.2±0.1 | 0.4±0.2 | 0.1±0.1 | 0.7±0.3 | ns |
| Fin score (0-3) | 1.7±0.2 | 1±0.3 | 1.7±0.3 | 1.7±0.2 | ns | 1.2±0.2 | 1.5±0.2 | 1.6±0.3 | 1.3±0.2 | ns |
| Liver score (0-5) | 3.6±0.1 | 3.2±0.2 | 3.4±0.2 | 3.6±0.2 | ns | 3.3±0.2 | 3.5±0.1 | 3.6±0.1 | 3.7±0.2 | ns |
| Viscera fat score(0-5) | 2.4±0.2 | 2.6±0.2 | 2.4±0.3 | 2.3±0.2 | ns | 2.3±0.2 | 2.1±0.2 | 1.9±0.1 | 2.2±0.2 | ns |
| **Bone strength, force at 15% thickness (N)** | 41±2.1 | 39.6±1.3 | 41.1±1.6 | 38.4±1.4 | ns | **39.1±1.3^ab^** | **36.7±1.8^a^** | **41.2±1.5^b^** | **42.5±1.3^b^** | **0.039** |
| Bone strength, force at 50% thickness (N) | 74±3.3 | 70.5±3.4 | 72.2±3.1 | 76.9±2.3 | ns | 76±2.4 | 70.8±2.2 | 77.5±2.9 | 78.9±2.7 | ns |
| **Technical quality** |  |  |  |  |  |  |  |  |  |  |
| **pH** | 6.24±0.02 | 6.29±0.01 | 6.26±0.02 | 6.25±0.02 | ns | 6.26±0.02 | 6.25±0.02 | 6.24±0.01 | 6.27±0.02 | ns |
| Gaping, fresh fillet (0-5) | 0.8±0.2 | 0.3±0.1 | 0.5±0.2 | 0.6±0.2 | ns | 0.7±0.2 | 0.6±0.2 | 0.8±0.2 | 0.5±0.2 | ns |
| **Gaping after thawing (0-5)** | 1.1±0.3 | 1.4±0.3 | 0.5±0.2 | 1.1±0.4 | ns | **0.8±0.2^a^** | **1.1±0.3^ab^** | **1.9±0.3^b^** | **0.5±0.3^a^** | **0.0079** |
| Fillet firmness, breaking force (N) | 3.3±0.1 | 3.4±0.1 | 3.7±0.2 | 3.5±0.1 | ns | 3.5±0.1 | 3.5±0.1 | 3.3±0.1 | 3.6±0.1 | ns |
| **Fillet firmness, total work (N*sec)** | **52.8±2.3^a^** | **53.7±1.7^a^** | **59.9±1.9^b^** | **52.8±2^ab^** | **0.012** | 54.6±1 | 52.4±1.4 | 54.6±1.1 | 59.2±2.2 | ns |
| **Liquid loss (%)** | **4.1±0.3^a^** | **4.4±0.3^a^** | **3.7±0.3^a^** | **4.9±0.3^b^** | **0.035** | **4.6±0.2^b^** | **3.7±0.3^a^** | **4.7±0.2^b^** | **3.6±0.2^a^** | **0.0014** |
| Skin strength, puncture force (N) | 2.5±0.1 | 2.7±0.2 | 2.8±0.2 | 2.6±0.2 | ns | 2.8±0.1 | 2.8±0.1 | 2.8±0.2 | 2.9±0.2 | ns |
| SalmonFan, score | 21.9±0.1 | 22.2±0.2 | 22.1±0.2 | 22±0.2 | ns | 21.8±0.2 | 22.1±0.2 | 22±0.1 | 22.2±0.1 | ns |
| Muscle_L | 42.5±0.4 | 42.6±0.3 | 42.8±0.4 | 42.9±0.4 | ns | 42.3±0.3 | 42.6±0.3 | 42.1±0.3 | 42.2±0.3 | ns |
| Muscle_a | 6.6±0.3 | 7.3±0.3 | 6.9±0.3 | 6.6±0.3 | ns | 6.8±0.3 | 6.6±0.4 | 6.6±0.3 | 6.6±0.3 | ns |
| Muscle_b | 9.6±0.3 | 10.8±0.3 | 10.6±0.4 | 10.1±0.4 | ns | 10.3±0.4 | 10.1±0.5 | 9.6±0.4 | 9.8±0.3 | ns |
| Muscle_Chroma | 11.7±0.4 | 13±0.4 | 12.7±0.5 | 12.1±0.5 | ns | 12.4±0.5 | 12.1±0.6 | 11.7±0.5 | 11.8±0.4 | ns |
| **Skin_L** | **77.6±1.4^b^** | **71.2±1.8^a^** | **71.2±1.9^a^** | **76.8±1.3^b^** | **0.008** | 76±1.4 | 79.3±1.2 | 76.6±1.8 | 79.5±1.1 | ns |
| Skin_a | -0.8±0.2 | -0.7±0.2 | -1±0.2 | -0.6±0.2 | ns | -0.1±0.2 | -0.5±0.2 | -0.7±0.2 | -0.5±0.2 | ns |
| Skin_b | 2±0.4 | 1.1±0.4 | 2.1±0.4 | 1.8±0.4 | ns | 0.8±0.4 | 1.2±0.5 | 0.9±0.4 | 0.7±0.3 | ns |
| Skin_Chroma | 2.3±0.3 | 1.9±0.3 | 2.6±0.3 | 2.2±0.4 | ns | 1.6±0.2 | 1.9±0.4 | 1.8±0.3 | 1.4±0.2 | ns |

*Values in the same row with different small letter are significantly different (p<0.05), whereas those with different capital letter show tendency for difference (p<0.1) following Duncan post hoc test

Supplementary-Table 11 Comparison of Atlantic salmon post-smolt biometrics, welfare and quality scores fed either inorganic or organic trace mineral premix in the diet^*^.

|  | **IM** | **OM** | **p-value** |
| --- | --- | --- | --- |
| Sex | 0.5 ± 0.1 | 0.6 ± 0.1 | ns |
| **Biometrics** |  |  |  |
| Body weigth (g) | 631.6 ± 2.5 | 637.4 ± 2.7 | ns |
| Body weigth, bled (g) | 623 ± 2.4 | 627.8 ± 2.7 | ns |
| **Blood loss (%)** | **1.4 ± 0.1** | **1.5 ± 0** | **0.069** |
| **Gutted body weight (g)** | **562.5 ± 2.2** | **569.6 ± 2.7** | **0.043** |
| **Slaughter yield (%)** | **89.1 ± 0.1** | **89.4 ± 0.1** | **0.000** |
| **Fillet yield, round weight (%)** | **68.6 ± 0.3** | **69.2 ± 0.2** | **0.084** |
| Fillet yield, gutted weight (%) | 76.9 ± 0.3 | 77.4 ± 0.2 | ns |
| **Length (cm)** | **35.3 ± 0.1** | **35.6 ± 0.1** | **0.033** |
| Condition factor, round weight | 1.44 ± 0.01 | 1.42 ± 0.01 | ns |
| Condition factor, gutted weight | 1.28 ± 0.01 | 1.27 ± 0.01 | ns |
| Liver weight (g) | 7 ± 0.1 | 6.8 ± 0.1 | ns |
| **HSI** | **1.11 ± 0.02** | **1.07 ± 0.02** | **0.060** |
| **Welfare** |  |  |  |
| Left eye cataract score | 0.2 ± 0 | 0.1 ± 0.1 | ns |
| Right eye cataract score | 0.3 ± 0.1 | 0.2 ± 0.1 | ns |
| Cataract, sum | 0.5 ± 0.1 | 0.4 ± 0.1 | ns |
| Liver score (0-5) | 3.5 ± 0.1 | 3.5 ± 0.1 | ns |
| **Viscera fat score (0-5)** | **2.4 ± 0.1** | **2.1 ± 0.1** | **0.049 (one-sided)** |
| Fin score (0-3) | 1.5 ± 0.1 | 1.4 ± 0.1 | ns |
| **Quality** |  |  |  |
| pH | 6.26 ± 0.01 | 6.25 ± 0.01 | ns |
| Nice_fillet_g | 216.8 ± 1.2 | 220.5 ± 1.2 | ns |
| Gaping, fresh fillet (0-5) | 0.6 ± 0.1 | 0.7 ± 0.1 | ns |
| Gaping after thawing (0-5) | 1.1 ± 0.2 | 1.1 ± 0.2 | ns |
| Water holding capacity (%) | 4.3 ± 0.2 | 4.2 ± 0.1 | ns |
| Fillet firmness, breaking force (N) | 3.5 ± 0.1 | 3.5 ± 0.1 | ns |
| Area0_Fb_nks | 6.1 ± 0.2 | 6.1 ± 0.1 | ns |
| Fillet firmness, total work (N*sec) | 54.8 ± 1 | 55.2 ± 0.8 | ns |
| mm_nks | 15.5 ± 0.1 | 15.4 ± 0.1 | ns |
| Skin strength, puncture force (N) | 2.7 ± 0.1 | 2.8 ± 0.1 | ns |
| Max_force_X | 88.4 ± 1.9 | 87.6 ± 1.6 | ns |
| Strain_Max_force_X | 58.4 ± 1 | 55.4 ± 0.9 | ns |
| F70_X | 73.3 ± 3 | 71.6 ± 2.3 | ns |
| Area_70perc_sec_X | 98.1 ± 2 | 99.8 ± 1.8 | ns |
| Area_70perc_mm_X | 194.9 ± 3.9 | 198.1 ± 3.7 | ns |
| Area_15perc_sec_X | 8.4 ± 0.1 | 8.4 ± 0.1 | ns |
| Area_15perc_mm_X | 16.3 ± 0.2 | 16.3 ± 0.2 | ns |
| Bone strength, force at 15% thickness (N) | 40.1 ± 0.8 | 39.9 ± 0.8 | ns |
| Area_50perc_sec_X | 61.9 ± 1.1 | 63.2 ± 1.1 | ns |
| Area_50perc_mm_X | 122.5 ± 2.2 | 125.2 ± 2.2 | ns |
| Bone strength, force at 50% thickness(N) | 73.3 ± 1.5 | 75.8 ± 1.3 | ns |
| Thickness_X | 5.1 ± 0 | 5.1 ± 0 | ns |
| Vekt_thaw | 207.6 ± 1.2 | 211.6 ± 1.1 | ns |
| SalmonFan, score | 22.1 ± 0.1 | 22 ± 0.1 | ns |
| **Muscle_L** | **42.7 ± 0.2** | **42.3 ± 0.1** | **0.098** |
| Muscle_a | 6.8 ± 0.2 | 6.7 ± 0.2 | ns |
| Muscle_b | 10.3 ± 0.2 | 9.9 ± 0.2 | ns |
| Muscle_Hue | 56.6 ± 0.4 | 56.3 ± 0.3 | ns |
| Muscle_Chroma | 12.4 ± 0.2 | 12 ± 0.3 | ns |
| **Skin_L** | **74.2 ± 0.9** | **77.9 ± 0.7** | **0.002** |


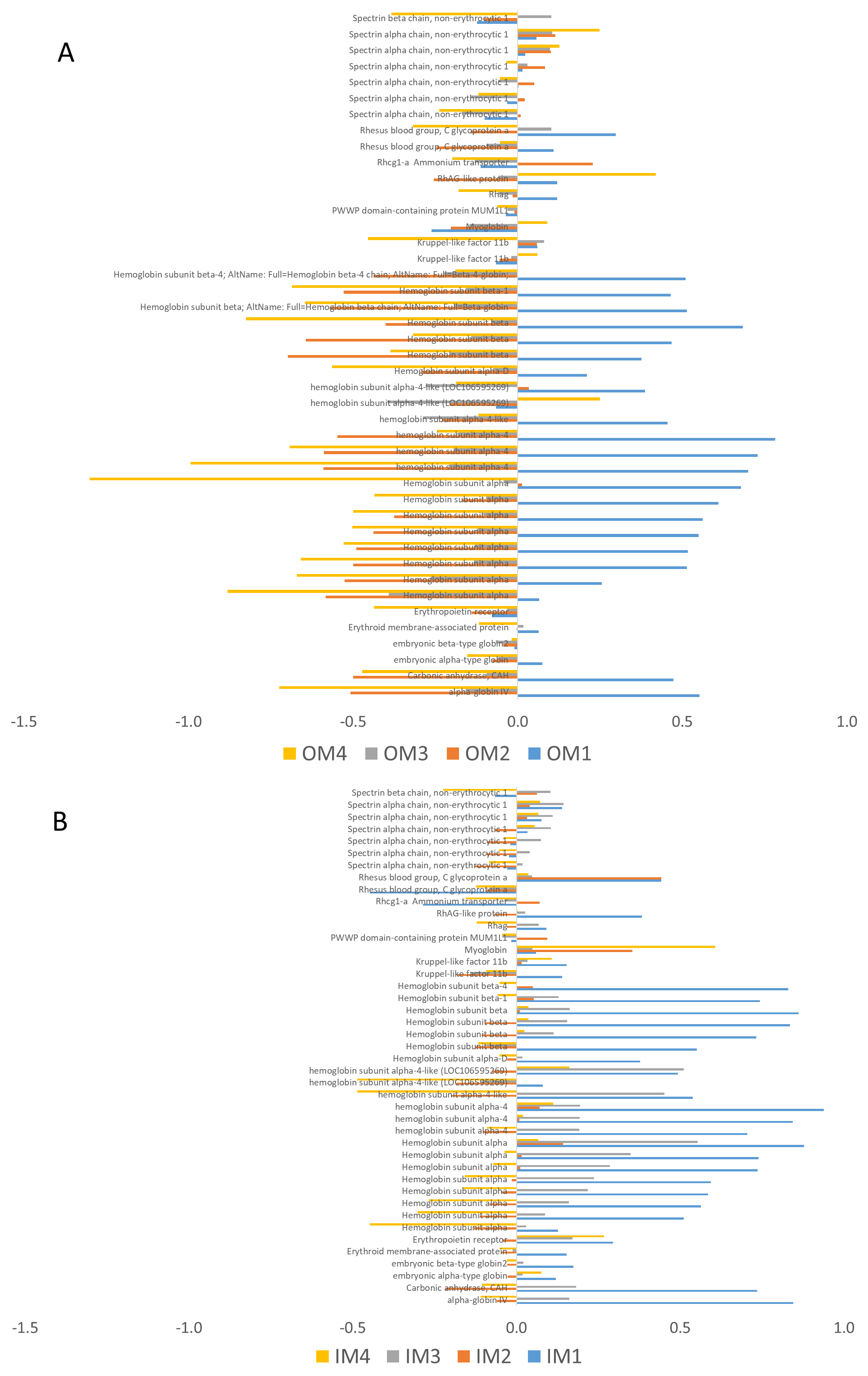


Supplementary - Figure 1 Relative expression of globin metabolism related genes in Atlantic salmon post smolt midguts, fed diets with various levels of trace minerals in organic (A) and inorganic (B) form
